# Rhodium-catalyzed reductive carbonylation of aryl iodides to arylaldehydes with syngas

**DOI:** 10.3762/bjoc.16.61

**Published:** 2020-04-08

**Authors:** Zhenghui Liu, Peng Wang, Zhenzhong Yan, Suqing Chen, Dongkun Yu, Xinhui Zhao, Tiancheng Mu

**Affiliations:** 1 School of Pharmaceutical and Materials Engineering, Taizhou University, Taizhou 318000, Zhejiang, China; 2 Beijing National Laboratory for Molecular Sciences, CAS Research/Education Center for Excellence in Molecular Sciences, Institute of Chemistry, Chinese Academy of Sciences, Beijing 100190, China; 3 Key Laboratory of Green Chemical Media and Reactions, Ministry of Education, School of Chemistry and Chemical Engineering, Henan Normal University, Xinxiang 453007, Henan, China,; 4 Department of Chemistry, Renmin University of China, Beijing 100872, China

**Keywords:** cost-effective ligand, industrial catalysis, reductive carbonylation, rhodium catalyst, syngas

## Abstract

The reductive carbonylation of aryl iodides to aryl aldehydes possesses broad application prospects. We present an efficient and facile Rh-based catalytic system composed of the commercially available Rh salt RhCl_3_·3H_2_O, PPh_3_ as phosphine ligand, and Et_3_N as the base, for the synthesis of arylaldehydes via the reductive carbonylation of aryl iodides with CO and H_2_ under relatively mild conditions with a broad substrate range affording the products in good to excellent yields. Systematic investigations were carried out to study the experimental parameters. We explored the optimal ratio of Rh salt and PPh_3_ ligand, substrate scope, carbonyl source and hydrogen source, and the reaction mechanism. Particularly, a scaled-up experiment indicated that the catalytic method could find valuable applications in industrial productions. The low gas pressure, cheap ligand and low metal dosage could significantly improve the practicability in both chemical researches and industrial applications.

## Introduction

The exploration of environmentally friendly and highly effective synthetic methods has been a significant goal of research [1–5]. In this aspect, effective catalytic systems and organometallic chemistry are suitable technologies to accomplish these goals. Carbonylation processes are important transformations in the refinement and reprocessing of readily available industrial raw materials into more functionalized products. These processes generally utilize carbon monoxide (CO), currently the most important C1 building block used in numerous industrial carbonylation processes [6–8] and widely applied in industrial productions [9–12]. Carbonylations are one of the industrial core technologies for transforming various bulk chemicals into useful products that are used in our daily life. Carbonylation reactions, together with polymerizations and oxidations, constitute the largest industrial applications in the field of homogeneous catalysis, and substantial value-added bulk and fine chemicals are available through this technology [13]. In spite of the existing plentiful progress in this conversion, the exploitation of advanced and more effective catalytic systems to the activity and to widen the range of substrates is crucial for new practical applications.

Syngas is a mixture of CO and H_2_, which is cheap, abundant and widely used in chemical industry productions [14–16]. In spite of its comprehensive utilization in industry, reactions involving CO are relatively seldom employed in fine chemicals syntheses. This could be due to the general difficulty of using gases as raw materials and the requirement of high-pressure equipment. In addition, relatively little attention has been paid to carbonylation chemistry using CO in academic research. Also, H_2_ as representative clean energy source is far more environmentally friendly than other frequently used hydrogen sources like hydrosilanes [17], tributyltin hydride (Bu_3_SnH) (often used in natural product syntheses) [18–20] and hydroboranes [21–23], since the only byproduct is water. The production, storage and use of H_2_ received much attention and plentiful achievements promoted the application of H_2_ into more and more chemistry researches and industrial productions [24–27].

Aromatic aldehydes are highly valuable organic compounds that are widely employed as indispensable building blocks in numerous areas of chemistry, especially for the preparation of biologically active molecules or their intermediates [28–29]. Generally, aromatic aldehydes are synthesized by Reimer–Tiemann, Gattermann–Koch, Vielsmeier–Haag, or Duff reactions and so forth. Unfortunately, these reactions usually use auxiliary reagents and thus generate large amounts of industrial waste and other side products. Particularly, the reductive carbonylation of aryl iodides to produce arylaldehydes with CO and H_2_ was seldom reported. Some homogeneous and heterogeneous catalytic systems based on palladium species using CO and H_2_ to complete the reductive carbonylation of aryl halogens to arylaldehydes have been developed. The homogeneous systems included Pd(OAc)_2_ with propyl di-*tert*-butylphosphinite ligand [30], Pd(acac)_2_ with dppm ligand [31], Pd(OAc)_2_ with CataCXium A ligand [32], and all of the three systems employed TMEDA as the base and toluene as the solvent. The heterogeneous systems contained: PdO/Co_3_O_4_ with K_2_CO_3_ [33], MCM-41-S-PdCl_2_ [34], and MCM-41-2P-PdCl_2_ [35]. However, the aforementioned systems often suffer from high toxicity of solvents, high pressure of gases or high reaction temperatures, which make these protocols inapplicable for large scale applications. Actually, in 2004, Eliseev et al. reported a catalytic system based on RhCl(CO)(PPh_3_)_2_ to achieve the conversion of iodobenzene to benzaldehyde in toluene using CO and H_2_ [36]. We sought for a commercially available Rh salt for this conversion at lower cost and higher potential for practical application.

In this work, we established a catalytic system composed of RhCl_3_·3H_2_O and PPh_3_, which allows the reductive carbonylation of aryl iodides using CO and H_2_ in the presence of Et_3_N as the base at 90 °C. In addition, the reported catalytic system demonstrates high catalytic activity affording the arylaldehydes in good to excellent yields, displays high functional-group tolerance, and broad substrate scope. In particular, the catalytic system could be applied in a scaled-up experiment and thus has potential for applications in industrial productions. The reaction mechanism study revealed that RhCl_3_·3H_2_O reacts with PPh_3_ to form RhCl(PPh_3_)_3_, which is able to activate C–I bonds in aryl iodides realizing the insertion of CO and hydrogenolysis with H_2_. The final trapping of HI by the base Et_3_N regenerates the catalyst to complete the reaction cycle. As far as we know, this is the first time that commercially available Rh salts with PPh_3_ as the ligand were utilized to complete the conversion of aryl iodides into arylaldehydes using CO and H_2_ with systematic researches. Considering the efficiency and generality, this catalytic system is expected to powerfully influence both laboratory research and chemical industry by offering a practical synthetic tool for the conversion of aryl iodides to arylaldehydes.

## Results and Discussion

### Effects of Rh species

Rhodium salts coordinated with proper ligands have been reported to be able to realize the activation of CO and H_2_ and thus might achieve the reductive carbonylation of aryl iodides to afford aromatic aldehydes [37–40]. Rh salts generally play vital roles in the catalytic results. Therefore, we tested 20 different Rh salts and the results are summarized in Table 1. The initial reaction conditions were set as PhI (1 mmol), Rh species (2.5 mol %), PPh_3_ (10 mol %), Et_3_N (1.2 mmol), DMA (2 mL), CO/H_2_ (5 bar:5 bar), 90 °C and 12 h. As can be seen from Table 1, most of the Rh salts gave unsatisfactory results with yields of benzaldehyde below 50%. However, three Rh salts provided yields over 50%, namely [RhCl(CO)_2_]_2_ (64%), RhCl_3_·3H_2_O (97%) and RhI_3_ (89%) (Table 1, entries 1, 18 and 20). Interestingly, RhCl_3_·3H_2_O and RhI_3_ performed well, whereas RhCl_3_ and RhBr_2_·2H_2_O afforded the product in very low yield (Table 1, entries 17–20). This strongly implied that not only the valence state of Rh and the species of anions were crucial for the conversion (even if all with halogen anions), but also structural differences (e.g., crystal water, RhCl_3_ vs RhCl_3_·3H_2_O) of analogous Rh salts played an important role in the catalytic synthesis of benzaldehyde. Among all tested rhodium salts, RhCl_3_·3H_2_O afforded benzaldehyde in the highest yield and was chosen as the most suitable Rh salt for the conversion. In addition, in spite of low yields, Rh species with valence states of 0, +1, +2 or +3 all promoted the reaction at least to some extent. It is worth noting that as a good leaving group, I^−^ tends to leave in an alkaline environment, and dehalogenation and direct coupling products (namely, benzene and biphenyl) were detected as the main byproducts by GC–MS. This observation also explains why the conversions were always higher than the yields.

**Table 1:** Rhodium-catalyzed reductive carbonylation of iodobenzene with CO and H_2_ to afford benzaldehyde: effects of the Rh species^a^.

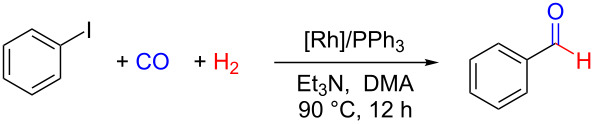			

Entry	Rh species	Conversion [%]^b^	Yield [%]^b^

1	[RhCl(CO)_2_]_2_	89	64
2	RhCl(CO)(PPh_3_)_2_	37	31
3	Rh(acac)(CO)_2_	21	15
4	[RhCl(COD)]_2_	16	14
5	Rh(MeCN)_2_(COD)BF_4_	15	12
6	Rh(COD)BF_4_	27	21
7	Rh(COD)_2_OTf	21	18
8	[Rh(OMe)(COD)]_2_	23	19
9	Rh(OAc)_2_	71	33
10	[Rh(CF_3_COO)]_2_	43	12
11	[Rh(CH_3_(CH_2_)_6_CO_2_)_2_]_2_	29	22
12	Rh(ethylene)_2_(acac)	48	42
13	[Rh(ethylene)_2_Cl]_2_	42	36
14	Rh(norbornadiene)_2_BF_4_	25	10
15	[Cp*RhCl_2_]_2_	51	46
16	Rh/C	38	21
17	RhCl_3_	22	18
18	RhCl_3_·3H_2_O	100	97
19	RhBr_2_·2H_2_O	31	28
20	RhI_3_	92	89

^a^Standard conditions: PhI (1 mmol), Rh species (2.5 mol %), PPh_3_ (10 mol %), Et_3_N (1.2 mmol), DMA (2 mL), CO/H_2_ (5 bar:5 bar), 90 °C, 12 h. ^b^Determined by GC using dodecane as an internal standard.

### Effects of ligands

Ligands play a decisive role in adjusting the catalytic ability of metal cations [12–13,41–51]. Different ligands coordinating with the same metal cations could make a difference between full conversion with nearly quantitative yields and no reactions. To obtain the optimized conditions, 13 kinds of ligands were tested and their structures are included in Table 2. In accordance with the expectations, only PPh_3_ was effective and afforded benzaldehyde in 97% yield, whereas the majority of the other ligands did not afford any product (Table 2). Therefore, PPh_3_ was selected as the proper ligand employed in the subsequent reactions.

**Table 2:** Rhodium-catalyzed reductive carbonylation of iodobenzene with CO and H_2_ to afford benzaldehyde: effects of the ligands^a^.

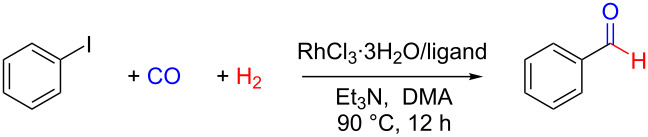			

Entry	Ligand	Conversion [%]^b^	Yield [%]^b^

1	PP_3_	8	0
2	triphos	5	0
3	dpp-BINAP	12	0
4	dpp-OPh	7	3
5	dppb	6	0
6	dppe	11	0
7	P(PhF_5_)_3_	16	0
8	P(4-FPh)_3_	13	0
9	Cydpp	8	2
10	Bipy	9	4
11	DBU	3	0
12	Im	2	0
13	PPh_3_	100	97

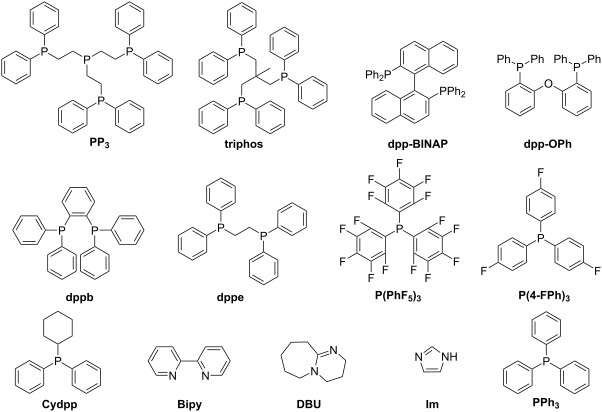
^a^Standard conditions: PhI (1 mmol), RhCl_3_·3H_2_O (2.5 mol %), ligand (10 mol %), Et_3_N (1.2 mmol), DMA (2 mL), CO/H_2_ (5 bar:5 bar), 90 °C, 12 h. ^b^Determined by GC using dodecane as an internal standard.

### Effects of bases

**Figure 1:**
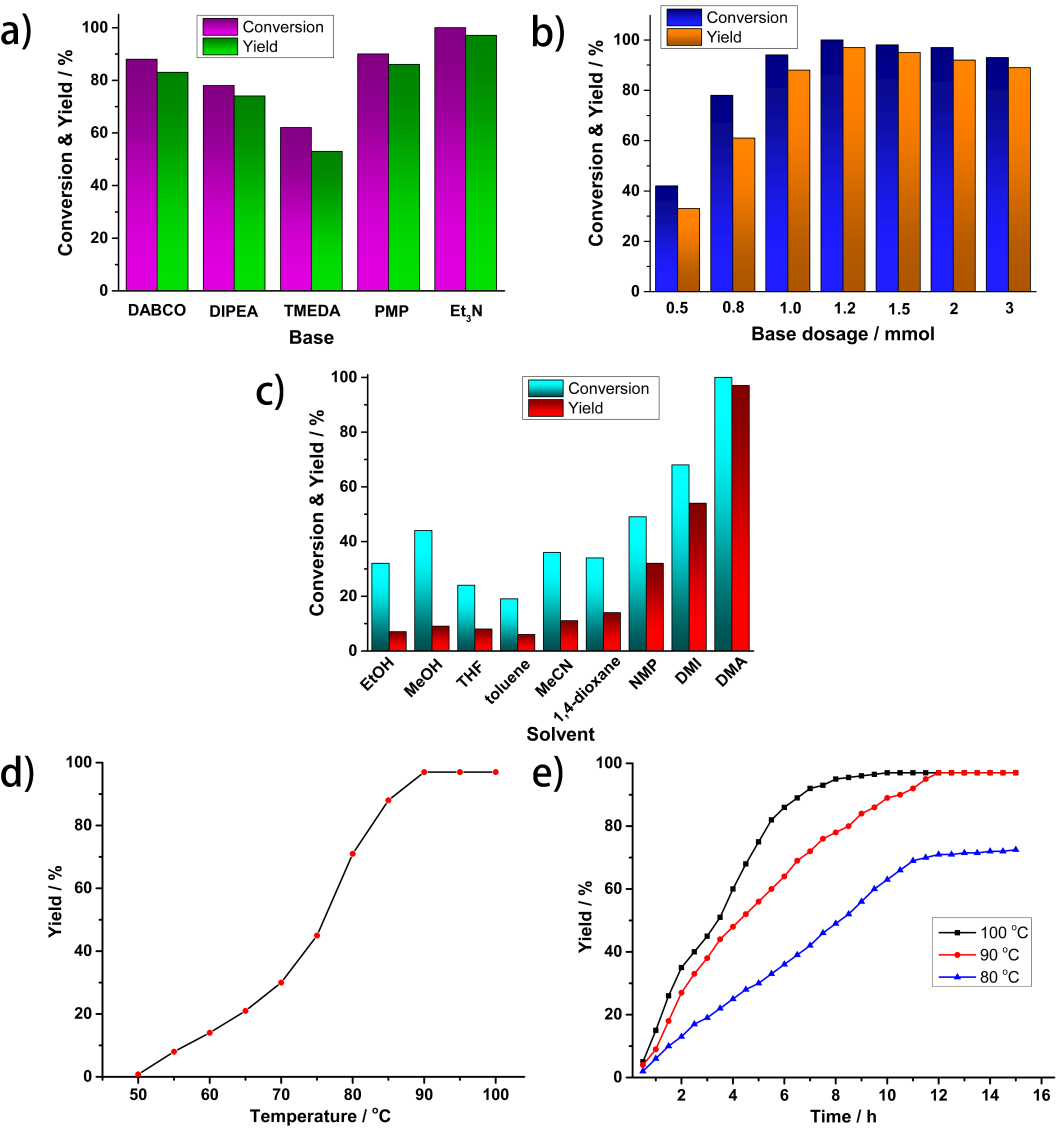
Rhodium-catalyzed reductive carbonylation of iodobenzene with CO and H_2_ to afford benzaldehyde. a) Effects of the tested bases; b) effects of base amount; c) effects of tested solvents; d) temperature screen; e) yields at different times for reactions performed at 80, 90 and 100 °C (every point represents an average result of three parallel experiments). Conversions and yields were determined by GC using dodecane as an internal standard. Standard conditions: PhI (1 mmol), RhCl_3_·3H_2_O (2.5 mol %), PPh_3_ (10 mol %), base (1.2 mmol, if not stated otherwise), DMA (2 mL, if not stated otherwise), CO/H_2_ (5 bar:5 bar), temperature 90 °C (if not stated otherwise), time 12 h (if not stated otherwise).

As explored in the mechanism study, the acid HI is produced during the reaction process, since H^+^ and I^−^ were generated from the reaction system. In order to make the reaction proceed continuously, HI produced needs to be removed effectively and in time. Five different bases were examined to assess their ability to bind HI. All of them were found suitable for the reaction and provided benzaldehyde with yields higher than 70%, except for TMEDA (53%, Figure 1a). The best results were obtained with Et_3_N, which was selected as the most appropriate base.

The optimized base concentration was explored next and the results are shown in Figure 1b. Since the base held the post of absorber for acids produced during the reaction, sufficient amounts are necessary to ensure high yields. As shown in Figure 1b, amounts of Et_3_N less than 1 mmol led to lower yields of 33% for 0.5 mmol Et_3_N and 61% for 0.8 mmol Et_3_N, respectively. However, a slight excess of Et_3_N (1.2 mmol) allowed the reaction to be completed with a product yield of 97%. In addition, further increasing the amount of the base was not beneficial for the yield of benzaldehyde (1.5 mmol Et_3_N, 95% yield; 2 mmol Et_3_N, 92% yield; 3 mmol Et_3_N, 89% yield). Thus, the appropriate amount of Et_3_N was set at 1.2 mmol.

### Effect of solvents

Solvents act as the reaction media and strongly influence the catalytic reactions [52–53]. We screened nine representative solvents in the catalytic reaction and the results are summarized in Figure 1c. Apparently, protic solvents like EtOH or MeOH and nonpolar solvents like THF or toluene were inappropriate for the reaction leading to yields lower than 10%. Medium polar solvents like MeCN or 1,4-dioxane afforded slightly higher yields but still below 15%. To our delight, polar solvents with high boiling points, NMP, DMI or DMA proved to be more suitable, and afforded benzaldehyde in 32%, 54% and 97% yield, respectively, likely owing to the high boiling point and better CO and H_2_ dissolution ability, highlighting a strong solvent dependency of the system [54–55]. Therefore, DMA was selected as the most suitable solvent for the reaction.

### Effects of temperature and time

To further optimize the performance of the catalytic system, the effect of the reaction temperature was screened in the range of 50 °C to 100 °C. The results revealed that the reaction temperature had an important influence on the catalytic process and the product yield increased with increasing temperature. At low temperature the reaction did not proceed at all, however, at 90 °C or higher, a nearly quantitative yield of benzaldehyde was obtained (Figure 1d). Next, parallel experiments were conducted at three selected temperatures (80 °C, 90 °C, and 100 °C) to study the yields at different reaction times. The results revealed that equally high yields were obtained at 90 °C and 100 °, but the reaction was much faster at 100 °C than at 90 °C (Figure 1e). On the other hand, when performing the reaction at 80 °C, the product yield was lower and required longer times to be reached.

### Effects of pressure of CO and H_2_

The influence of the pressures of CO and H_2_ was explored next. As expected, a low pressure of both, CO (1 atm) and H_2_ (1 atm), resulted in a lower yield of benzaldehyde (24% yield, Table 3, entry 1). When the pressures of both gases were increased to 2, 3, 4, or 5 bar, the yield of benzaldehyde accordingly increased to 51%, 62% 81%, and 97%, respectively (Table 3, entries 2–5). However, further increasing the pressures did not lead to higher yields (Table 3, entries 6 and 7). After that, we examined the proportions of the syngas components on the reaction and it was found that the optimized proportions of syngas were 5 bar CO and 5 bar H_2_ (1:1, (Table 3, entries 8–13).

**Table 3:** Rhodium-catalyzed reductive carbonylation of iodobenzene with CO and H_2_ to afford benzaldehyde: effect of pressures of CO and H_2_^a^.

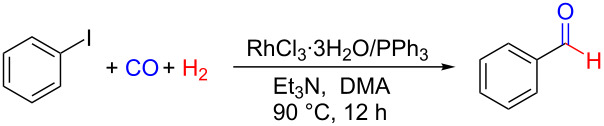				

Entry	*p*(CO) [bar]	*p*(H_2_) [bar]	Conversion [%]^b^	Yield [%]^b^

1	1	1	32	24
2	2	2	63	51
3	3	3	74	62
4	4	4	92	81
5	5	5	100	97
6	6	6	100	97
7	7	7	100	97
8	2	4	72	63
9	4	2	66	58
10	3	6	85	72
11	6	3	74	68
12	4	6	99	94
13	6	4	95	89

^a^Standard conditions: PhI (1 mmol), RhCl_3_·3H_2_O (2.5 mol %), PPh_3_ (10 mol %), Et_3_N (1.2 mmol), DMA (2 mL), CO/H_2_, 90 °C, 12 h. ^b^Determined by GC using dodecane as an internal standard.

### Scaled-up experiment

**Scheme 1:**
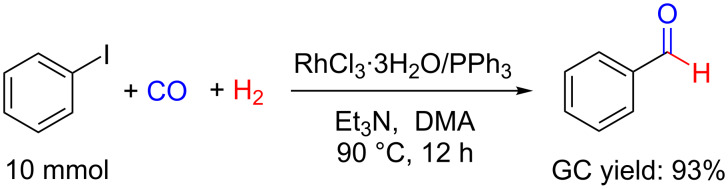
Scaled-up experiment of the reductive carbonylation of iodobenzene to benzaldehyde under the optimized conditions.

Noteworthy, when conducting the reaction at a larger scale (10 mmol), benzaldehyde was obtained with a high yield of 93%, indicating that our system could be suitable for industrial application (Scheme 1).

### Optimal ratio of Rh salt and PPh_3_ ligand and active species participating in the catalytic process

In order to ascertain the optimized ratio of RhCl_3_·3H_2_O and the PPh_3_ ligand, a series of experiments with different concentrations and ratios of RhCl_3_·3H_2_O and PPh_3_ were conducted. At first, the concentration of RhCl_3_·3H_2_O was fixed at 1 mol %. When changing the amount of PPh_3_ from 2 mol % to 5 mol %, the yield gradually increased reaching a maximum yield of 74% at 4 mol % PPh_3_. Further increasing the amount of the ligand resulted in a decrease of the product yield (Table 4, entries 1–4). Next, the concentration of RhCl_3_·3H_2_O was increased to 2 mol % and 2.5 mol %, leading to the same results. In other words, the optimal ratio of RhCl_3_·3H_2_O and the PPh_3_ ligand was always 1:4 (Table 4, entries 5–12). No higher yields (Table 4, entries 13–15) could be obtained by increasing the loadings of RhCl_3_·3H_2_O and PPh_3_.

**Scheme 2:**
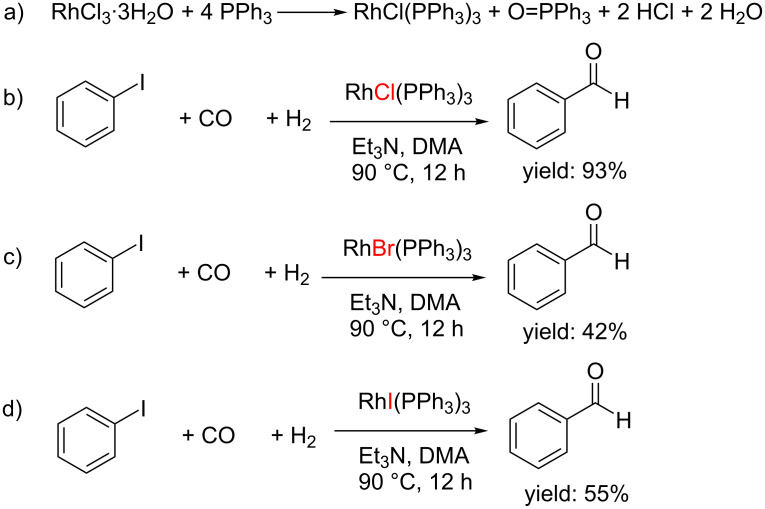
Catalytic species participating in the catalytic process.

The effects of PPh_3_ dosage on the catalytic ability demonstrated that under otherwise identical conditions, a maximum yield of benzaldehyde (97%) was obtained with a 1:4 molar ratio of RhCl_3_·3H_2_O/PPh_3_, while higher or lower concentrations of PPh_3_ both led to decreased yields (Table 4). This indicated that in the active catalytic species the molar ratio of [Rh]:PPh_3_ was exactly 1:3 (not 1:4 because of the consumption of 1 equiv PPh_3_ during the redox process), which might be Rh(PPh_3_)_3_Cl. The mentioned redox reaction can be explained as shown in Scheme 2a. Initially, RhCl_3_·3H_2_O is reduced by 1 equiv PPh_3_ to form a Rh(I) species that subsequently combines with 3 equiv PPh_3_ forming Rh(PPh_3_)_3_Cl. During this process PPh_3_ is oxidized to O=PPh_3_, and HCl and H_2_O are released.

**Table 4:** Rhodium-catalyzed reductive carbonylation of iodobenzene with CO and H_2_ to afford benzaldehyde: effects of dosage of RhCl_3_·3H_2_O and PPh_3_^a^.

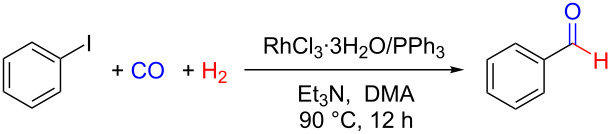				

Entry	RhCl_3_·3H_2_O [mol %]	PPh_3_ [mol %]	Conversion [%]^b^	Yield [%]^b^

1	1	2	35	28
2	1	3	51	47
3	1	4	78	74
4	1	5	72	70
5	2	4	34	31
6	2	6	65	62
7	2	8	92	88
8	2	10	88	84
9	2.5	5	47	42
10	2.5	7.5	81	73
11	2.5	10	100	97
12	2.5	12.5	100	92
13	3	12	100	97
14	4	16	100	97
15	5	20	100	97

^a^Standard conditions: PhI (1 mmol), RhCl_3_·3H_2_O, PPh_3_, Et_3_N (1.2 mmol), DMA (2 mL), CO/H_2_ (5 bar:5 bar), 90 °C, 12 h. ^b^Determined by GC using dodecane as an internal standard.

To test this assumption, we synthesized Rh(PPh_3_)_3_Cl according to the literature [56] on account of no commercial sources and employed it as the catalyst in the reaction instead of RhCl_3_·3H_2_O and PPh_3_ and a 93% yield of benzaldehyde was obtained under otherwise identical reaction conditions (Scheme 2b). This could be an evidence that after the reduction of one equivalent RhCl_3_ complexation with three molecular PPh_3_ takes place forming Rh(PPh_3_)_3_Cl, which participated in the subsequent catalytic process. Herein, Rh(III) is reduced to Rh(I), with concomitant oxidation of PPh_3_ to PPh_3_=O. A quite interesting fact is that Rh(PPh_3_)_3_Br and Rh(PPh_3_)_3_I afforded much lower yields of the aldehyde compared to Rh(PPh_3_)_3_Cl although they share the identical metal center and ligand (Scheme 2c and 2d). This indicated that the anion plays an important role in the catalytic process.

### Substrate scope

**Scheme 3:**
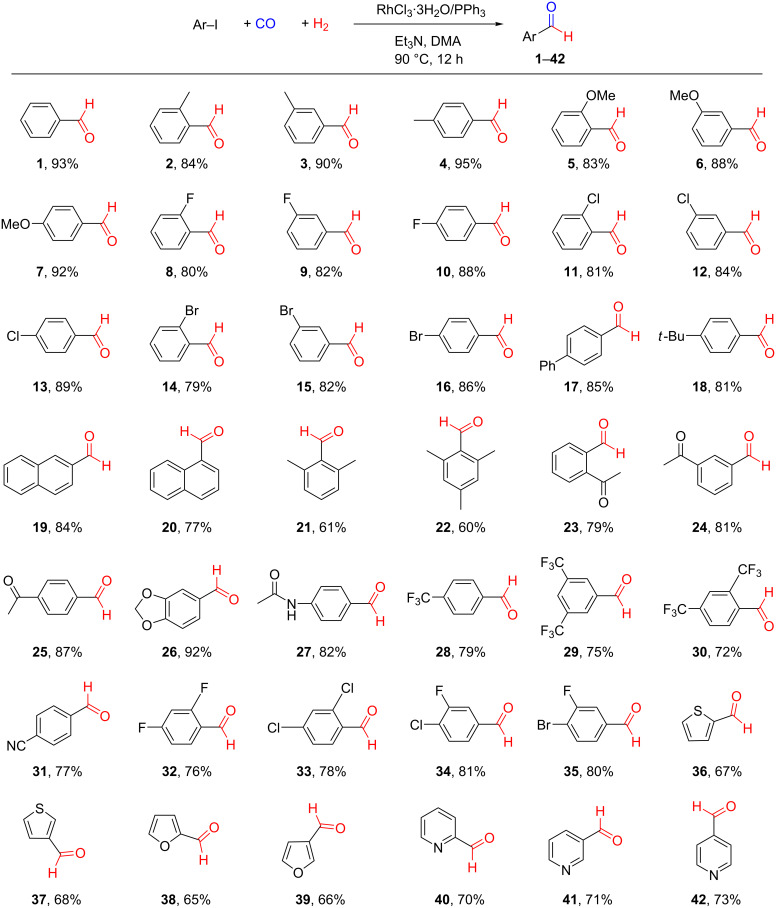
Substrate scope for the Rh-catalyzed reductive carbonylation of aryl iodides using CO and H_2_. Reaction conditions: PhI (1 mmol), RhCl_3_·3H_2_O (2.5 mol %), PPh_3_ (10 mol %), Et_3_N (1.2 mmol), DMA (2 mL), CO (5 bar), H_2_ (5 bar), 90 °C, 12 h. Isolated yields of the products are given and the structures were determined by NMR. Detailed information is given in Supporting Information File 1.

Next, the scope of the reductive carbonylation reaction was explored (shown in Scheme 3), after having identified the optimized conditions for benzaldehyde synthesis from iodobenzene. The results showed that electronic effects had little impact on the reaction. Both, substrates with electron-withdrawing or electron-donating groups afforded similar yields. However, steric effects played an important role in the catalytic process, i.e., the yields were influenced by the substituent group positions following the order of *ortho* < *meta* < *para*. The obtained isolated yields were slightly lower than the NMR yields owing to a loss of products during the workup process. Iodobenzene with no substituent group provided benzaldehyde (**1**) with 93% yield. For iodobenzene derivatives with electron-donating groups (Me, OMe), 83–95% yield of aldehydes **2**–**7** were obtained. As expected, the yields increased in the order of *ortho* < *meta* < *para*-substituted compounds. As for aryl iodides with halide substituents (F, Cl, Br), these were also compatible with the reaction system, affording 79−89% yields of the halogenated aromatic aldehydes **8**–**16**. Iodobenzene derivatives with phenyl or *tert*-butyl groups displaying larger steric hindrance in the *para*-position still provided the corresponding aldehydes **17** and **18** in relatively high yields (85% and 81%, respectively). 2-Iodonaphthalene with smaller steric hindrance performed a little better than 1-iodonaphthalene (**19**, 84% and **20**, 77%). However, starting iodides having methyl substituents in both *ortho*-positions afforded the corresponding aldehydes in much lower yields (**21** 61% and **22** 60%). Also, iodobenzenes with an acetyl group in either *ortho*, *meta*, or *para*-position gave the products in satisfactory yields (**23** 79%, **24** 81% and **25** 87%). 1-Iodo-3,4-methylenedioxybenzene performed well providing aldehyde **26** with 92% yield. Aryl iodide with an acetamido group in the *para*-position gave a medium yield (**27** 82%). Aryl iodides containing one or two trifluoromethyl groups in their structure worked slightly less efficient producing arylaldehydes **28**–**30** with yields of 72–79%. It is worth noticing that a cyano group in the substrate stayed intact in the catalytic process giving aldehyde **31** with 77% yield. Also substrates comprising two halogen substituents performed well offering access to dihalogenated aldehydes **32**–**35** with yields between 76–81%. In addition, heterocyclic iodides were also amenable to the reductive carbonylation reaction and the corresponding aldehydes **36**–**42** were isolated with yields of 65−73%.

### Isotope labeling experiments

**Scheme 4:**
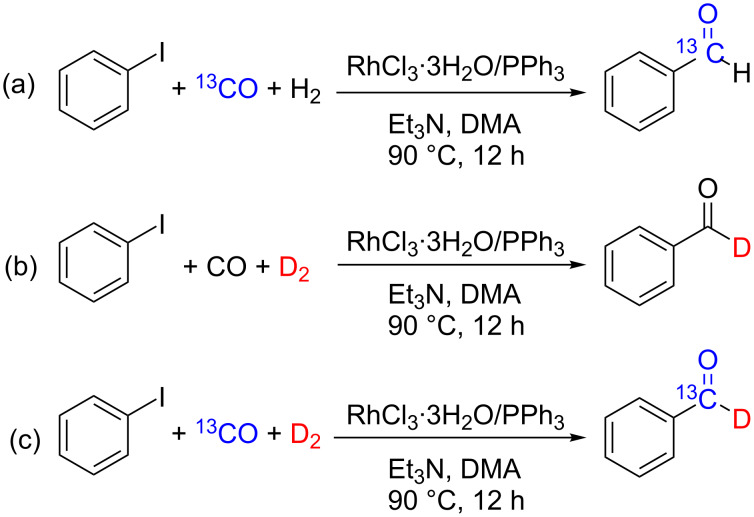
Isotope-labeling experiments.

Isotope labeling experiments were conducted to study the mechanism of the reductive carbonylation of aryl iodide with CO and H_2_ under our optimized conditions, using ^13^CO and D_2_ instead of CO and H_2_, respectively, as the sources of the carbonyl group and hydrogen in the formyl group. C_6_H_5_^13^CHO, C_6_H_5_CDO, and C_6_H_5_^13^CDO could be verified by the molecular ion peaks at *m*/*z* 107, 107, and 108 in the GC–MS spectrum of the reaction solution (for the spectrum, see Supporting Information File 1). This result confirmed that both CO and H_2_ participated in the formation of the formyl group in the product (Scheme 4a–c).

### Reaction mechanism and role of each component

**Scheme 5:**
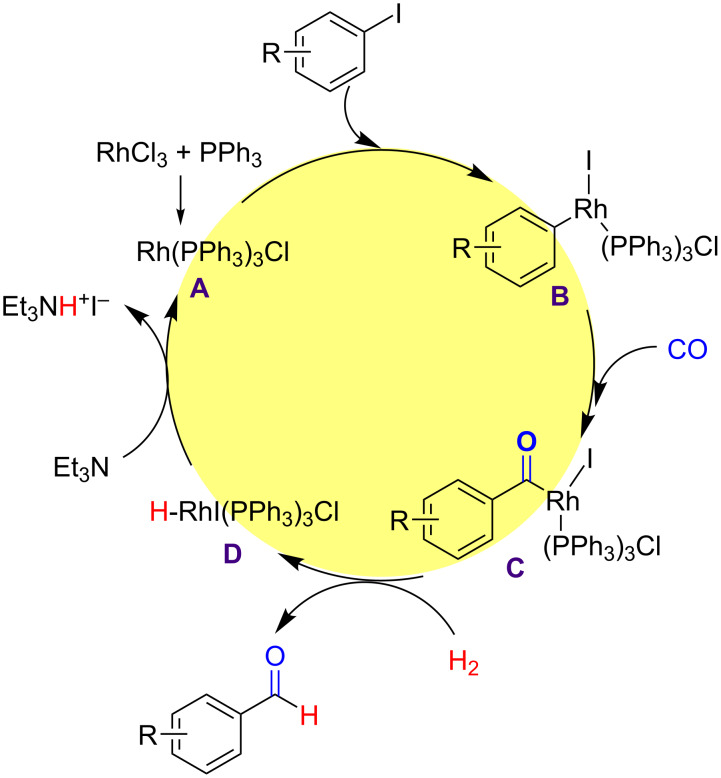
Proposed reaction mechanism for the Rh-catalyzed reductive carbonylation of aryl iodides using CO and H_2_.

Based on the results from the labeling experiments a reaction mechanism for the reductive carbonylation of aryl iodides was proposed, as shown in Scheme 5 [57]. First, RhCl_3_·3H_2_O reacted with PPh_3_ to form Rh(PPh_3_)_3_Cl (**A**), followed by an oxidative addition of Rh(PPh_3_)_3_Cl (**A**) to the aryl iodide, producing the corresponding arylrhodium complex (**B**). Then, the coordination and insertion of CO led to the formation of benzoylrhodium complex (**C**). Next, metathesis with H_2_ afforded the aldehyde product. The base, Et_3_N neutralized the proton in the rhodium hydroiodide complex (**D**) and regenerated the active Rh species [11].

In the catalytic system, CO and H_2_ (or syngas) were used as the carbonyl and hydrogen sources, respectively. The catalyst RhCl_3_·3H_2_O and PPh_3_ reacted via a redox reaction to form Rh(PPh_3_)_3_Cl, which is the active catalytic species able to activate C–I bonds in the aryl iodides for the insertion of CO in the next step. The base Et_3_N neutralized the proton in the intermediate rhodium hydroiodide (**D**) complex and regenerated the active Rh species completing the catalytic cycle.

## Conclusion

An efficient and facile Rh-based catalytic system composed of a commercially available Rh salt, RhCl_3_·3H_2_O, a phosphine ligand PPh_3_, and a base Et_3_N, was evaluated for the synthesis of arylaldehydes via the reductive carbonylation of aryl iodides using CO as carbonyl source and H_2_ as hydrogen source under relatively mild conditions with a broad substrate range. The low gas pressure, cost-effective ligand and low metal dosage significantly improved the practicability of the system for industrial productions. Another advantage of the method includes the use of cheap and abundant syngas (mixtures of CO and H_2_) in the catalytic system as an effective and convenient formyl source at relatively low pressures, which further enhanced the possibility of practical application of the proposed system.

## Experimental

### Materials

Rhodium species ([RhCl(CO)_2_]_2_, RhCl(CO)(PPh_3_)_2_, Rh(acac)(CO)_2_, [RhCl(COD)]_2_, Rh(MeCN)_2_(COD)BF_4_, Rh(COD)BF_4_, Rh(COD)_2_OTf, [Rh(OMe)(COD)]_2_, Rh(OAc)_2_, [Rh(CF_3_COO)]_2_, [Rh(CH_3_(CH_2_)_6_CO_2_)_2_]_2_, Rh(acac)_3_, Rh(ethylene)_2_(acac), [Rh(ethylene)_2_Cl]_2_, Rh(norbornadiene)_2_BF_4_, [Cp*RhCl_2_]_2_, Rh/C, and RhCl_3_·3H_2_O), ligands containing nitrogen or phosphorus (PP_3_, triphos, dpp-BINAP, dpp-OPh, dppb, dppe, P(PhF_5_)_3_, P(4-FPh)_3_, Cydpp, Bipy, DBU, Im, and PPh_3_ (their structures are shown in Table 2)), bases (Et_3_N, 1,4-diaza[2.2.2]bicyclooctane (DABCO), *N*,*N*-diisopropylethylamine (DIPEA), *N*,*N*,*N*',*N*'-tetramethylethylenediamine (TMEDA) and 1,2,2,6,6-pentamethylpiperidine (PMP)), and solvents (*N*,*N*-dimethylacetamide (DMA), 1,3-dimethyl-2-imidazolidinone (DMI), *N*-methyl-2-pyrrolidinone (NMP), tetrahydrofuran (THF), 1,4-dioxane, ethanol, methanol, acetonitrile, and toluene) together with aryl iodides and other reagents were purchased from commercial sources (namely J&K Scientific Ltd. and Innochem Science & Technology Co., Ltd.) and used without further purification. CO and H_2_ with high purity (99.99%) were supplied by Beijing Analytical Instrument Factory.

### Instrumentation

^1^H NMR spectra in solution were recorded in CDCl_3_ using the residual CHCl_3_ as internal reference (7.26 ppm) on a Bruker 400 spectrometer. ^1^H NMR peaks were labeled as singlet (s), doublet (d), triplet (t), and multiplet (m). The coupling constants, *J*, are reported in hertz (Hz). ^13^C NMR spectra in solution were recorded at 101 MHz in CDCl_3_ using the solvent as internal reference (77.0 ppm). GC analysis was performed on Agilent 4890D with a FID detector and a nonpolar capillary column (DB-5) (30 m × 0.25 mm × 0.25 μm). The column oven was temperature-programmed with a 2 min initial hold at 50 °C, followed by heating to 265 °C at a rate of 10 °C /min and kept at 265 °C for 10 min. High purity nitrogen was used as the carrier gas.

### General procedure for reductive carbonylation of aryl iodides with CO and H_2_

All reactions were carried out in an 80 mL Teflon-lined stainless steel reactor equipped with a magnetic stirring bar. Typically, in a glovebox, the aryl iodides (1.0 mmol), RhI_3_ (0.025 mmol), PPh_3_ (0.1 mmol), Et_3_N (1.2 mmol), and DMA (2 mL) were loaded into the reactor. Then, the autoclave was screwed up, charged with CO and H_2_ to a total pressure of 10 bar (1:1) and transferred to an oil bath preheated at 90 °C, which was controlled by a Haake-D3 temperature controller. After completion of the reaction, the reactor was cooled in iced water and the gas carefully vented. The conversion and yield of the aryl iodides and arylaldehydes were determined by GC analysis using dodecane as an internal standard. For yield determination of the other products, the reaction mixture was first analyzed by GC–MS to determine the structures of the aromatic aldehyde products. Then, CH_2_Cl_2_ (5 mL) was added to the reaction mixture, after which deionized water (10 mL) was added to extract the solvent DMA for 5 times. The organic layer was dried over anhydrous Na_2_SO_4_, concentrated by rotary evaporation and finally purified by column chromatography on silica gel using *n*-hexane/ethyl acetate as eluent to obtain the pure products and isolated yields.

### Procedures for the preparations of RhCl(PPh_3_)_3_, RhBr(PPh_3_)_3_ and RhI(PPh_3_)_3_

Preparation of RhCl(PPh_3_)_3_: A round-bottomed flask filled with anhydrous ethanol (60 mL) and triphenylphosphine (10 mmol) was put in an oil bath at 70 °C under a nitrogen atmosphere. Then, hydrated rhodium(III) chloride (1.5 mmol) dissolved in anhydrous ethanol (10 mL) was added to the solution under stirring and the resulting solution was kept at gentle reflux for 2.5 h. Afterwards, the hot mixture was filtered. Finally, the solid was washed with ether and dried under vacuum overnight to afford RhCl(PPh_3_)_3_ as maroon powder. The preparations of RhBr(PPh_3_)_3_ and RhI(PPh_3_)_3_ were similar only with different halide ions.

## Supporting Information

File 1 MS spectra of isotope-labeling experiments and characterization of products.
