# Supplementary material for: Rhodium-catalyzed reductive carbonylation of aryl iodides to arylaldehydes with syngas
Source: Beilstein J Org Chem. 2020 Apr 8;16:645–56. doi: 10.3762/bjoc.16.61 (PMC7155901; doi:10.3762/bjoc.16.61)
Supplement: File 1 — MS spectra of isotope-labeling experiments and characterization of products. [file Beilstein_J_Org_Chem-16-645-s001.pdf]

BEILSTEIN JOURNAL OF ORGANIC CHEMISTRY

Supporting Information

for

Rhodium-catalyzed reductive carbonylation of aryl iodides to arylaldehydes with syngas

Zhenghui Liu, Peng Wang, Zhenzhong Yan, Suqing Chen, Dongkun Yu, Xinhui Zhao and Tiancheng Mu

Beilstein J. Org. Chem. **2020,** *16,* 645–656. doi:10.3762/bjoc.16.61

MS spectra of isotope-labeling experiments and characterization of products

License and Terms: This is a supporting information file under the terms of the Creative Commons Attribution License (http://creativecommons.org/licenses/by/4.0). Please note that the reuse, redistribution and reproduction in particular requires that the authors and source are credited.
The license is subject to the *Beilstein Journal of Organic Chemistry* terms and conditions: (https://www.beilstein-journals.org/bjoc)

Table of Contents

1. MS spectra of ^{13}CO and D_2 in the isotope-labeling experiments……..…..…S2

2. NMR data of the aromatic aldehyde products………………………………...S3

3. NMR spectra of the aromatic aldehyde products…………………………..…….S8

4. References……………………………………………………………………….S50

1. MS spectra of ^{13}CO and D_2 in the isotope-labeling experiments

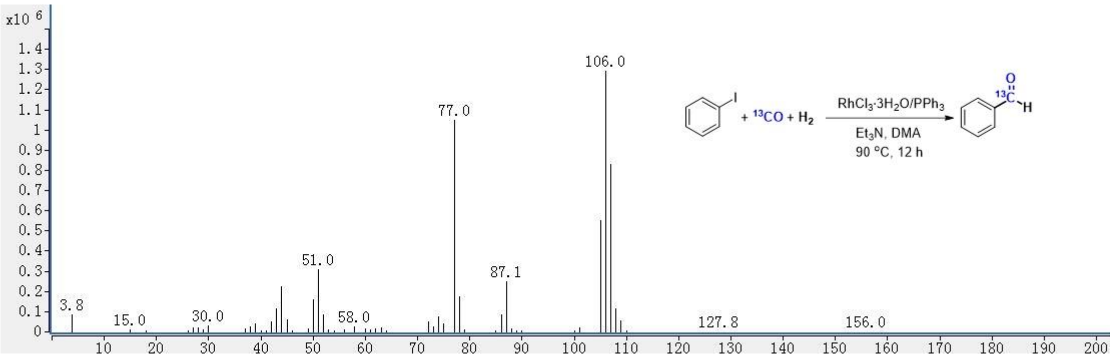

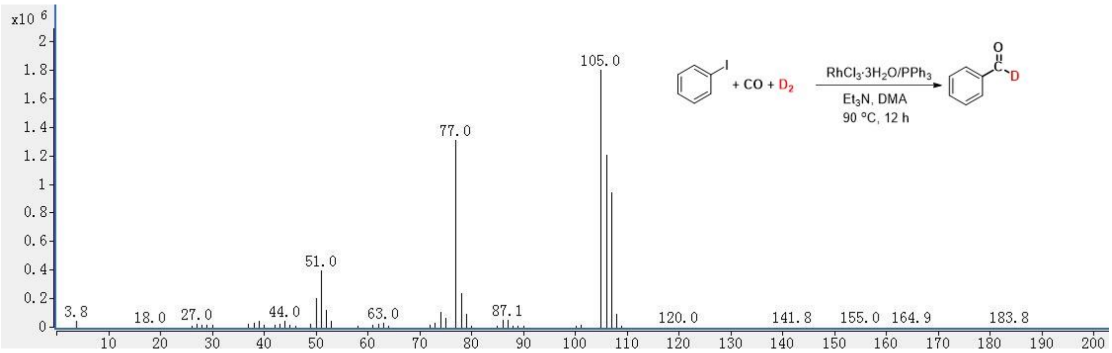

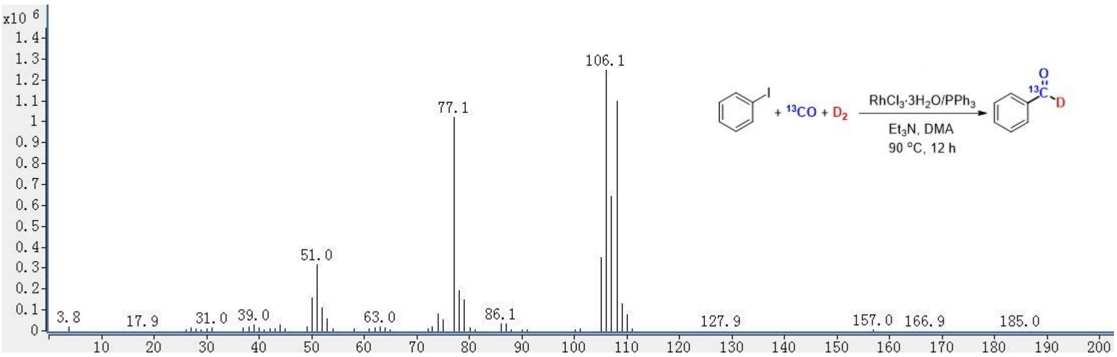

2. NMR data of the aromatic aldehyde products

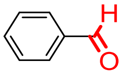
1; Benzaldehyde [1]; ^{1}H NMR (400 MHz, $CDCl_3$) δ 10.02 (s, 1H), 7.94 – 7.81 (m, 2H), 7.69 – 7.40 (m, 3H); ^{13}C NMR (101 MHz, $CDCl_3$) δ 192.35, 136.34, 134.41, 129.68, 128.94.

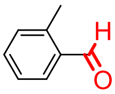
2; 2-Methylbenzaldehyde [1]; ^{1}H NMR (400 MHz, $CDCl_3$) δ 10.26 (s, 1H), 7.79 (dd, *J* = 7.6, 1.5 Hz, 1H), 7.47 (td, *J* = 7.5, 1.6 Hz, 1H), 7.36 (td, *J* = 7.5, 1.2 Hz, 1H), 7.28 – 7.22 (m, 1H), 2.67 (s, 3H); ^{13}C NMR (101 MHz, $CDCl_3$) δ 192.73, 140.53, 134.07, 133.58, 131.98, 131.70, 126.25, 19.52.

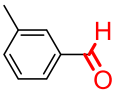
3; 3-Methylbenzaldehyde [2]; ^{1}H NMR (400 MHz, $CDCl_3$) δ 9.97 (s, 1H), 7.74 – 7.61 (m, 2H), 7.47 – 7.33 (m, 2H), 2.42 (s, 3H); ^{13}C NMR (101 MHz, $CDCl_3$) δ 192.53, 138.83, 136.39, 135.21, 129.94, 128.80, 127.14, 21.11.

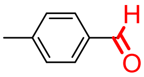
4; 4-Methylbenzaldehyde [1]; ^{1}H NMR (400 MHz, $CDCl_3$) δ 9.96 (s, 1H), 7.77 (d, *J* = 8.1 Hz, 2H), 7.33 (d, *J* = 7.8 Hz, 2H), 2.44 (s, 3H); ^{13}C NMR (101 MHz, $CDCl_3$) δ 191.94, 145.49, 134.13, 129.71, 21.83.

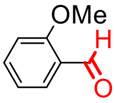
5; 2-Methoxybenzaldehyde [1]; ^{1}H NMR (400 MHz, $CDCl_3$) δ 10.45 (s, 1H), 7.80 (dd, *J* = 7.7, 1.8 Hz, 1H), 7.52 (ddd, *J* = 8.2, 7.4, 1.8 Hz, 1H), 7.08 – 6.90 (m, 2H), 3.90 (s, 3H); ^{13}C NMR (101 MHz, $CDCl_3$) δ 189.62, 161.75, 135.83, 128.38, 124.80, 120.55, 111.58, 55.53.

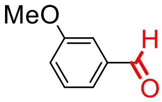
6; 3-Methoxybenzaldehyde [3]; ^{1}H NMR (400 MHz, $CDCl_3$) δ 9.95 (s, 1H), 7.50 – 7.32 (m, 3H), 7.16 (dt, *J* = 5.9, 2.9 Hz, 1H), 3.84 (s, 3H); ^{13}C NMR (101 MHz, $CDCl_3$) δ 192.04, 160.05, 137.70, 129.94, 123.43, 121.40, 111.96, 55.36.

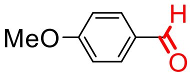
7; 4-Methoxybenzaldehyde [1]; ^{1}H NMR (400 MHz, $CDCl_3$) δ 9.83 (s, 1H), 8.17 – 7.55 (m, 2H), 6.95 (d, *J* = 8.7 Hz, 2H), 3.83 (s, 3H); ^{13}C NMR (101 MHz, $CDCl_3$) δ 190.61, 164.44, 131.79, 129.77, 114.15, 55.41.

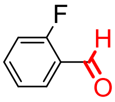
8; 2-Fluorobenzaldehyde [2]; ^{1}H NMR (400 MHz, $CDCl_3$) δ 10.37 (d, *J* = 0.8 Hz, 1H), 7.88 (td, *J* = 7.4, 1.9 Hz, 1H), 7.61 (dddd, *J* = 8.4, 7.3, 5.4, 1.9 Hz, 1H), 7.33 – 7.09 (m, 2H); ^{13}C NMR (101 MHz, $CDCl_3$) δ 187.12, 166.34, 162.92, 136.29, 128.64, 124.58, 116.45.

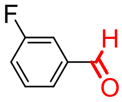
9; 3-Fluorobenzaldehyde [4]; ^{1}H NMR (400 MHz, $CDCl_3$) δ 10.00 (d, *J* = 1.9 Hz, 1H), 7.69 (dt, *J* = 7.5, 1.3 Hz, 1H), 7.61 – 7.48 (m, 2H), 7.39 – 7.26 (m, 1H); ^{13}C NMR (101 MHz, $CDCl_3$) δ 190.82, 164.69, 161.38, 138.36, 130.74, 126.00, 121.52, 115.27.

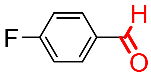

10; 4-Fluorobenzaldehyde [1]; ^{1}H NMR (400 MHz, $CDCl_3$) δ 9.98 (s, 1H), 7.92 (dd, *J* = 8.7, 5.4 Hz, 2H), 7.22 (t, *J* = 8.5 Hz, 2H); ^{13}C NMR (101 MHz, $CDCl_3$) δ 190.45, 168.16, 164.76, 132.91, 132.18, 116.30.

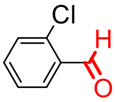

11; 2-Chlorobenzaldehyde [4]; ^{1}H NMR (400 MHz, $CDCl_3$) δ 10.47 (d, *J* = 0.8 Hz, 1H), 7.91 (dd, *J* = 7.7, 1.8 Hz, 1H), 7.53 (ddd, *J* = 8.0, 7.1, 1.8 Hz, 1H), 7.48 – 7.32 (m, 2H); ^{13}C NMR (101 MHz, $CDCl_3$) δ 189.67, 137.81, 135.03, 132.32, 130.49, 129.24, 127.18.

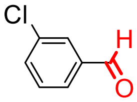

12; 3-Chlorobenzaldehyde [2]; ^{1}H NMR (400 MHz, $CDCl_3$) δ 9.94 (s, 1H), 7.81 (dd, *J* = 2.1, 1.5 Hz, 1H), 7.73 (dt, *J* = 7.5, 1.4 Hz, 1H), 7.56 (ddd, *J* = 8.0, 2.1, 1.2 Hz, 1H), 7.45 (t, *J* = 7.8 Hz, 1H); ^{13}C NMR (101 MHz, $CDCl_3$) δ 190.81, 137.78, 135.40, 134.35, 130.38, 129.22, 127.98.

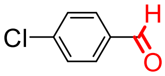

13; 4-Chlorobenzaldehyde [1]; Colorless solid; ^{1}H NMR (400 MHz, $CDCl_3$) δ 9.98 (s, 1H), 7.82 (d, *J* = 8.5 Hz, 2H), 7.51 (d, *J* = 8.4 Hz, 2H); ^{13}C NMR (101 MHz, $CDCl_3$) δ 190.75, 140.81, 134.61, 130.80, 129.34.

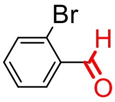

14; 2-Bromobenzaldehyde [4]; ^{1}H NMR (400 MHz, $CDCl_3$) δ 10.29 (d, *J* = 0.7 Hz, 1H), 7.87 – 7.80 (m, 1H), 7.60 – 7.52 (m, 1H), 7.43 – 7.32 (m, 2H); ^{13}C NMR (101 MHz, $CDCl_3$) δ 191.55, 135.14, 133.67, 133.25, 129.63, 127.70, 126.88.

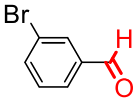

15; 3-Bromobenzaldehyde [5]; ^{1}H NMR (400 MHz, $CDCl_3$) δ 9.92 (s, 1H), 7.95 (d, *J* = 1.8 Hz, 1H), 7.77 (dt, *J* = 7.6, 1.3 Hz, 1H), 7.73 – 7.65 (m, 1H), 7.39 (t, *J* = 7.8 Hz, 1H); ^{13}C NMR (101 MHz, $CDCl_3$) δ 190.52, 137.78, 137.06, 132.05, 130.46, 128.22, 123.15.

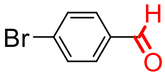

16; 4-Bromobenzaldehyde [3]; ^{1}H NMR (400 MHz, $CDCl_3$) δ 9.96 (s, 1H), 7.73 (d, *J* = 8.5 Hz, 2H), 7.66 (d, *J* = 8.5 Hz, 2H). ^{13}C NMR (101 MHz, $CDCl_3$) δ 190.98, 135.01, 132.37, 130.90, 129.70.

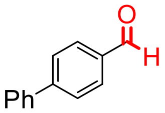

17; 4-Biphenylcarboxaldehyde [1]; ^{1}H NMR (400 MHz, $CDCl_3$) δ 10.06 (s, 1H), 7.96 (d, *J* = 8.3 Hz, 2H), 7.76 (d, *J* = 8.3 Hz, 2H), 7.68 – 7.60 (m, 2H), 7.53 – 7.36 (m, 3H); ^{13}C NMR (101 MHz, $CDCl_3$) δ 191.84, 147.13, 139.65, 135.15, 130.21, 128.96, 128.42, 127.63, 127.31.

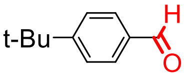

18; 4-*tert*-Butylbenzaldehyde [6]; ^{1}H NMR (400 MHz, $CDCl_3$) δ 9.98 (s, 1H), 7.82 (d, *J* = 8.4 Hz, 2H), 7.55 (d, *J* = 8.4 Hz, 2H), 1.35 (s, 9H); ^{13}C NMR (101 MHz, $CDCl_3$) δ 192.02, 158.41, 134.03, 129.66, 125.95, 35.32, 31.03.

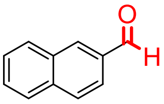

19; 2-Naphthaldehyde [1]; ^{1}H NMR (400 MHz, $CDCl_3$) δ 10.16 (s, 1H), 8.34 (s, 1H), 8.05 – 7.84 (m, 4H), 7.62 (dddd, *J* = 17.6, 8.2, 6.9, 1.4 Hz, 2H); ^{13}C NMR (101 MHz, $CDCl_3$) δ 192.23, 136.42, 134.5, 134.08, 132.61, 129.50, 129.08, 128.05, 127.07, 122.74.

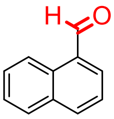

20; 1-Naphthaldehyde [1]; ^{1}H NMR (400 MHz, $CDCl_3$) δ 10.38 (s, 1H), 9.26 (dd, *J* = 8.6, 1.1 Hz, 1H), 8.08 (dt, *J* = 8.2, 1.0 Hz, 1H), 8.01 – 7.83 (m, 2H), 7.79 – 7.44 (m, 3H); ^{13}C NMR (101 MHz, $CDCl_3$) δ 193.45, 136.60, 135.19, 133.60, 131.27, 130.41, 128.97, 128.38, 126.86, 124.77.

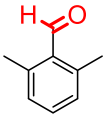

21; 2,6-Dimethylbenzaldehyde [2]; ^{1}H NMR (400 MHz, $CDCl_3$) δ 10.62 (s, 1H), 7.32 (t, *J* = 7.6 Hz, 1H), 7.09 (d, *J* = 7.6 Hz, 2H), 2.61 (s, 6H); ^{13}C NMR (101 MHz, $CDCl_3$) δ 193.39, 140.99, 132.85, 132.30, 129.58, 20.34.

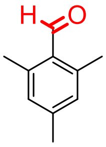

22; Mesitaldehyde [7]; ^{1}H NMR (400 MHz, $CDCl_3$) δ 10.53 (s, 1H), 6.87 (s, 2H), 2.56 (s, 6H), 2.30 (s, 3H); ^{13}C NMR (101 MHz, $CDCl_3$) δ 192.76, 143.66, 141.31, 130.38, 129.82, 21.31, 20.33.

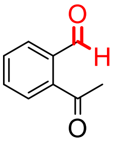

23; 2-Formylacetophenone; ^{1}H NMR (400 MHz, $CDCl_3$) δ 10.22 (s, 1H), 7.89 – 7.83 (m, 1H), 7.68 (ddd, J = 22.9, 6.0, 2.9 Hz, 3H), 2.65 (d, J = 1.7 Hz, 3H); ^{13}C NMR (101 MHz, $CDCl_3$) δ 200.91, 192.12, 140.55, 136.17, 132.95, 131.77, 129.60, 128.40, 28.76.

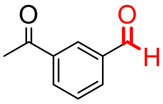

24; 3-Formylacetophenone; ^{1}H NMR (400 MHz, $CDCl_3$) δ 10.04 (s, 1H), 8.38 (t, J = 1.8 Hz, 1H), 8.17 (dt, J = 7.8, 1.5 Hz, 1H), 8.04 (dt, J = 7.6, 1.4 Hz, 1H), 7.61 (t, J = 7.7 Hz, 1H), 2.62 (s, 3H); ^{13}C NMR (101 MHz, $CDCl_3$) δ 196.76, 191.30, 137.68, 136.54, 133.56, 133.43, 129.38, 129.36, 26.53.

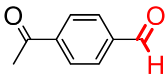

25; 4-Formylacetophenone [6]; ^{1}H NMR (400 MHz, $CDCl_3$) δ 10.10 (s, 1H), 8.10 (d, *J* = 8.3 Hz, 2H), 7.97 (d, *J* = 8.0 Hz, 2H), 2.66 (s, 3H). ^{13}C NMR (101 MHz, $CDCl_3$) δ 197.20, 191.42, 140.97, 138.81, 129.57, 128.59, 26.74.

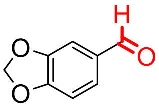

26; Piperonyl aldehyde [1]; ^{1}H NMR (400 MHz, $CDCl_3$) δ 10.30 (s, 1H), 7.16 (dd, *J* = 8.1, 1.7 Hz, 1H), 7.14 (d, *J* = 1.7 Hz, 1H), 6.62 (d, *J* = 8.1 Hz, 1H), 5.99 (s, 2H); ^{13}C NMR (101 MHz, $CDCl_3$) δ 186.81, 152.20, 151.48, 146.02, 128.14, 107.47, 105.06, 103.89.

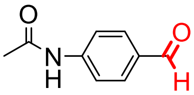
27; 4-Acetamidobenzaldehyde [8]; ^1H NMR (400 MHz, $CDCl_3$) δ 9.91 (s, 1H), 8.00 (s, 1H), 7.89 – 7.79 (m, 2H), 7.71 (d, *J* = 8.4 Hz, 2H), 2.23 (s, 3H); ^{13}C NMR (101 MHz, $CDCl_3$) δ 191.14, 168.86, 143.62, 132.14, 131.14, 119.22, 24.76.

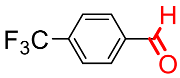
28; 4-(Trifluoromethyl)benzaldehyde [1]; ^1H NMR (400 MHz, $CDCl_3$) δ 10.09 (s, 1H), 8.00 (dt, *J* = 7.9, 0.9 Hz, 2H), 7.80 (d, *J* = 8.1 Hz, 2H); ^{13}C NMR (101 MHz, $CDCl_3$) δ 191.07, 138.63, 135.79, 135.36, 129.89, 126.11, 121.60, 117.99.

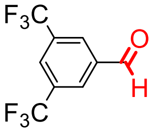
29; 3,5-Bis(trifluoromethyl)benzaldehyde [9]; ^1H NMR (400 MHz, $CDCl_3$) δ 10.13 (s, 1H), 8.34 (d, *J* = 1.8 Hz, 2H), 8.12 (d, *J* = 2.1 Hz, 1H). ^{13}C NMR (101 MHz, $CDCl_3$) δ 189.06, 137.52, 132.78, 129.38, 127.49, 124.10.

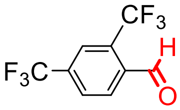
30; 2,4-Bis(trifluoromethyl)benzaldehyde; ^1H NMR (400 MHz, $CDCl_3$) δ 10.04 (d, J = 0.5 Hz, 1H), 8.06 (d, J = 1.5 Hz, 1H), 7.90 (dd, J = 7.5, 0.5 Hz, 1H), 7.72 (dd, J = 7.5, 1.5 Hz, 1H); ^{13}C NMR (101 MHz, $CDCl_3$) δ 189.42, 134.81, 134.80, 134.74, 133.42, 133.17, 129.59, 129.57, 129.56, 129.54, 126.95, 126.92, 126.88, 126.85, 125.22, 125.19, 125.16, 125.13, 124.91, 123.66, 122.76.

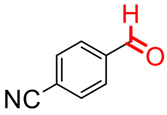
31; 4-Cyanobenzaldehyde [8]; ^1H NMR (400 MHz, $CDCl_3$) δ 10.08 (s, 1H), 7.98 (d, *J* = 8.2 Hz, 2H), 7.84 (d, *J* = 8.2 Hz, 2H); ^{13}C NMR (101 MHz, $CDCl_3$) δ 190.56, 138.65, 132.82, 129.80, 117.57.

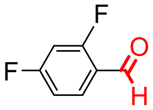
32; 2,4-Difluorobenzaldehyde; ^1H NMR (400 MHz, $CDCl_3$) δ 10.28 (s, 1H), 7.91 (td, J = 8.3, 6.5 Hz, 1H), 7.02 – 6.97 (m, 1H), 6.91 (ddd, J = 10.8, 8.6, 2.4 Hz, 1H); ^{13}C NMR (101 MHz, $CDCl_3$) δ 185.64, 185.59, 168.04, 167.95, 166.55, 166.45, 165.98, 165.88, 164.47, 164.37, 130.74, 130.72, 130.66, 130.63, 121.12, 121.10, 121.06, 112.73, 112.70, 112.55, 112.52, 104.96, 104.76, 104.56.

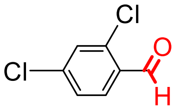
33; 2,4-Dichlorobenzaldehyde; ^1H NMR (400 MHz, $CDCl_3$) δ 10.23 (d, J = 0.5 Hz, 1H), 7.74 (dd, J = 7.5, 0.5 Hz, 1H), 7.50 (d, J = 1.5 Hz, 1H), 7.41 (dd, J = 7.5, 1.5 Hz, 1H); ^{13}C NMR (101 MHz, $CDCl_3$) δ 188.19, 135.58, 134.02, 131.52, 130.26, 130.11, 128.63.

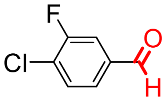
34; 4-Chloro-3-fluorobenzaldehyde; ^1H NMR (400 MHz, $CDCl_3$) δ 9.92 (t, J = 0.5 Hz, 1H), 7.65 (dddd, J = 9.7, 8.0, 1.5, 0.5 Hz, 2H), 7.46 (dd, J = 7.4, 5.0 Hz, 1H); ^{13}C NMR (101 MHz, $CDCl_3$) δ 190.35, 190.33, 159.21, 157.20, 134.98, 134.91, 129.87, 129.85, 127.87, 127.81, 126.08, 125.92, 116.24, 116.08.

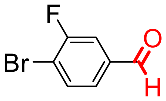
35; 4-Bromo-3-fluorobenzaldehyde; ^{1}H NMR (400 MHz, $CDCl_3$) δ 9.92 (d, *J* = 1.8 Hz, 1H), 7.72 (t, *J* = 7.2 Hz, 1H), 7.60 – 7.45 (m, 2H); ^{13}C NMR (101 MHz, $CDCl_3$) δ 189.77, 160.71, 158.21, 137.20, 134.44, 126.39, 116.71, 116.50, 116.15, 115.92.

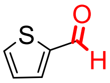
36; 2-Thiophenecarbaldehyde [1]; ^{1}H NMR (400 MHz, $CDCl_3$) δ 9.95 (d, *J* = 1.2 Hz, 1H), 7.97 – 7.54 (m, 2H), 7.22 (dd, *J* = 4.9, 3.8 Hz, 1H); ^{13}C NMR (101 MHz, $CDCl_3$) δ 182.93, 143.96, 136.27, 135.06, 128.26.

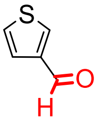
37; 3-Thiophene carboxaldehyde [1]; ^{1}H NMR (400 MHz, $CDCl_3$) δ 9.90 (d, *J* = 0.8 Hz, 1H), 8.11 (dd, *J* = 2.9, 1.2 Hz, 1H), 7.51 (dd, *J* = 5.1, 1.2 Hz, 1H), 7.35 (ddd, *J* = 5.1, 2.9, 0.8 Hz, 1H); ^{13}C NMR (101 MHz, $CDCl_3$) δ 184.81, 142.81, 136.65, 127.25, 125.11.

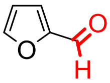
38; 2-Furaldehyde; ^{1}H NMR (400 MHz, $CDCl_3$) δ 9.63 (s, 1H), 7.67 (s, 1H), 7.23 (d, J = 4.8 Hz, 1H), 6.58 (dd, J = 4.4, 1.9 Hz, 1H); ^{13}C NMR (101 MHz, $CDCl_3$) δ 177.70, 152.80, 147.96, 112.45.

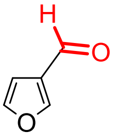
39; 3-Furaldehyde [10]; ^{1}H NMR (400 MHz, $CDCl_3$) δ 9.89 (s, 1H), 8.06 (s, 1H), 7.44 (s, 1H), 6.72 (s, 1H); ^{13}C NMR (101 MHz, $CDCl_3$) δ 184.27, 151.28, 144.77, 128.50, 106.70.

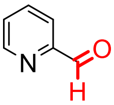
40; 2-Pyridinecarboxaldehyde [3]; ^{1}H NMR (400 MHz, $CDCl_3$) δ 10.09 (s, 1H), 8.81 (dt, *J* = 4.8, 1.3 Hz, 1H), 7.98 (dt, *J* = 7.8, 1.2 Hz, 1H), 7.91 (td, *J* = 7.6, 1.6 Hz, 1H), 7.57 (ddd, *J* = 7.4, 4.8, 1.4 Hz, 1H); ^{13}C NMR (101 MHz, $CDCl_3$) δ 193.20, 152.60, 150.02, 136.89, 127.70, 121.51.

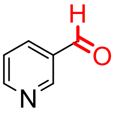
41; 3-Pyridinecarboxaldehyde [11]; ^{1}H NMR (400 MHz, $CDCl_3$) δ 10.04 (s, 1H), 8.99 (d, *J* = 2.1 Hz, 1H), 8.75 (dd, *J* = 4.9, 1.8 Hz, 1H), 8.08 (dt, *J* = 7.9, 2.0 Hz, 1H), 7.41 (dd, *J* = 7.9, 4.8 Hz, 1H); ^{13}C NMR (101 MHz, $CDCl_3$) δ 190.60, 154.55, 151.85, 135.58, 131.19, 123.87.

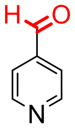
42; 4-Pyridinecarboxaldehyde [12]; ^{1}H NMR (400 MHz, $CDCl_3$) δ 9.98 (s, 1H), 8.77 (d, *J* = 6.0 Hz, 2H), 7.60 (d, *J* = 6.0 Hz, 2H); ^{13}C NMR (101 MHz, $CDCl_3$) δ 191.29, 150.97, 141.15, 121.85.

3. NMR spectra of the aromatic aldehyde products

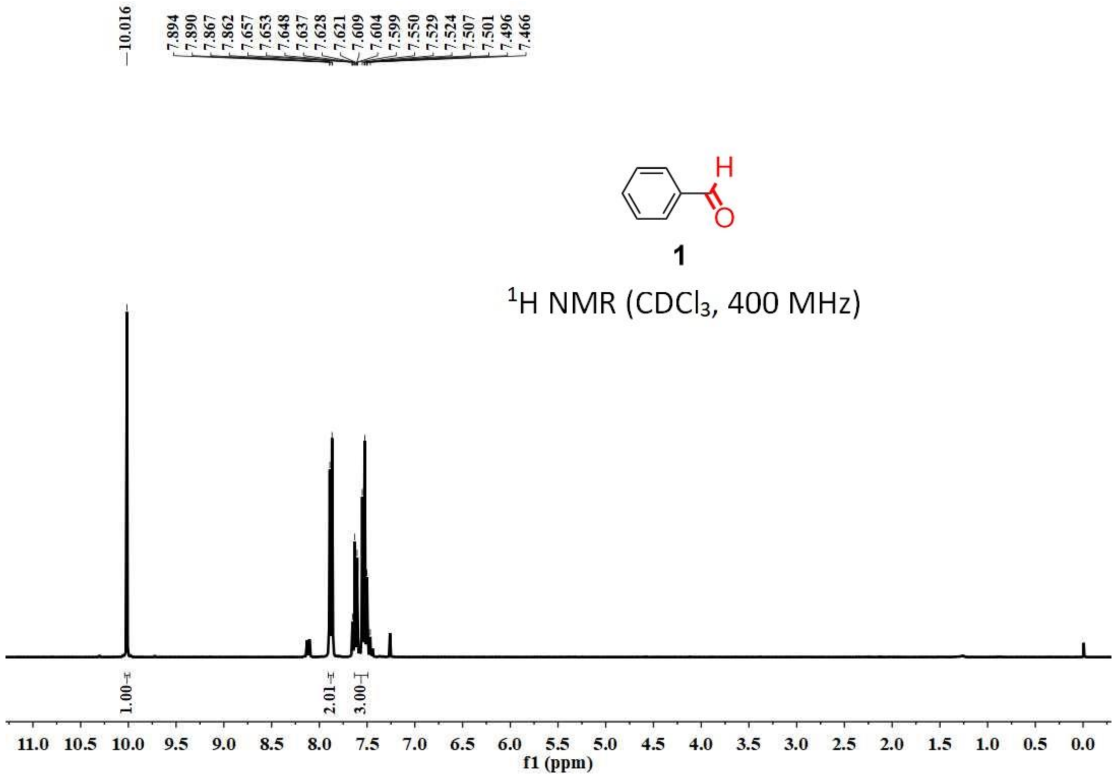

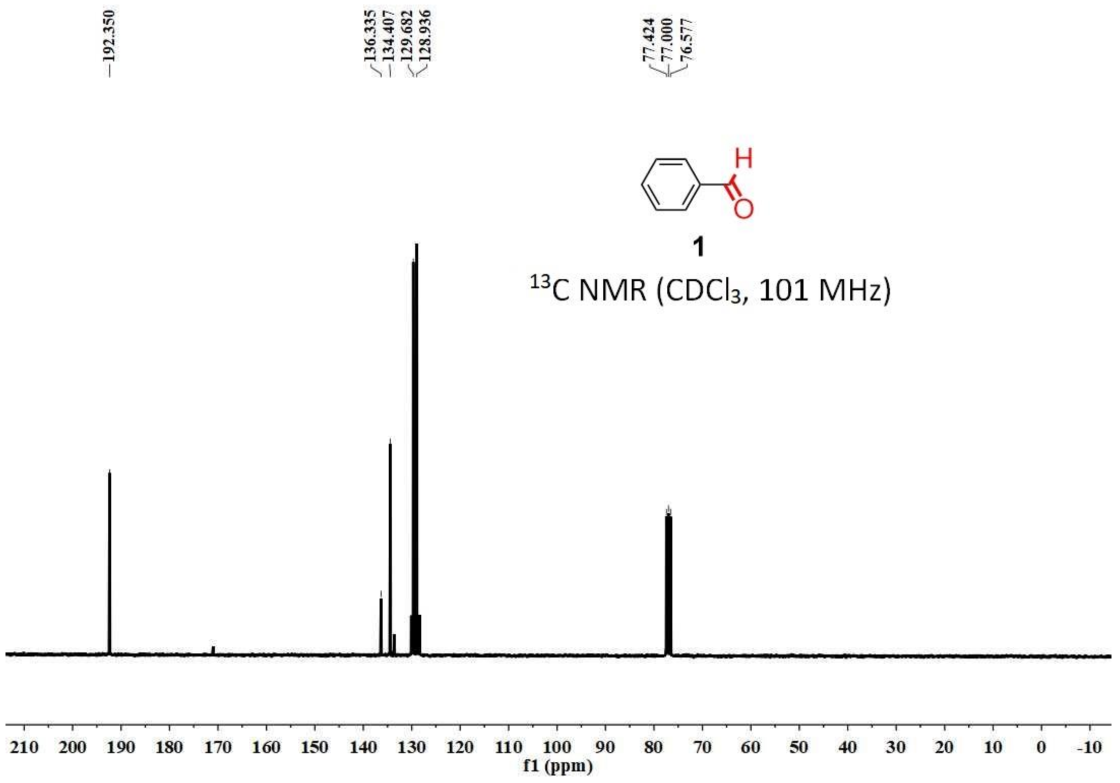

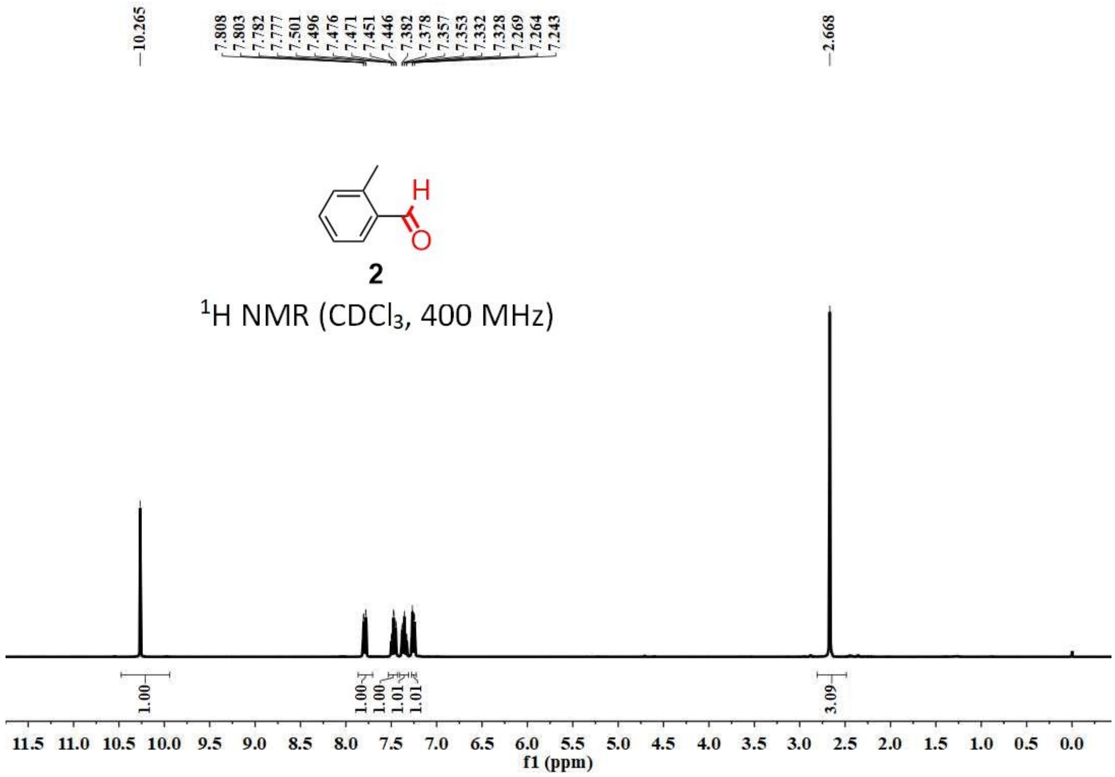

2

^{1}H NMR ($CDCl_3$, 400 MHz)

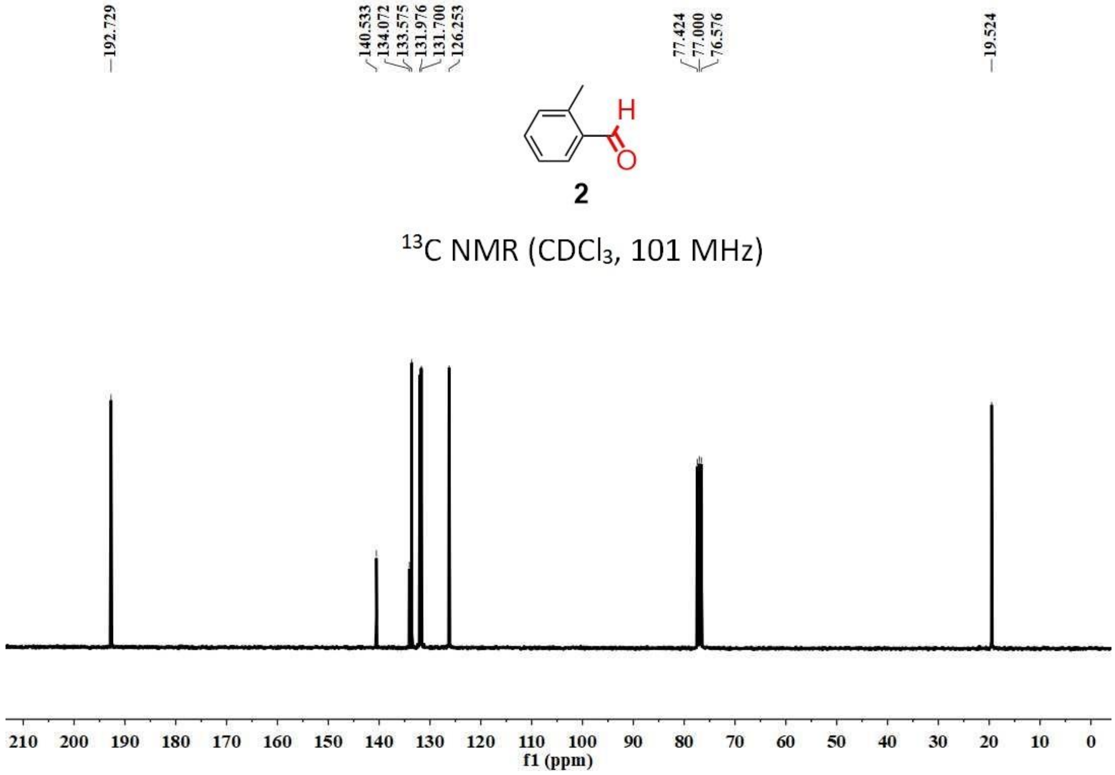

2

^{13}C NMR ($CDCl_3$, 101 MHz)

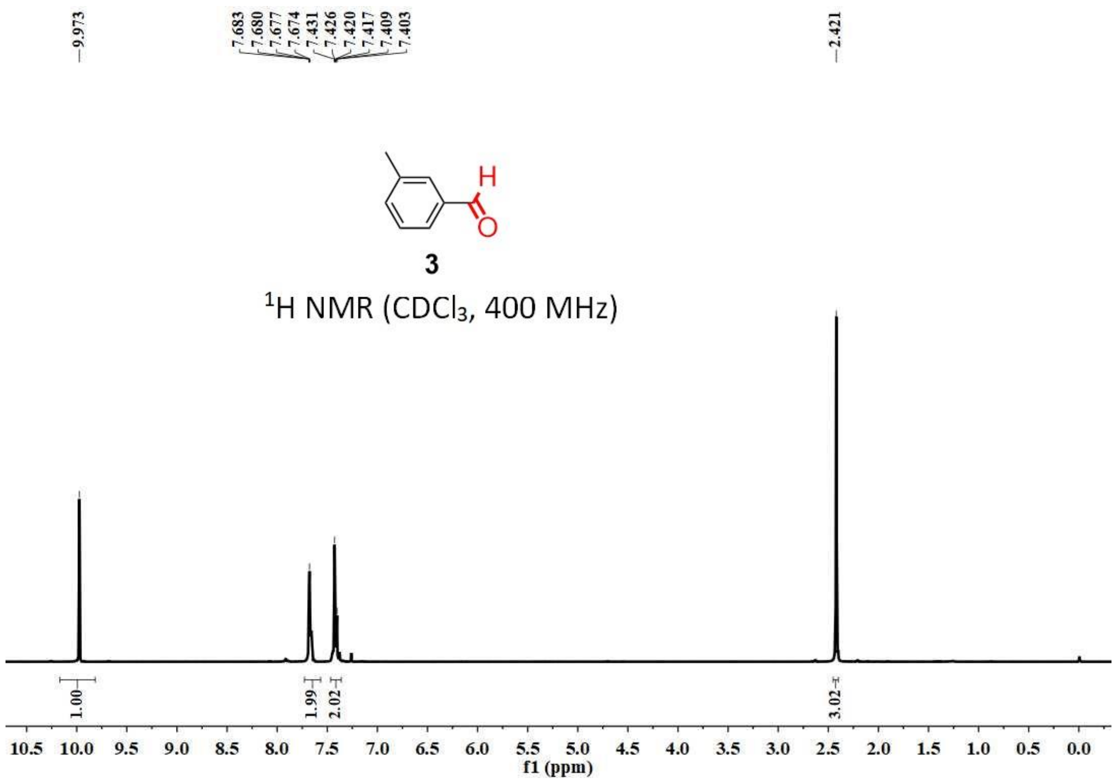

3

^{1}H NMR ($CDCl_3$, 400 MHz)

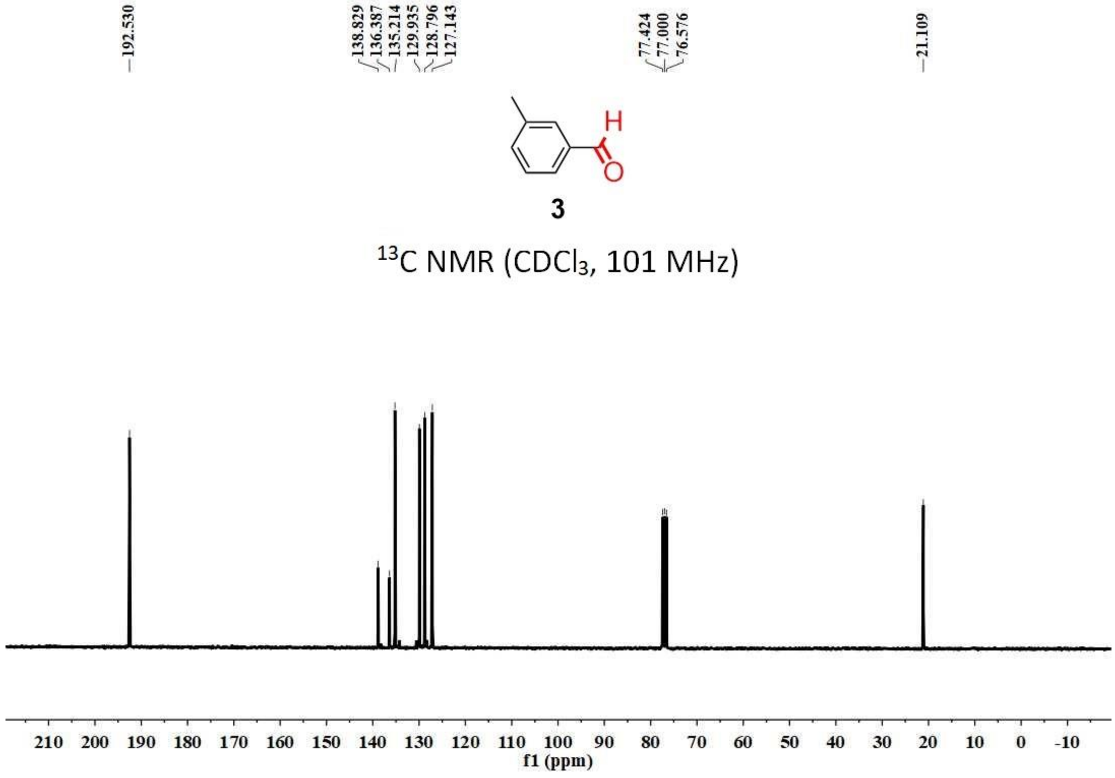

3

^{13}C NMR ($CDCl_3$, 101 MHz)

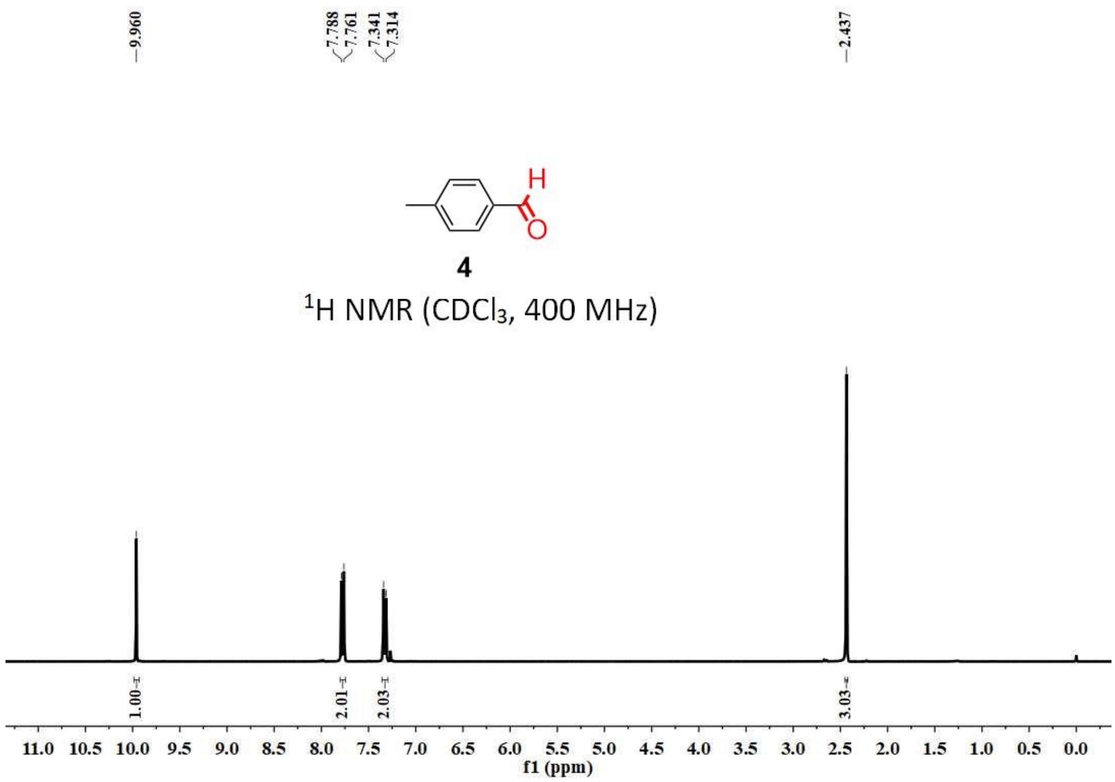
9.960
7.788
7.761
7.341
7.314
2.437
4
1H NMR (CDCl3, 400 MHz)
1.00
2.01
2.03
3.03
11.0 10.5 10.0 9.5 9.0 8.5 8.0 7.5 7.0 6.5 6.0 5.5 5.0 4.5 4.0 3.5 3.0 2.5 2.0 1.5 1.0 0.5 0.0
f1 (ppm)

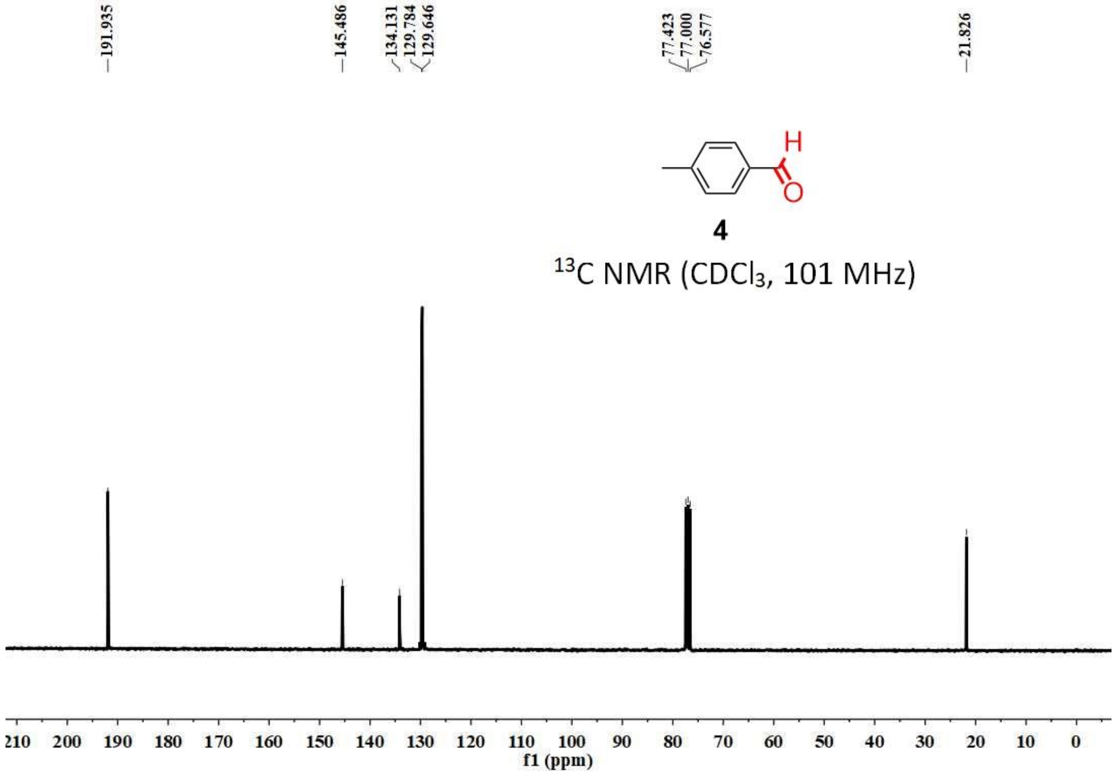
191.935
145.486
134.131
129.784
129.646
77.423
77.000
76.577
21.826
4
13C NMR (CDCl3, 101 MHz)
210 200 190 180 170 160 150 140 130 120 110 100 90 80 70 60 50 40 30 20 10 0
f1 (ppm)

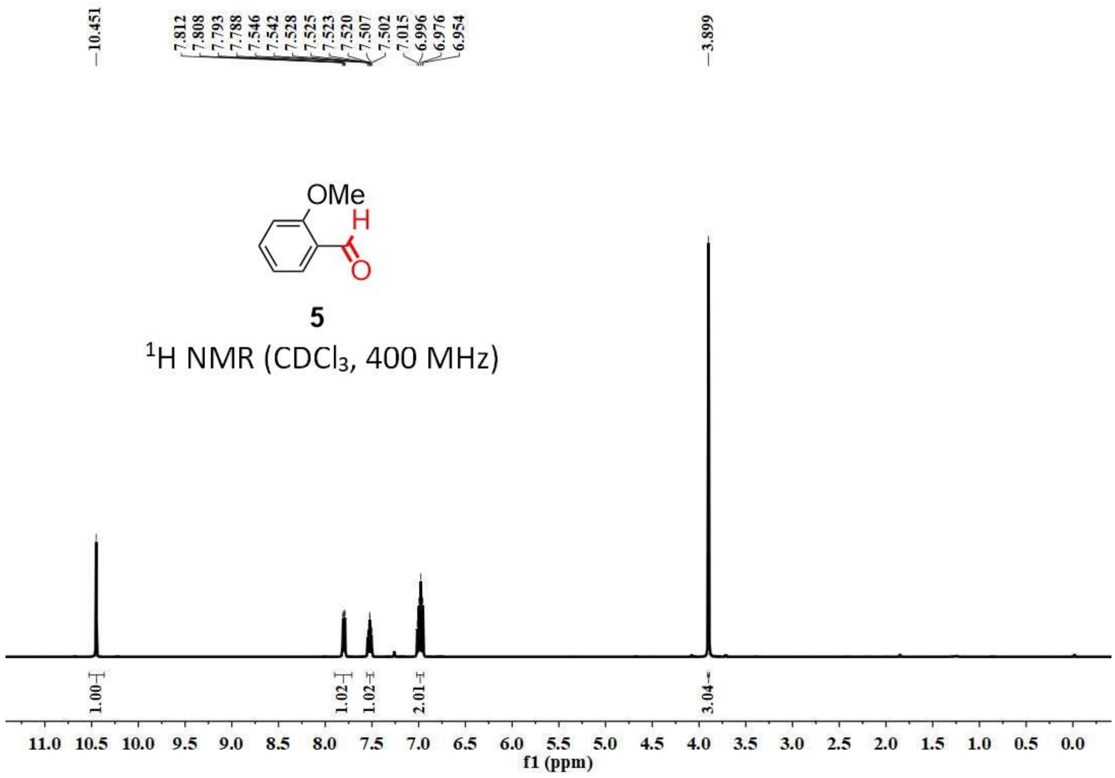
10.451
7.812
7.808
7.793
7.788
7.546
7.542
7.528
7.525
7.523
7.520
7.507
7.502
7.015
6.996
6.976
6.954
3.899
OMe
H
O
5
^{1}H NMR ($CDCl_3$, 400 MHz)
1.00
1.02
1.02
2.01
3.04
11.0 10.5 10.0 9.5 9.0 8.5 8.0 7.5 7.0 6.5 6.0 5.5 5.0 4.5 4.0 3.5 3.0 2.5 2.0 1.5 1.0 0.5 0.0
f1 (ppm)

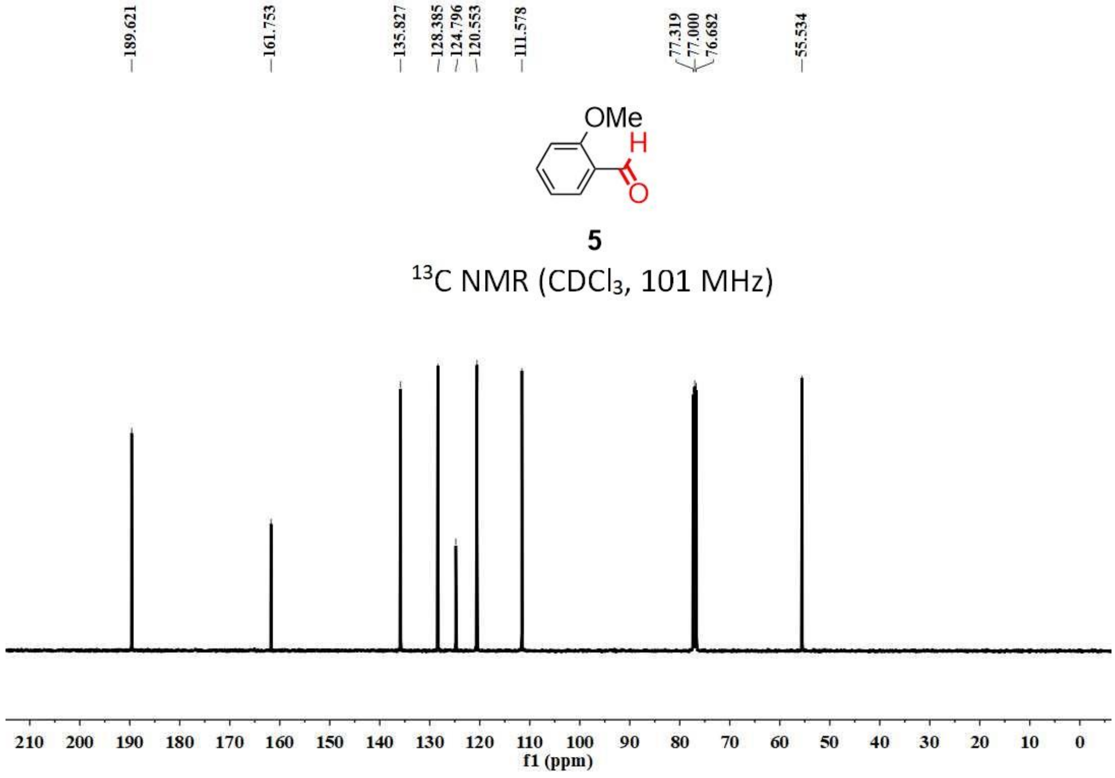
189.621
161.753
135.827
128.385
124.796
120.553
111.578
77.319
77.000
76.682
55.534
OMe
H
O
5
^{13}C NMR ($CDCl_3$, 101 MHz)
210 200 190 180 170 160 150 140 130 120 110 100 90 80 70 60 50 40 30 20 10 0
f1 (ppm)

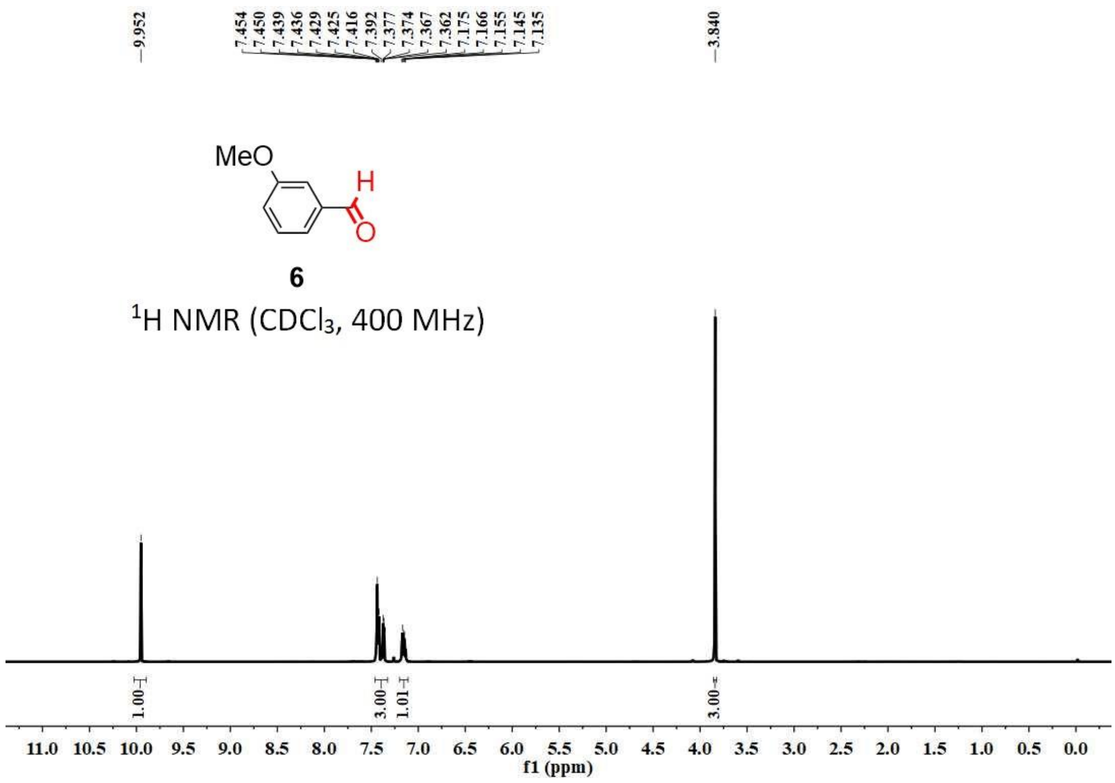

9.952
7.454
7.450
7.439
7.436
7.429
7.425
7.416
7.392
7.377
7.374
7.367
7.362
7.175
7.166
7.155
7.145
7.135
3.840
MeO
H
O
6
¹H NMR (CDCl₃, 400 MHz)
1.00
3.00
1.01
3.00
11.0 10.5 10.0 9.5 9.0 8.5 8.0 7.5 7.0 6.5 6.0 5.5 5.0 4.5 4.0 3.5 3.0 2.5 2.0 1.5 1.0 0.5 0.0
f1 (ppm)

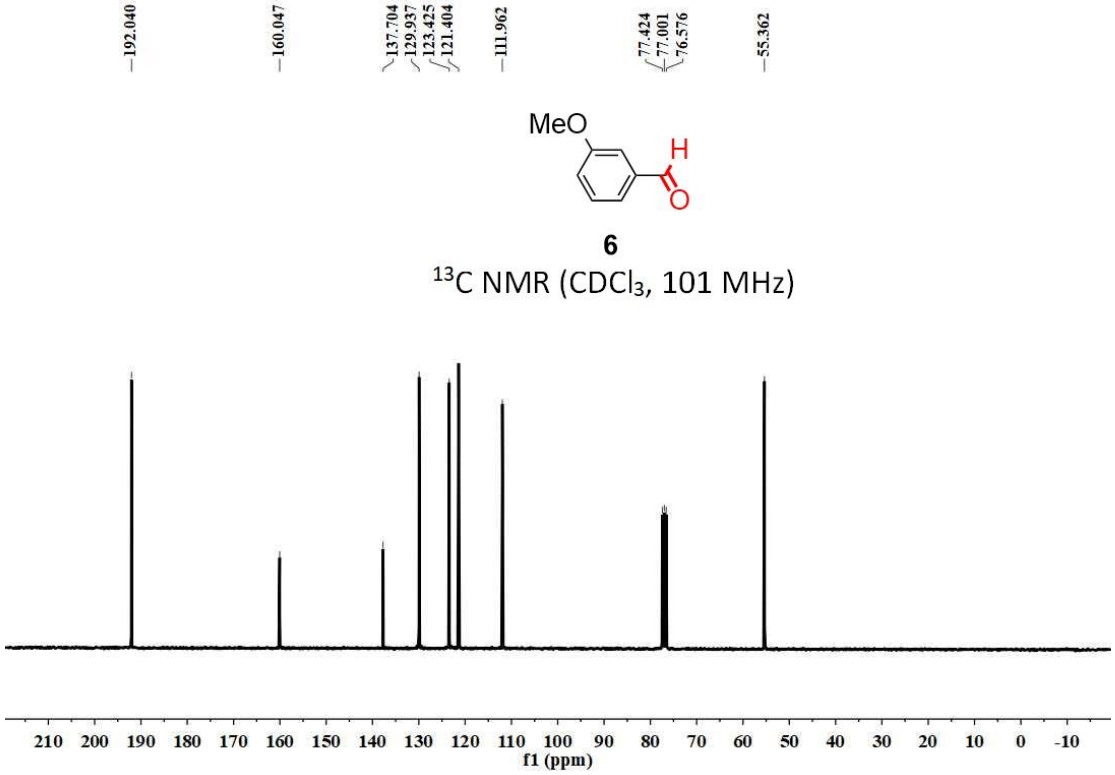

192.040
160.047
137.704
129.937
123.425
121.404
111.962
77.424
77.001
76.576
55.362
MeO
H
O
6
¹³C NMR (CDCl₃, 101 MHz)
210 200 190 180 170 160 150 140 130 120 110 100 90 80 70 60 50 40 30 20 10 0 -10
f1 (ppm)

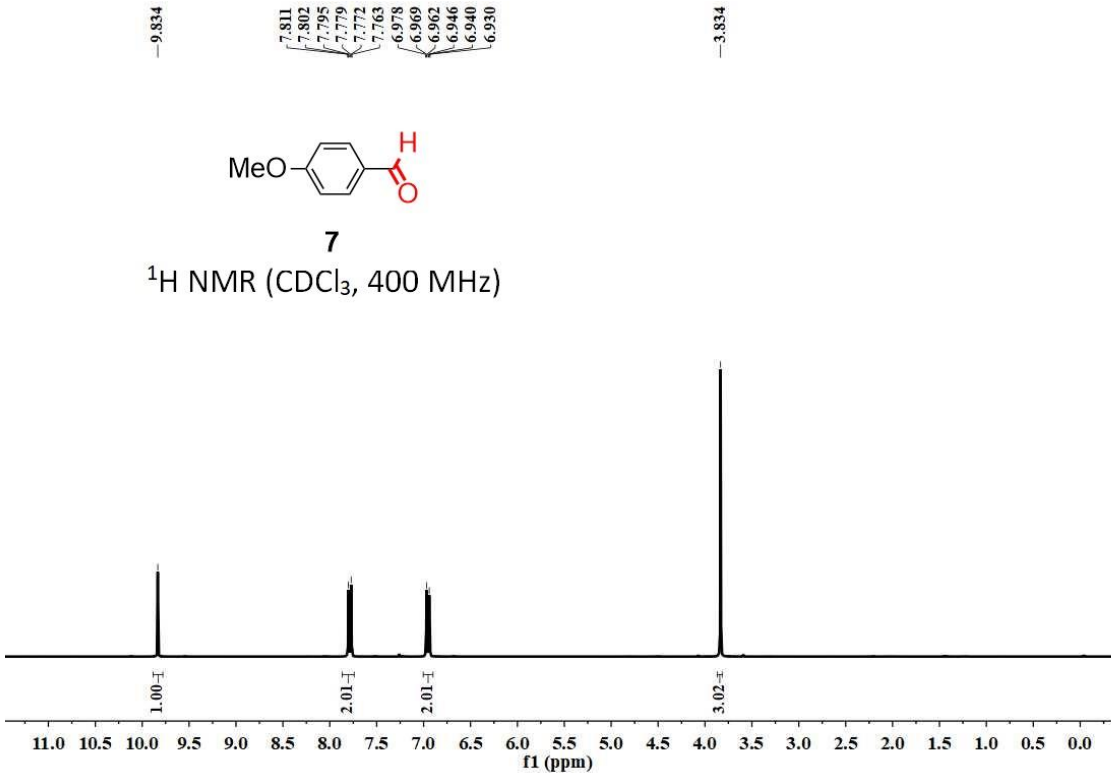
9.834
7.811
7.802
7.795
7.779
7.772
7.763
6.978
6.969
6.962
6.946
6.940
6.930
3.834
MeO
H
O
7
1H NMR (CDCl3, 400 MHz)
1.00
2.01
2.01
3.02
11.0 10.5 10.0 9.5 9.0 8.5 8.0 7.5 7.0 6.5 6.0 5.5 5.0 4.5 4.0 3.5 3.0 2.5 2.0 1.5 1.0 0.5 0.0
f1 (ppm)

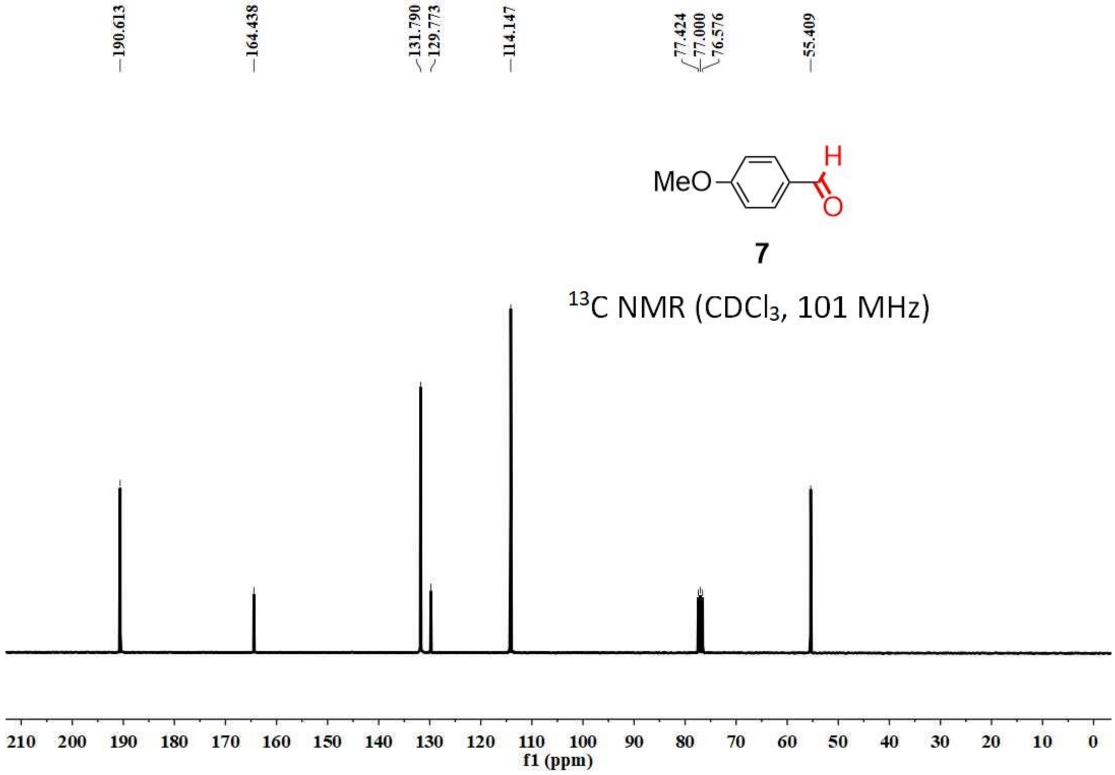
190.613
164.438
131.790
129.773
114.147
77.424
77.000
76.576
55.409
MeO
H
O
7
13C NMR (CDCl3, 101 MHz)
210 200 190 180 170 160 150 140 130 120 110 100 90 80 70 60 50 40 30 20 10 0
f1 (ppm)

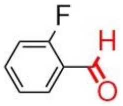

8

^{1}H NMR ($CDCl_3$, 400 MHz)

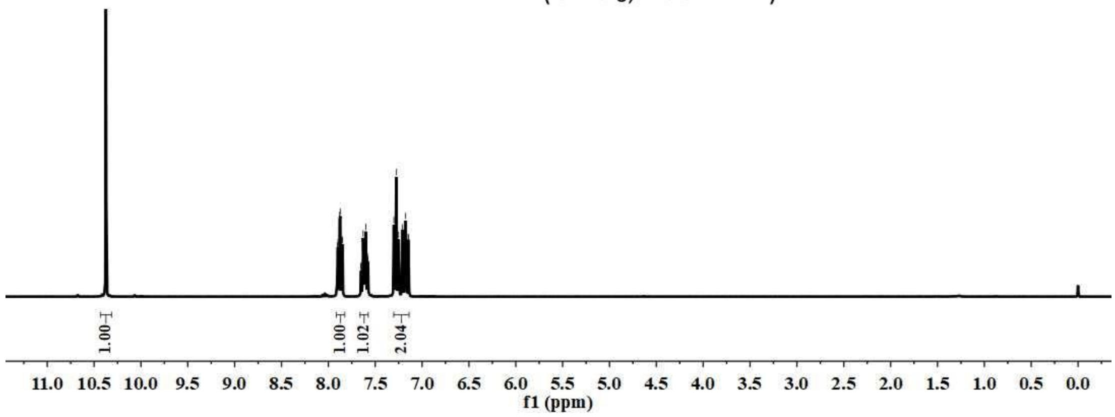

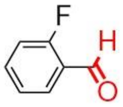

8

^{13}C NMR ($CDCl_3$, 101 MHz)

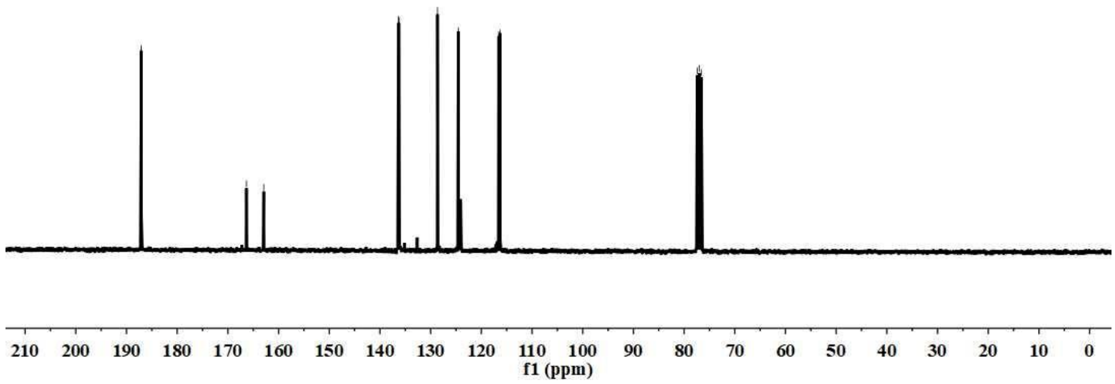

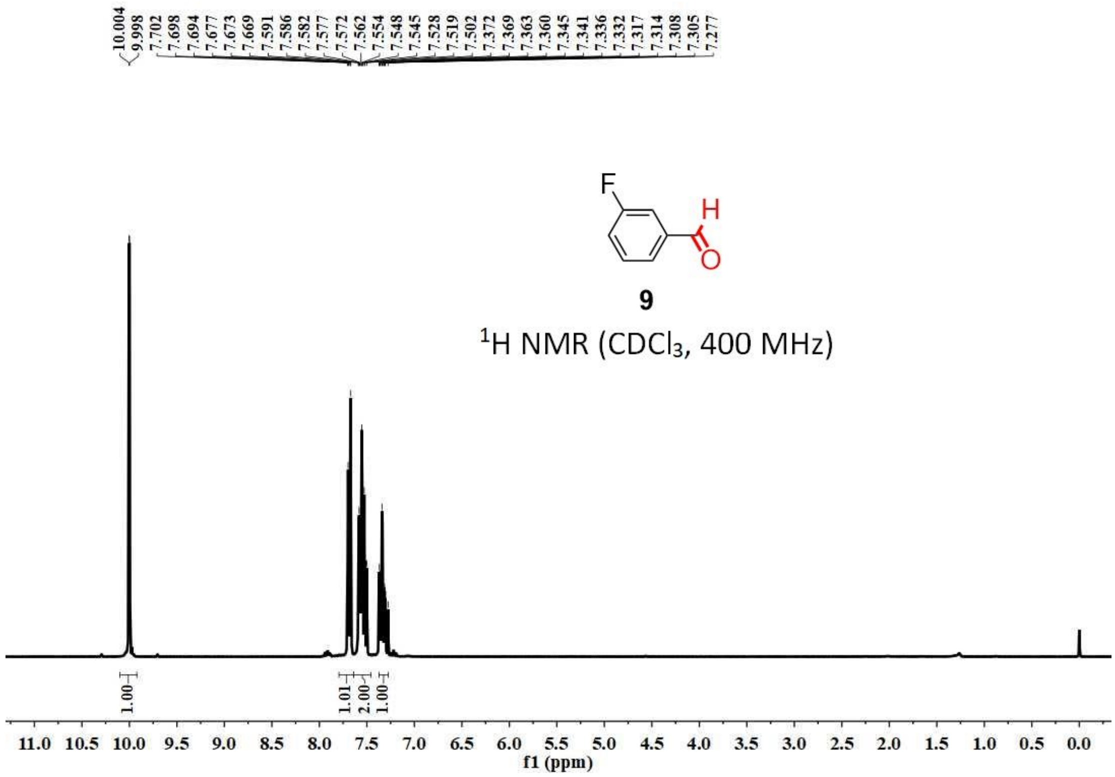
10.004
9.998
7.702
7.698
7.694
7.677
7.673
7.669
7.591
7.586
7.582
7.577
7.572
7.562
7.554
7.548
7.545
7.528
7.519
7.502
7.372
7.369
7.363
7.360
7.345
7.341
7.336
7.332
7.317
7.314
7.308
7.305
7.277
F
H
O
9
1H NMR (CDCl3, 400 MHz)
1.00
1.01
2.00
1.00
11.0 10.5 10.0 9.5 9.0 8.5 8.0 7.5 7.0 6.5 6.0 5.5 5.0 4.5 4.0 3.5 3.0 2.5 2.0 1.5 1.0 0.5 0.0
f1 (ppm)

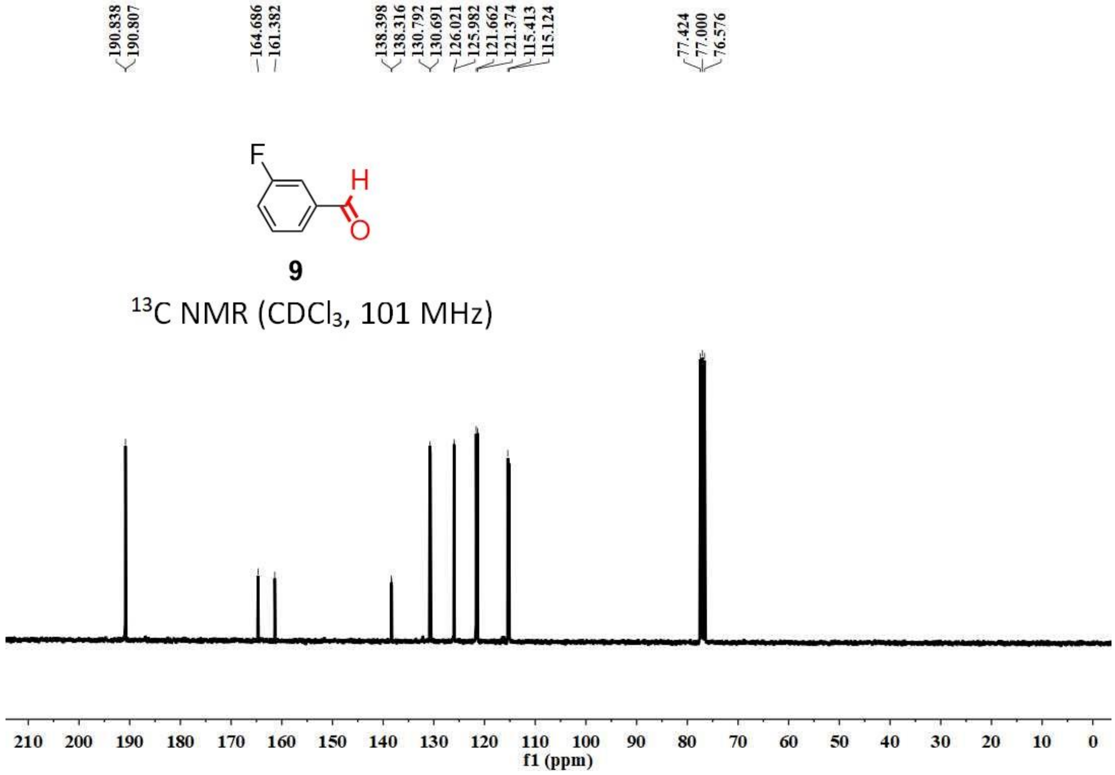
190.838
190.807
164.686
161.382
138.398
138.316
130.792
130.691
126.021
125.982
121.662
121.374
115.413
115.124
77.424
77.000
76.576
F
H
O
9
13C NMR (CDCl3, 101 MHz)
210 200 190 180 170 160 150 140 130 120 110 100 90 80 70 60 50 40 30 20 10 0
f1 (ppm)

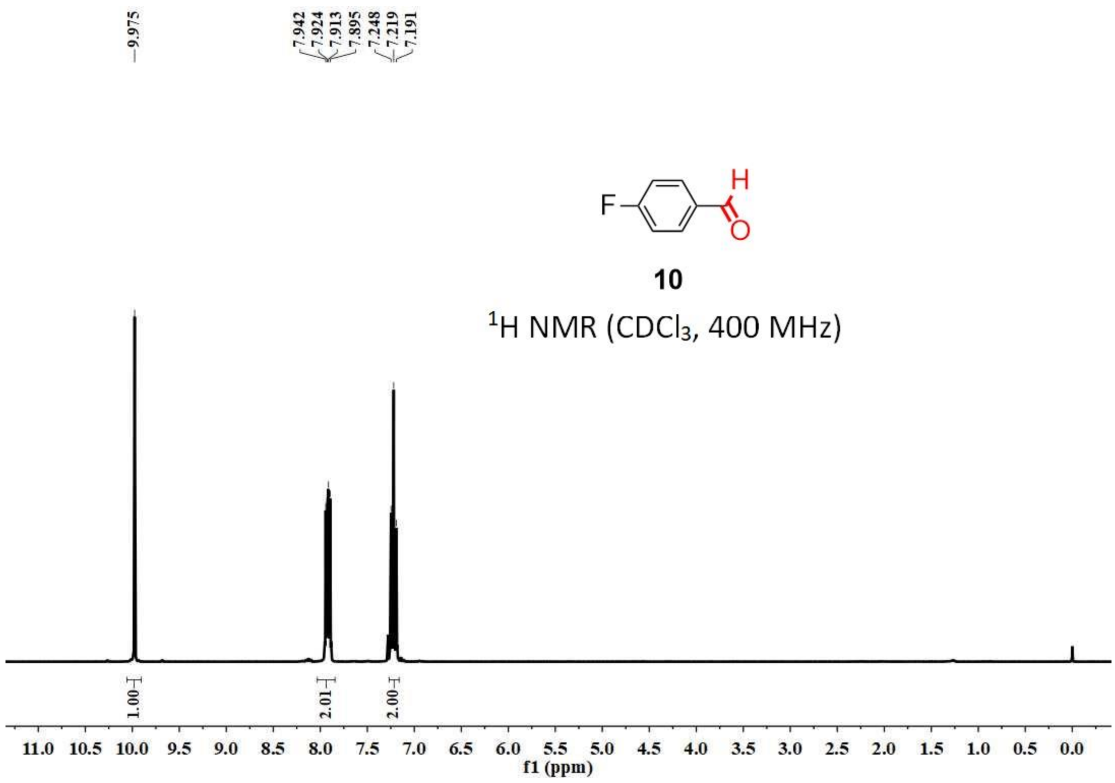

10

^{1}H NMR ($CDCl_3$, 400 MHz)

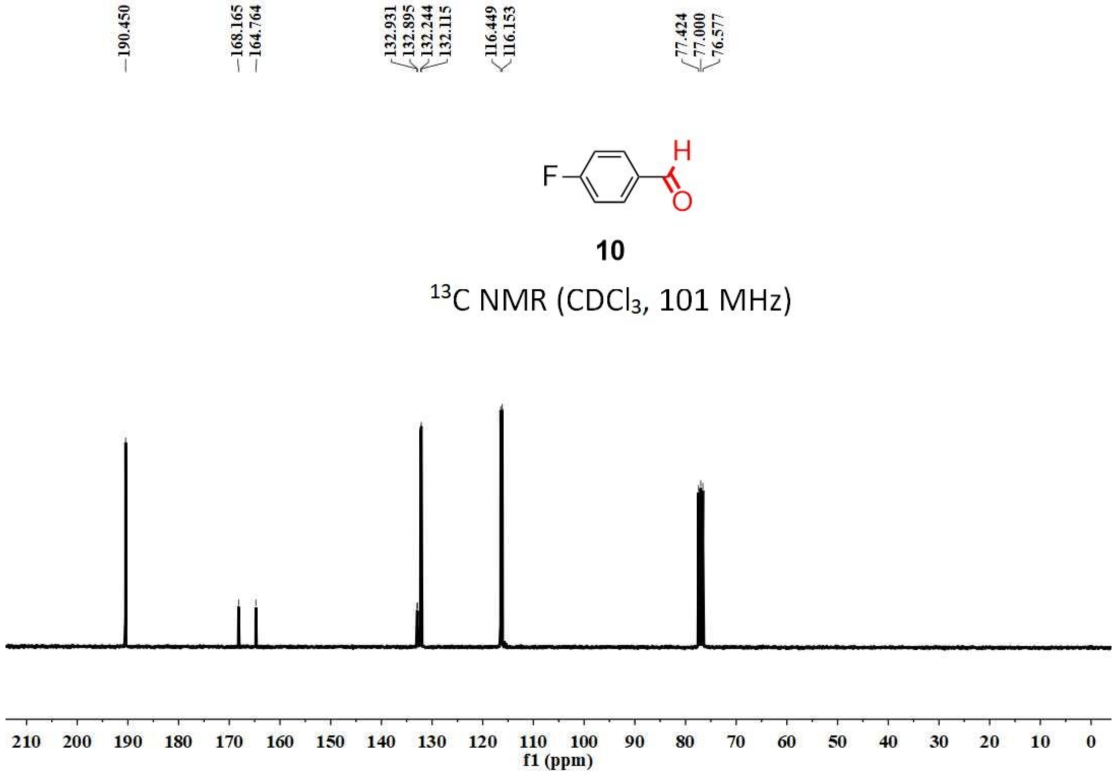

10

^{13}C NMR ($CDCl_3$, 101 MHz)

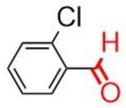

11

^{1}H NMR ($CDCl_3$, 400 MHz)

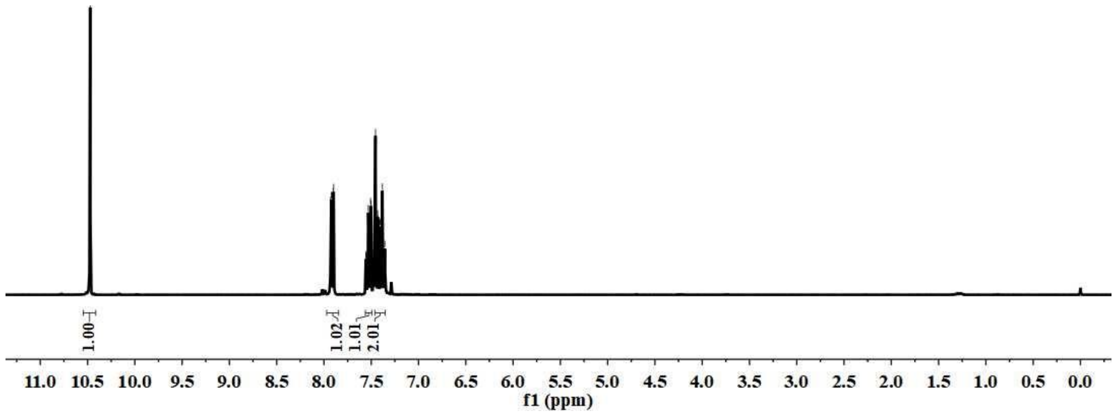

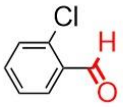

11

^{13}C NMR ($CDCl_3$, 101 MHz)

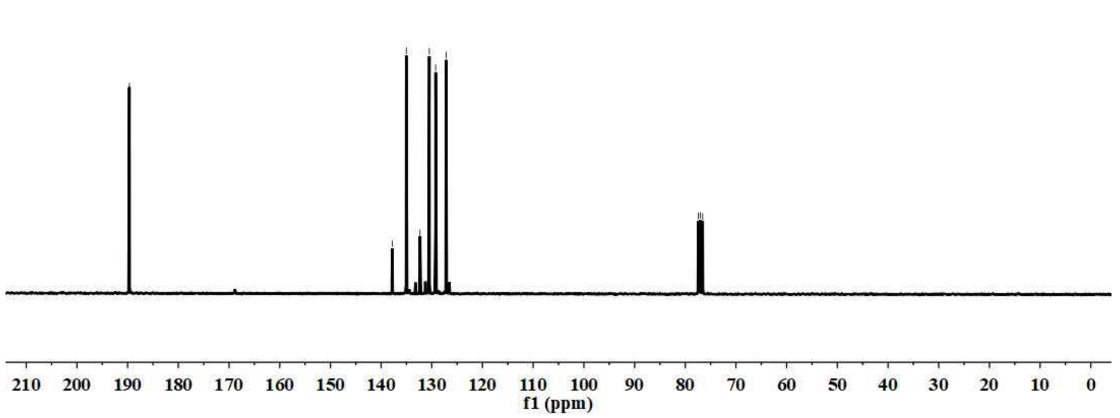

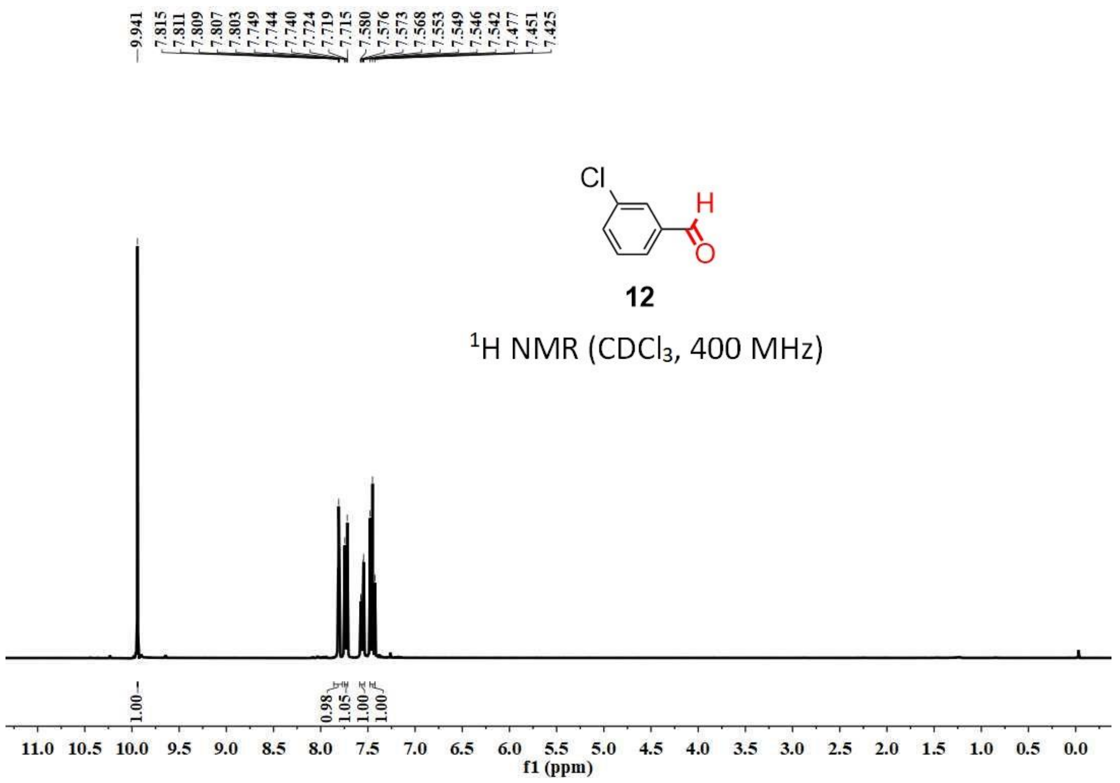

12

^{1}H NMR (CDCl$_3$, 400 MHz)

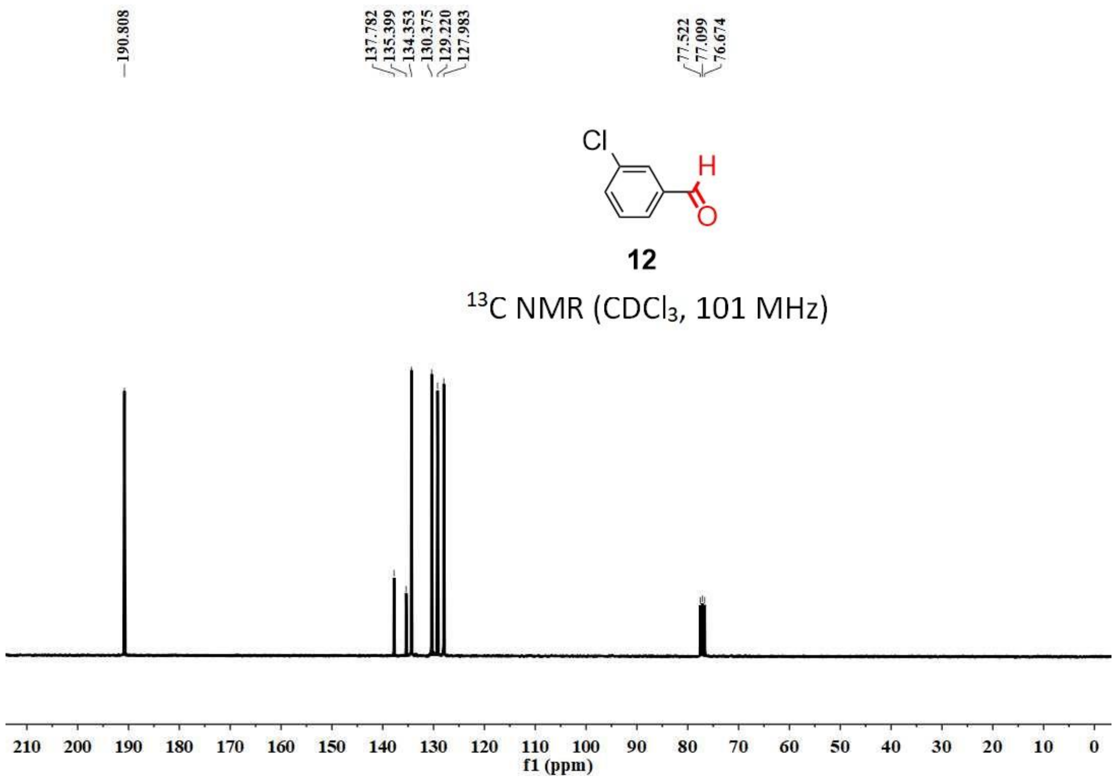

12

^{13}C NMR (CDCl$_3$, 101 MHz)

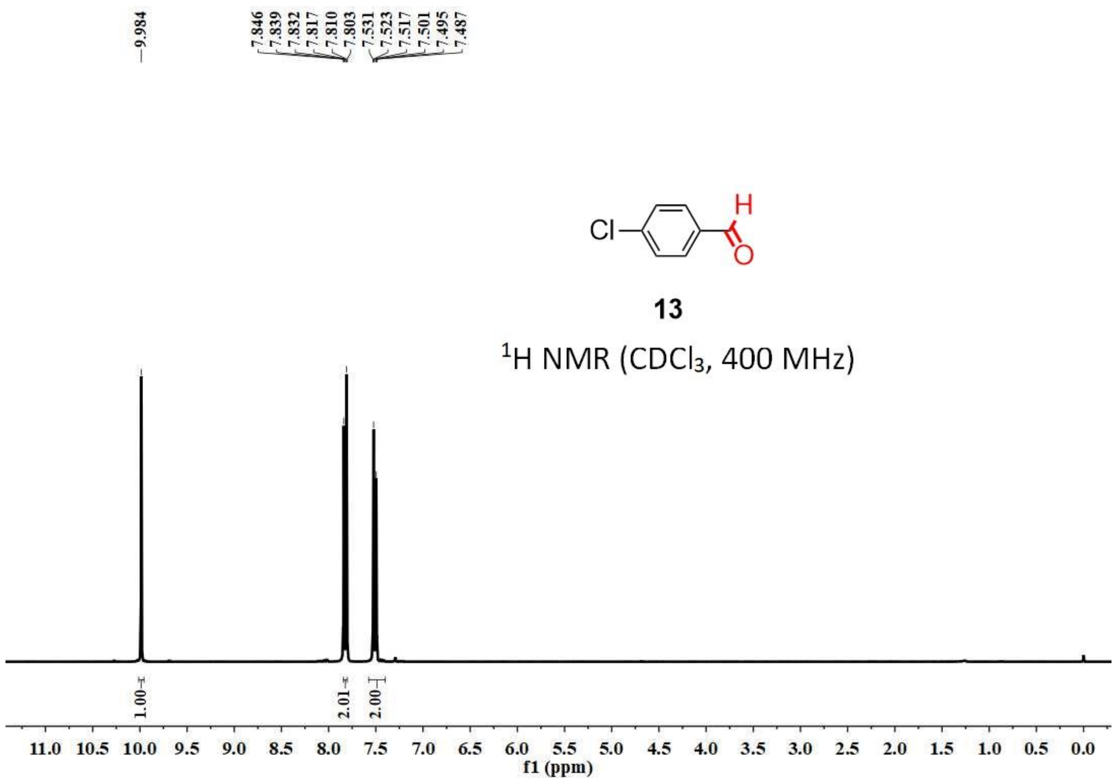
9.984
7.846
7.839
7.832
7.817
7.810
7.803
7.531
7.523
7.517
7.501
7.495
7.487
Cl
H
O
13
^{1}H NMR ($CDCl_3$, 400 MHz)
1.00
2.01
2.00
11.0 10.5 10.0 9.5 9.0 8.5 8.0 7.5 7.0 6.5 6.0 5.5 5.0 4.5 4.0 3.5 3.0 2.5 2.0 1.5 1.0 0.5 0.0
f1 (ppm)

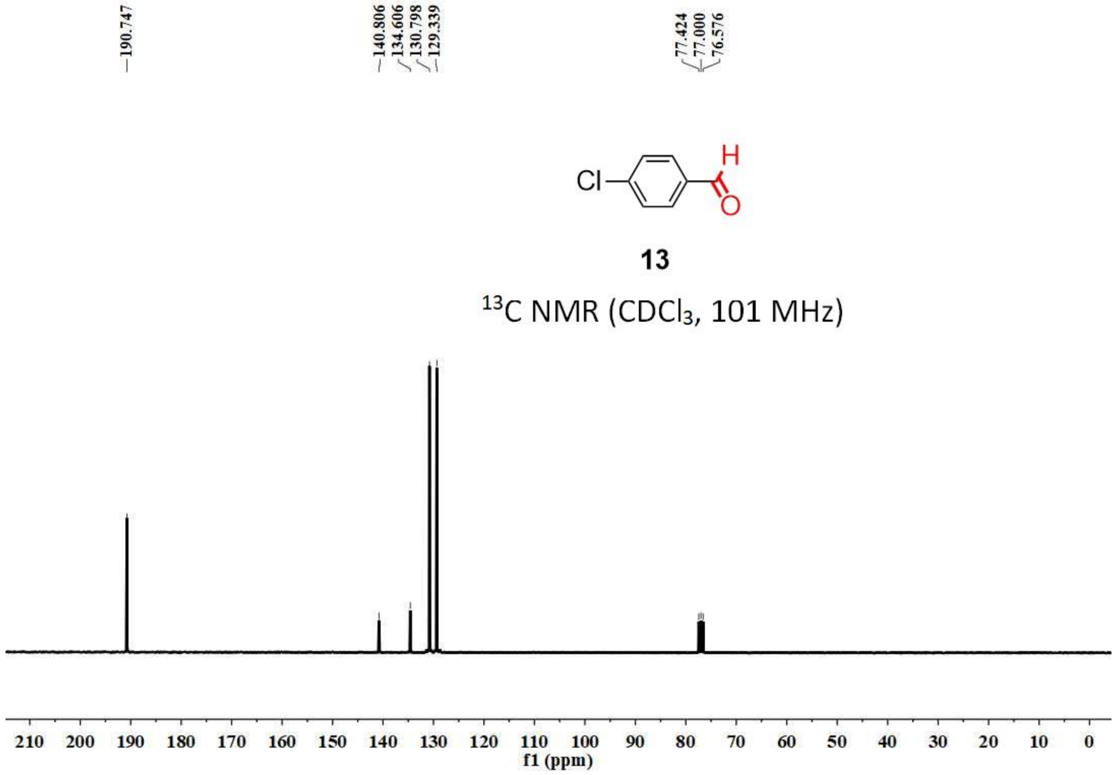
190.747
140.806
134.606
130.798
129.339
77.424
77.000
76.576
Cl
H
O
13
^{13}C NMR ($CDCl_3$, 101 MHz)
210 200 190 180 170 160 150 140 130 120 110 100 90 80 70 60 50 40 30 20 10 0
f1 (ppm)

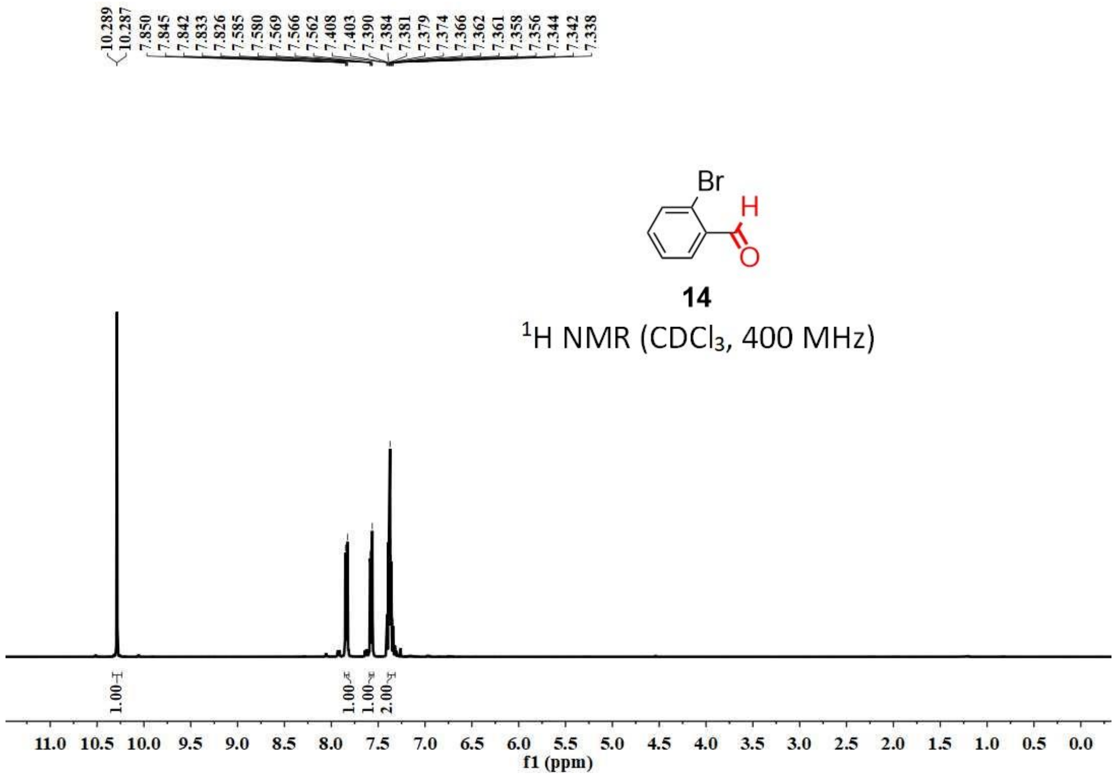
10.289
10.287
7.850
7.845
7.842
7.833
7.826
7.585
7.580
7.569
7.566
7.562
7.408
7.403
7.390
7.384
7.381
7.379
7.374
7.366
7.362
7.361
7.358
7.356
7.344
7.342
7.338
Br
H
O
14
1H NMR (CDCl3, 400 MHz)
1.00
1.00
1.00
2.00
11.0 10.5 10.0 9.5 9.0 8.5 8.0 7.5 7.0 6.5 6.0 5.5 5.0 4.5 4.0 3.5 3.0 2.5 2.0 1.5 1.0 0.5 0.0
f1 (ppm)

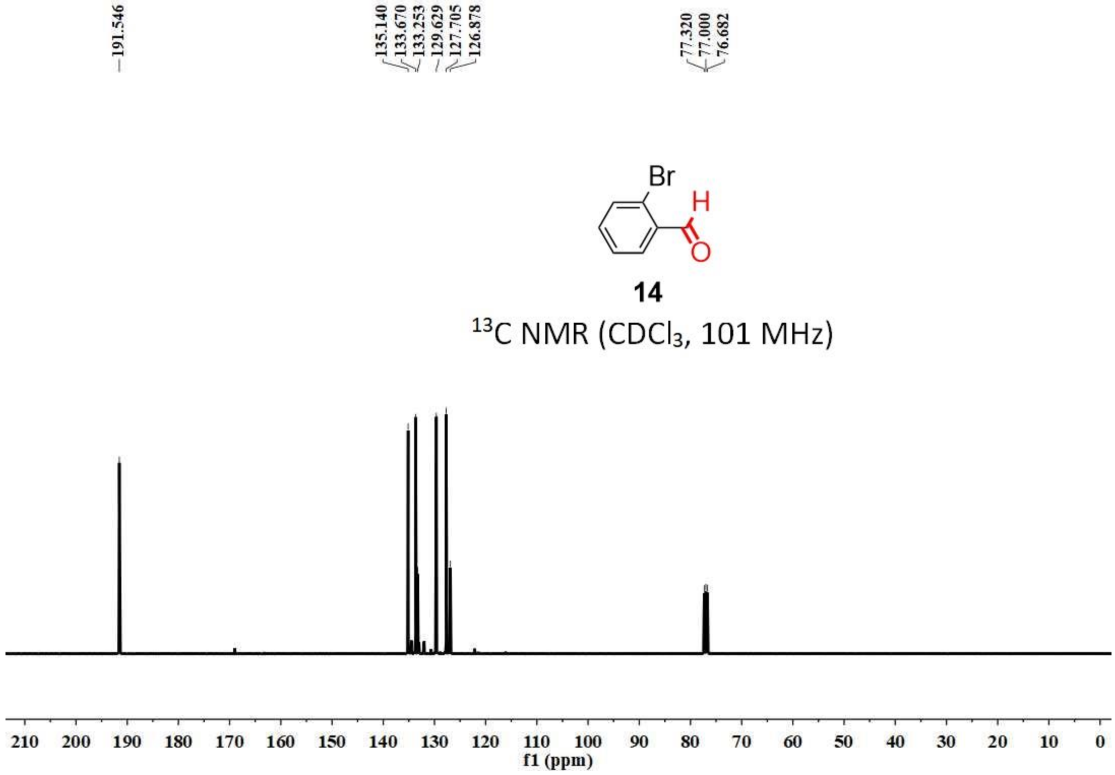
191.546
135.140
133.670
133.253
129.629
127.705
126.878
77.320
77.000
76.682
Br
H
O
14
13C NMR (CDCl3, 101 MHz)
210 200 190 180 170 160 150 140 130 120 110 100 90 80 70 60 50 40 30 20 10 0
f1 (ppm)

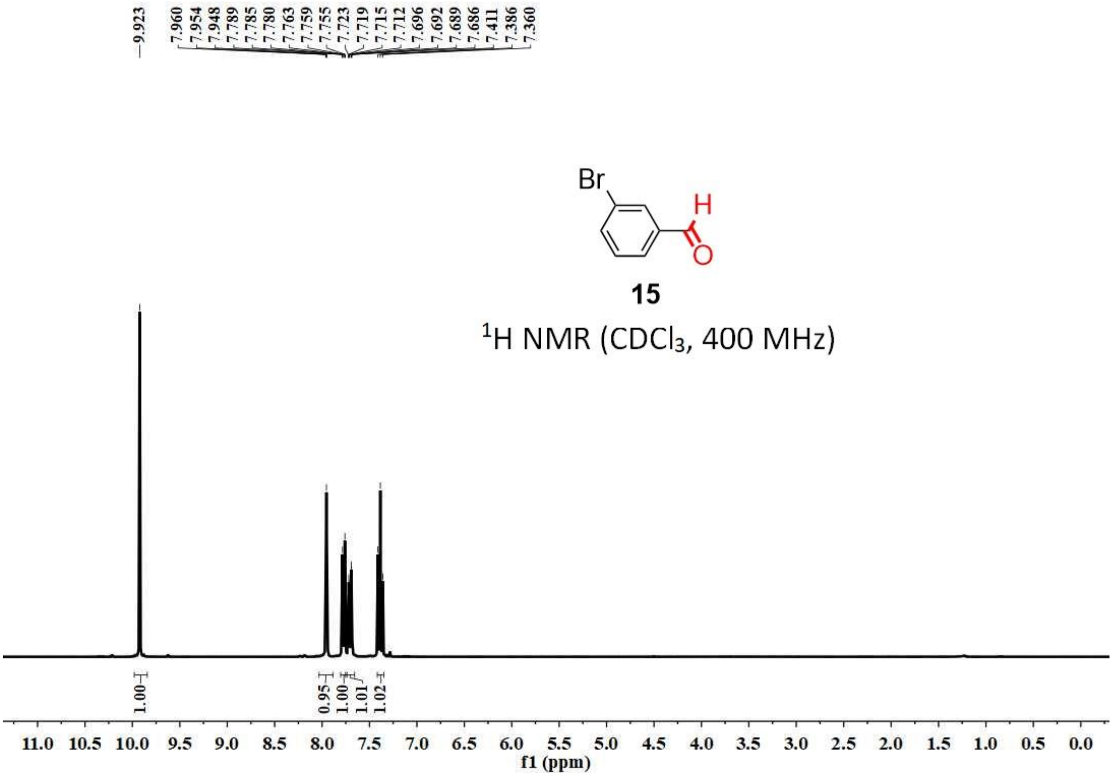
9.923
7.960 7.954 7.948 7.789 7.785 7.780 7.763 7.759 7.755 7.723 7.719 7.715 7.712 7.696 7.692 7.689 7.686 7.411 7.386 7.360
Br
H
O
15
1H NMR (CDCl3, 400 MHz)
1.00
0.95 1.00 1.01 1.02
11.0 10.5 10.0 9.5 9.0 8.5 8.0 7.5 7.0 6.5 6.0 5.5 5.0 4.5 4.0 3.5 3.0 2.5 2.0 1.5 1.0 0.5 0.0
f1 (ppm)

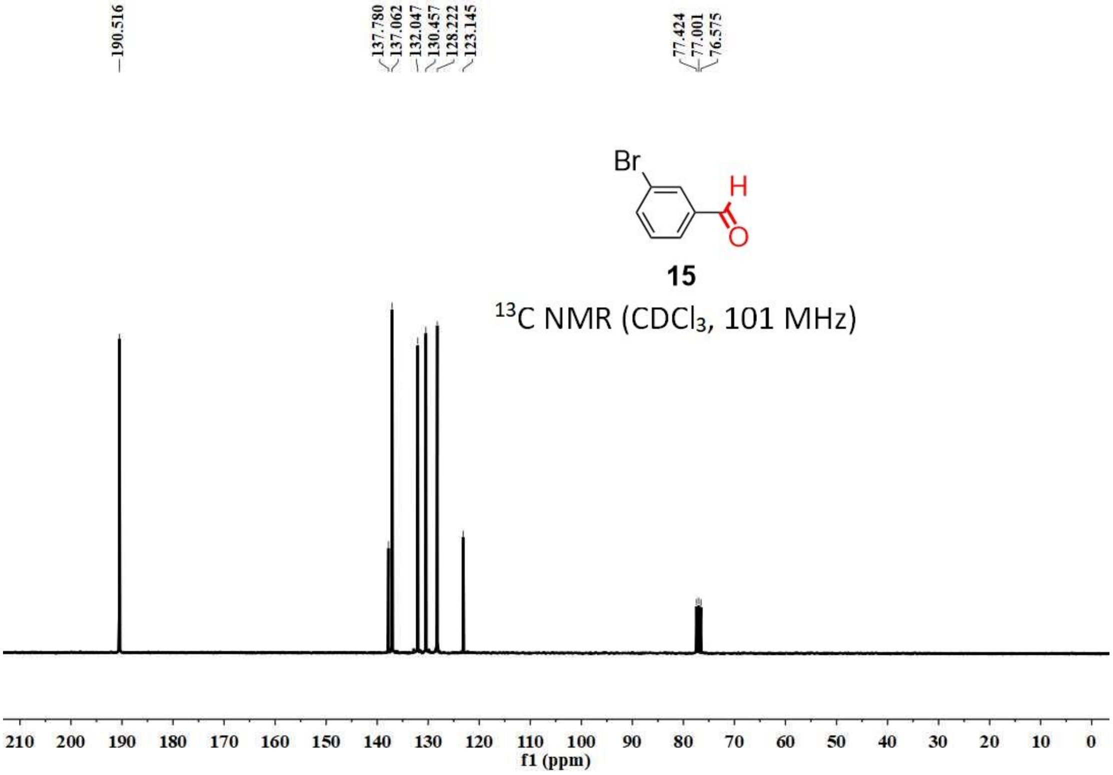
190.516
137.780 137.062 132.047 130.457 128.222 123.145
77.424 77.001 76.575
Br
H
O
15
13C NMR (CDCl3, 101 MHz)
210 200 190 180 170 160 150 140 130 120 110 100 90 80 70 60 50 40 30 20 10 0
f1 (ppm)

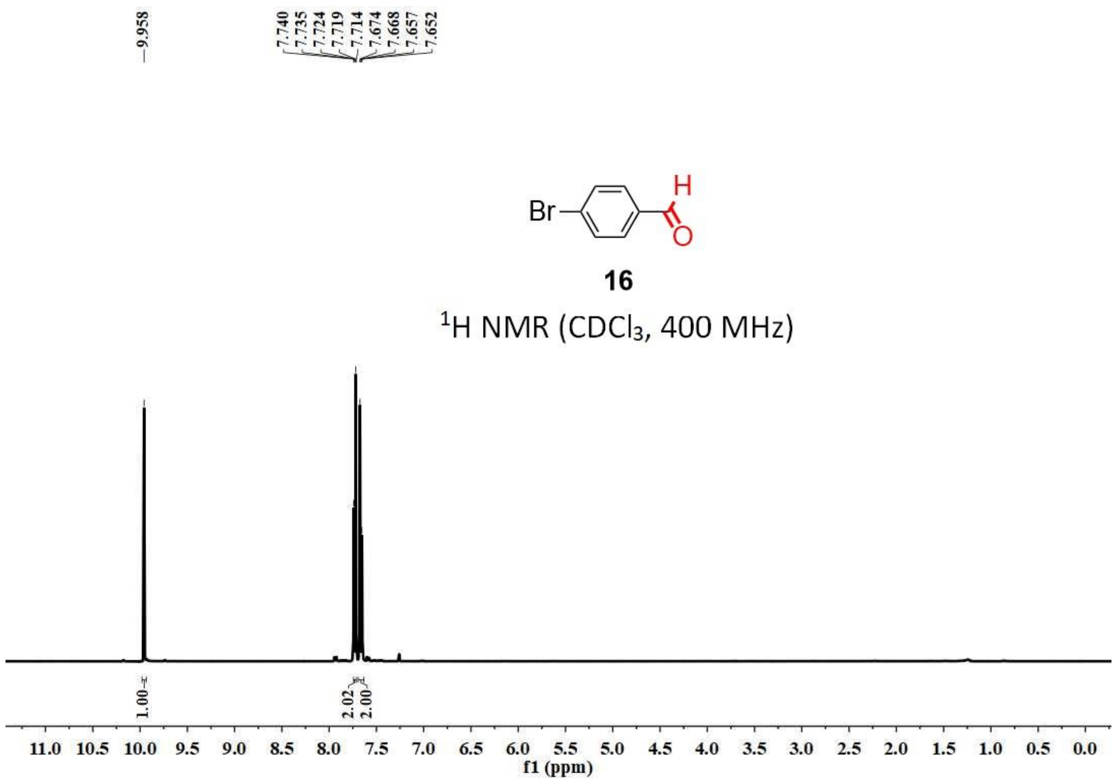

9.958
7.740
7.735
7.724
7.719
7.714
7.674
7.668
7.657
7.652
Br
H
O
16
¹H NMR (CDCl₃, 400 MHz)
1.00
2.02
2.00
11.0 10.5 10.0 9.5 9.0 8.5 8.0 7.5 7.0 6.5 6.0 5.5 5.0 4.5 4.0 3.5 3.0 2.5 2.0 1.5 1.0 0.5 0.0
f1 (ppm)

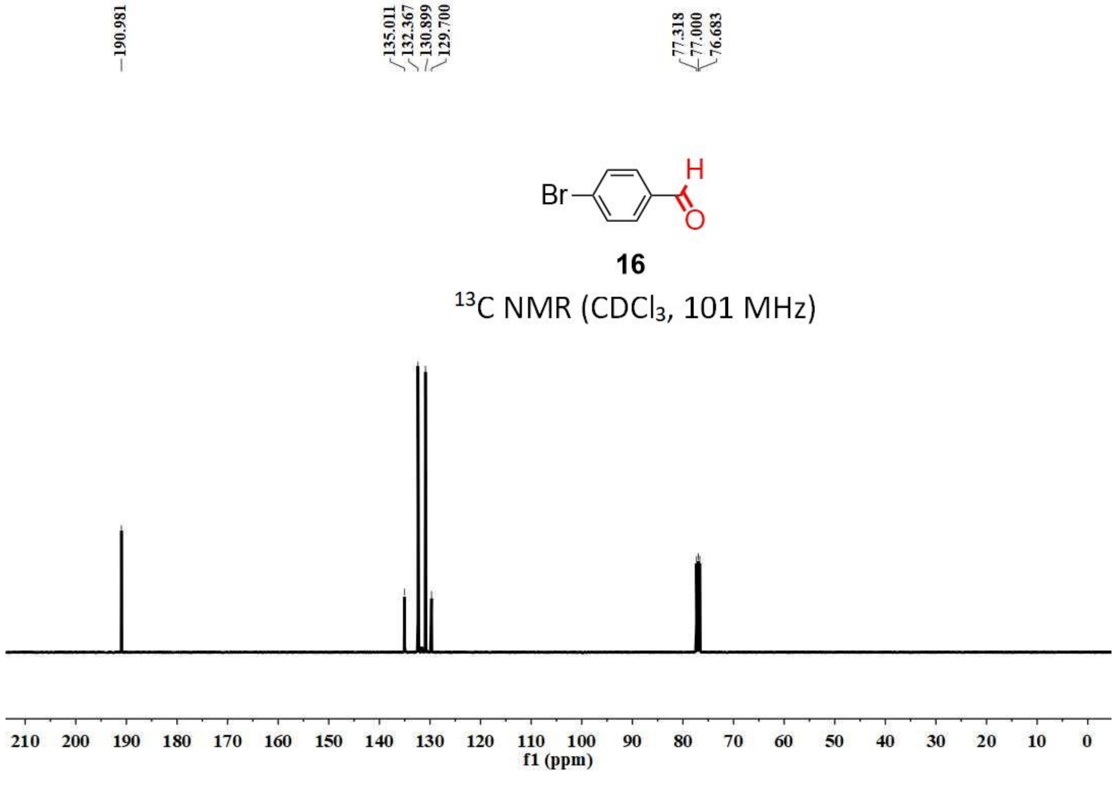

190.981
135.011
132.367
130.899
129.700
77.318
77.000
76.683
Br
H
O
16
¹³C NMR (CDCl₃, 101 MHz)
210 200 190 180 170 160 150 140 130 120 110 100 90 80 70 60 50 40 30 20 10 0
f1 (ppm)

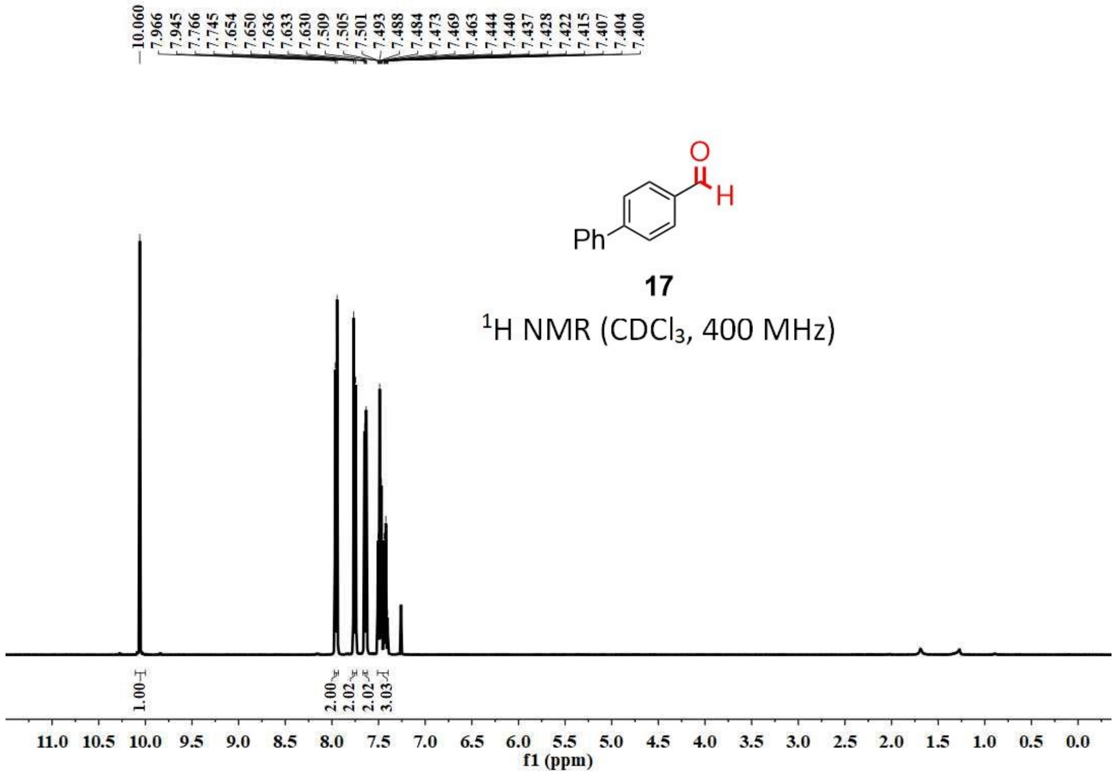

17

^{1}H NMR ($CDCl_3$, 400 MHz)

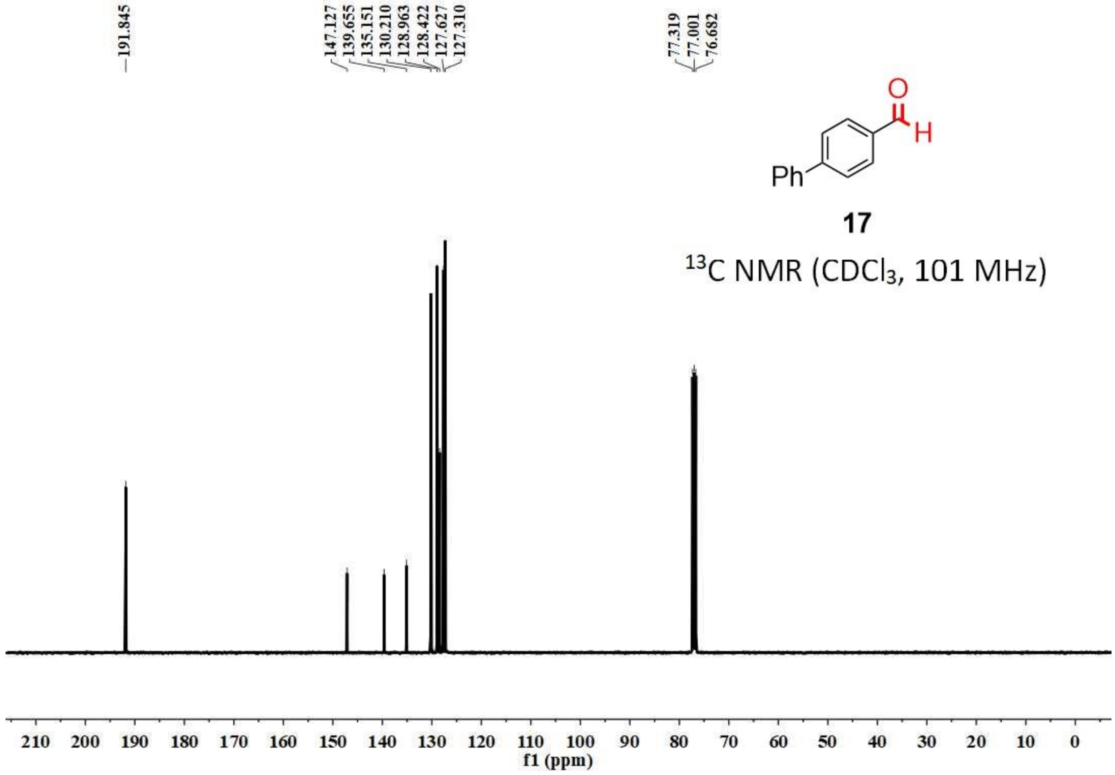

17

^{13}C NMR ($CDCl_3$, 101 MHz)

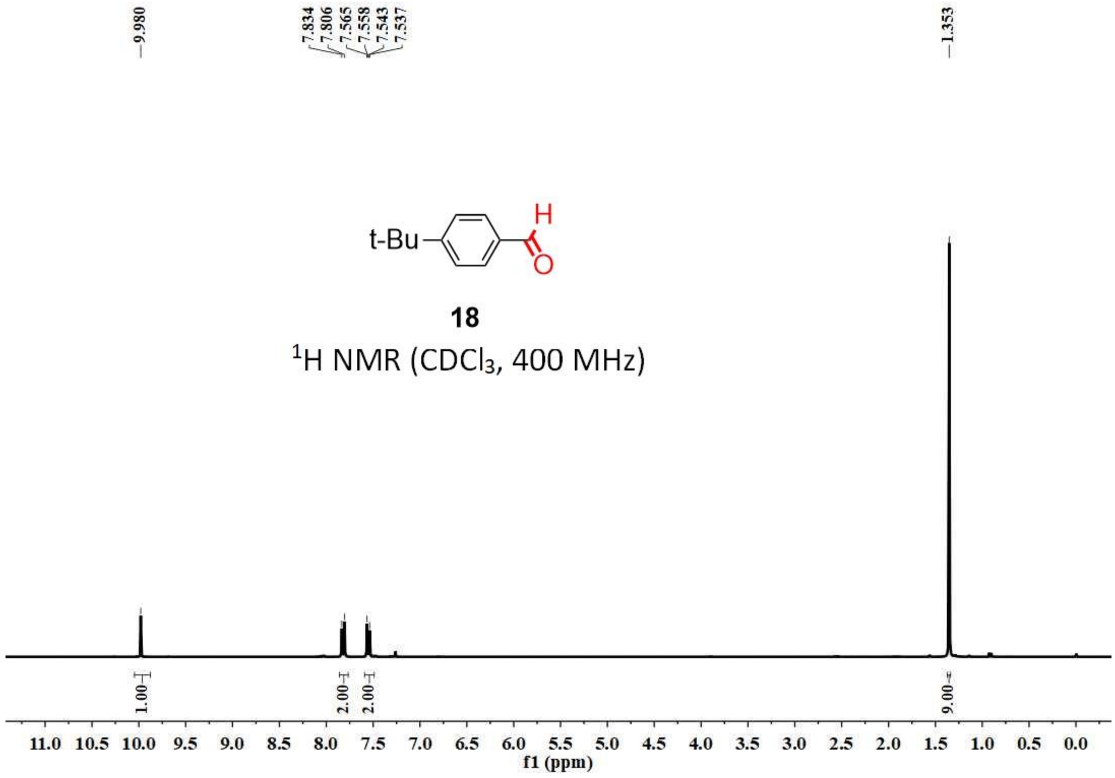
9.980
7.834
7.806
7.565
7.558
7.543
7.537
1.353
t-Bu
H
O
18
1H NMR (CDCl3, 400 MHz)
1.00
2.00
2.00
9.00
11.0 10.5 10.0 9.5 9.0 8.5 8.0 7.5 7.0 6.5 6.0 5.5 5.0 4.5 4.0 3.5 3.0 2.5 2.0 1.5 1.0 0.5 0.0
f1 (ppm)

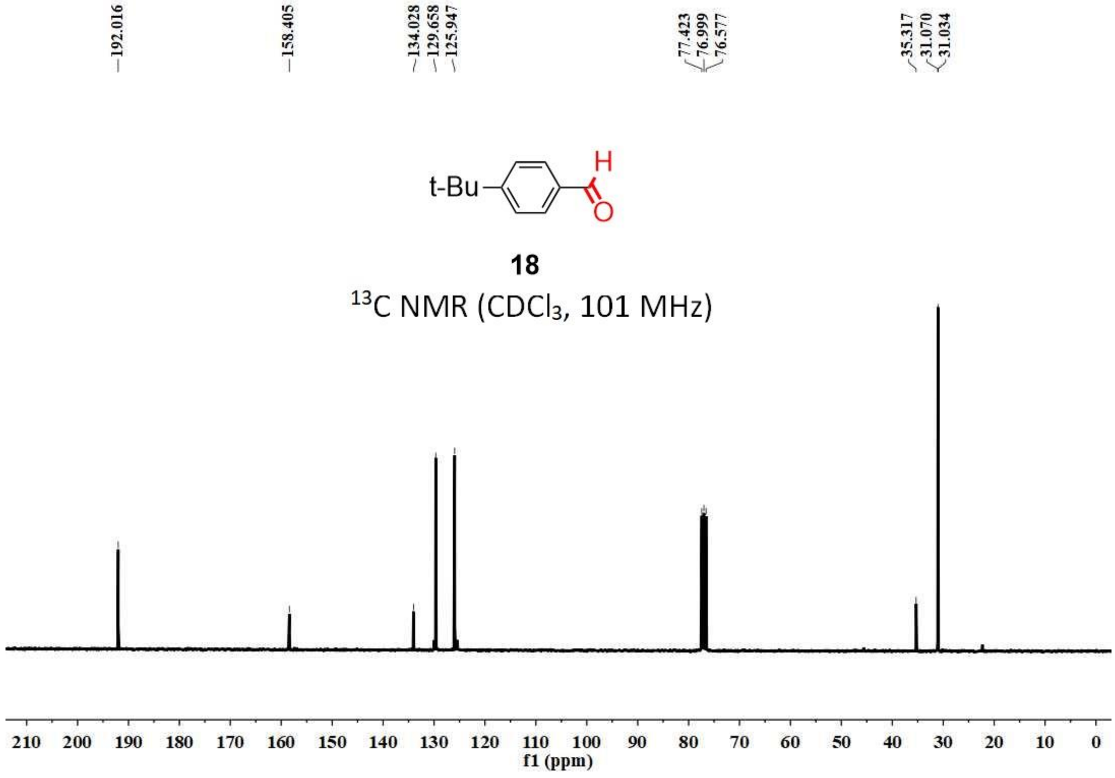
192.016
158.405
134.028
129.658
125.947
77.423
76.999
76.577
35.317
31.070
31.034
t-Bu
H
O
18
13C NMR (CDCl3, 101 MHz)
210 200 190 180 170 160 150 140 130 120 110 100 90 80 70 60 50 40 30 20 10 0
f1 (ppm)

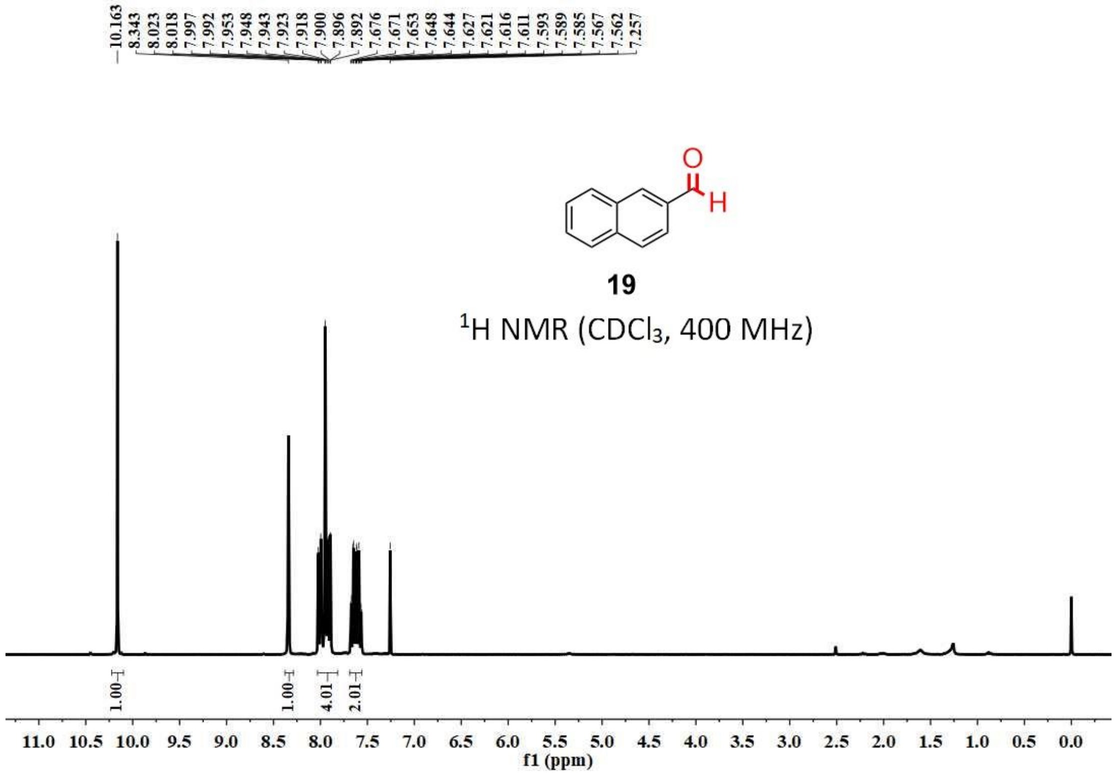
19
¹H NMR (CDCl₃, 400 MHz)
f1 (ppm)

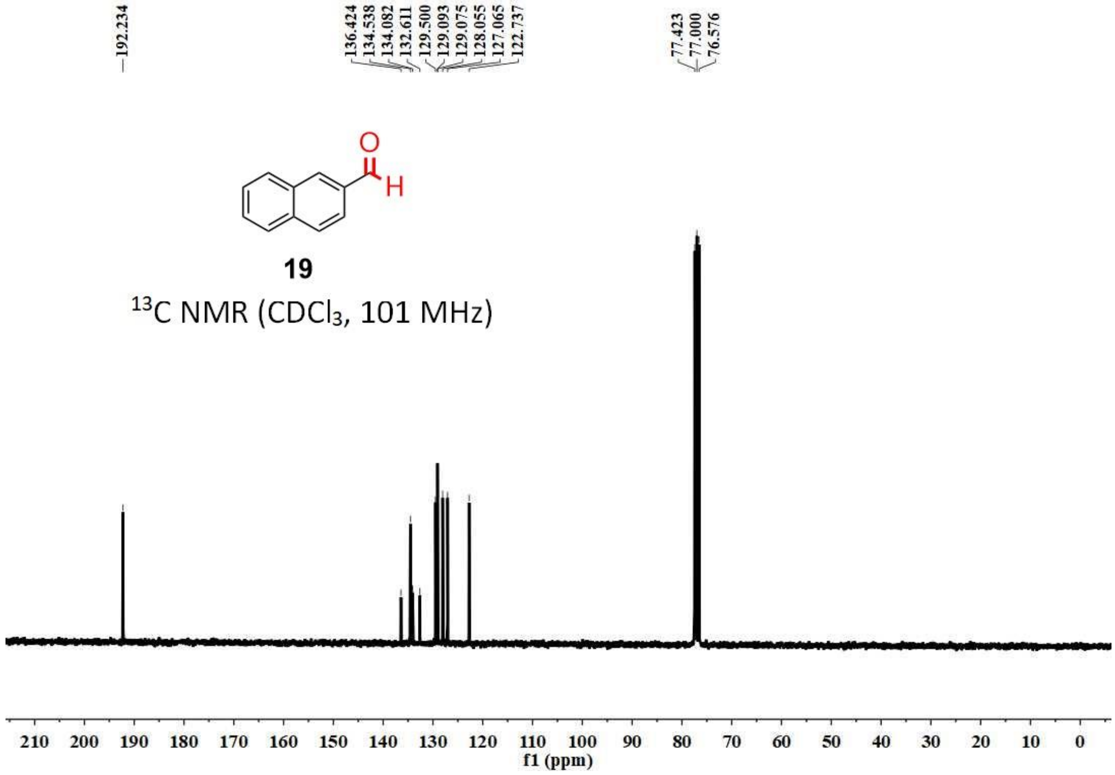
19
¹³C NMR (CDCl₃, 101 MHz)
f1 (ppm)

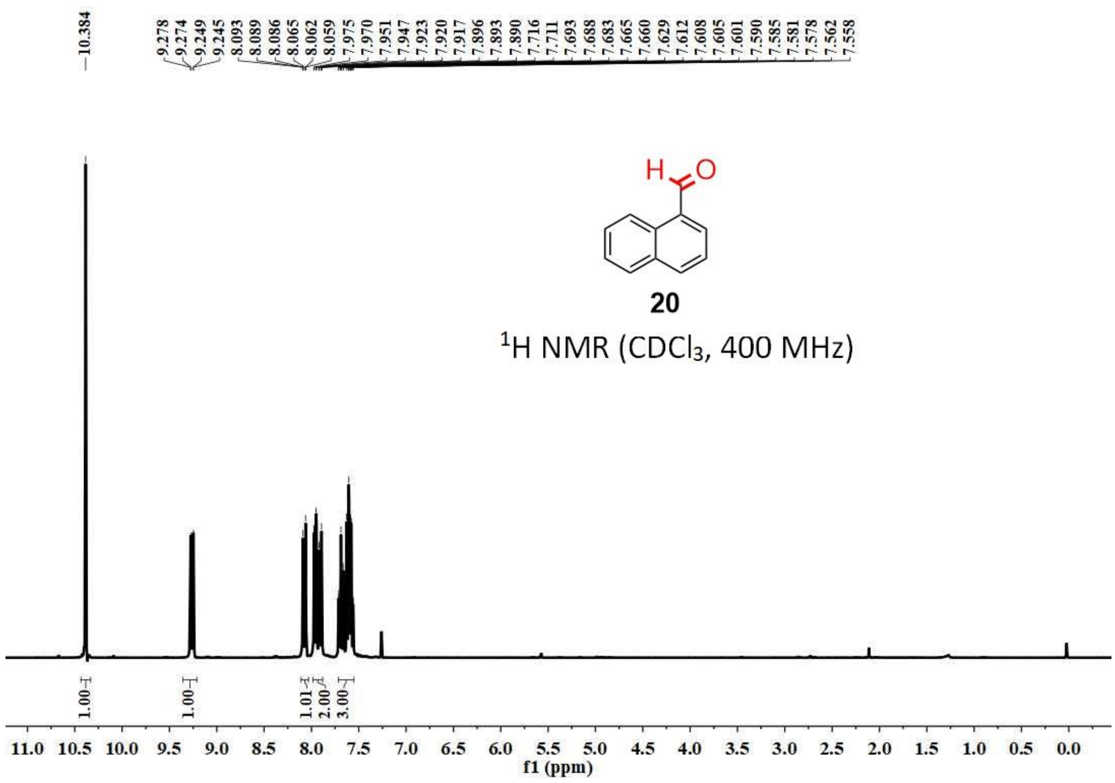
10.384
9.278
9.274
9.249
9.245
8.093
8.089
8.086
8.065
8.062
8.059
7.975
7.970
7.951
7.947
7.923
7.920
7.917
7.896
7.893
7.890
7.716
7.711
7.693
7.688
7.683
7.665
7.660
7.629
7.612
7.608
7.605
7.601
7.590
7.585
7.581
7.578
7.562
7.558
H
O
20
1H NMR (CDCl3, 400 MHz)
1.00
1.00
1.01
2.00
3.00
11.0 10.5 10.0 9.5 9.0 8.5 8.0 7.5 7.0 6.5 6.0 5.5 5.0 4.5 4.0 3.5 3.0 2.5 2.0 1.5 1.0 0.5 0.0
f1 (ppm)

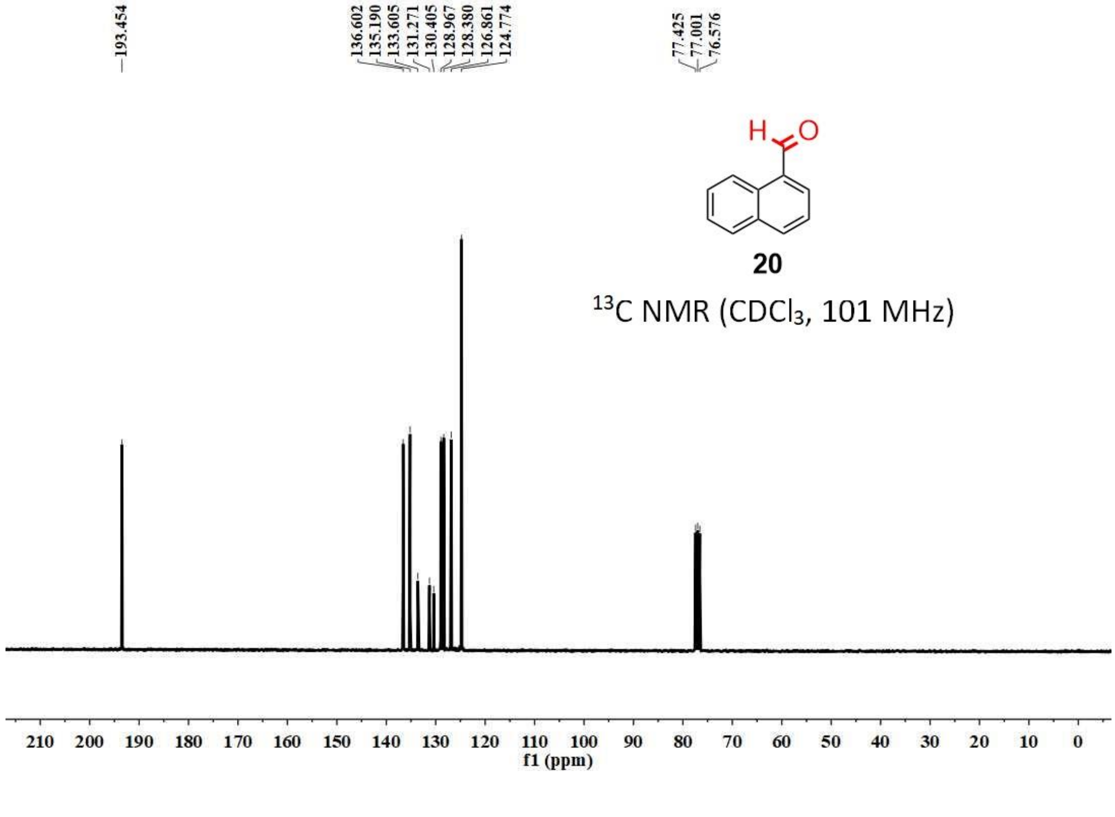
193.454
136.602
135.190
133.605
131.271
130.405
128.967
128.380
126.861
124.774
77.425
77.001
76.576
H
O
20
13C NMR (CDCl3, 101 MHz)
210 200 190 180 170 160 150 140 130 120 110 100 90 80 70 60 50 40 30 20 10 0
f1 (ppm)

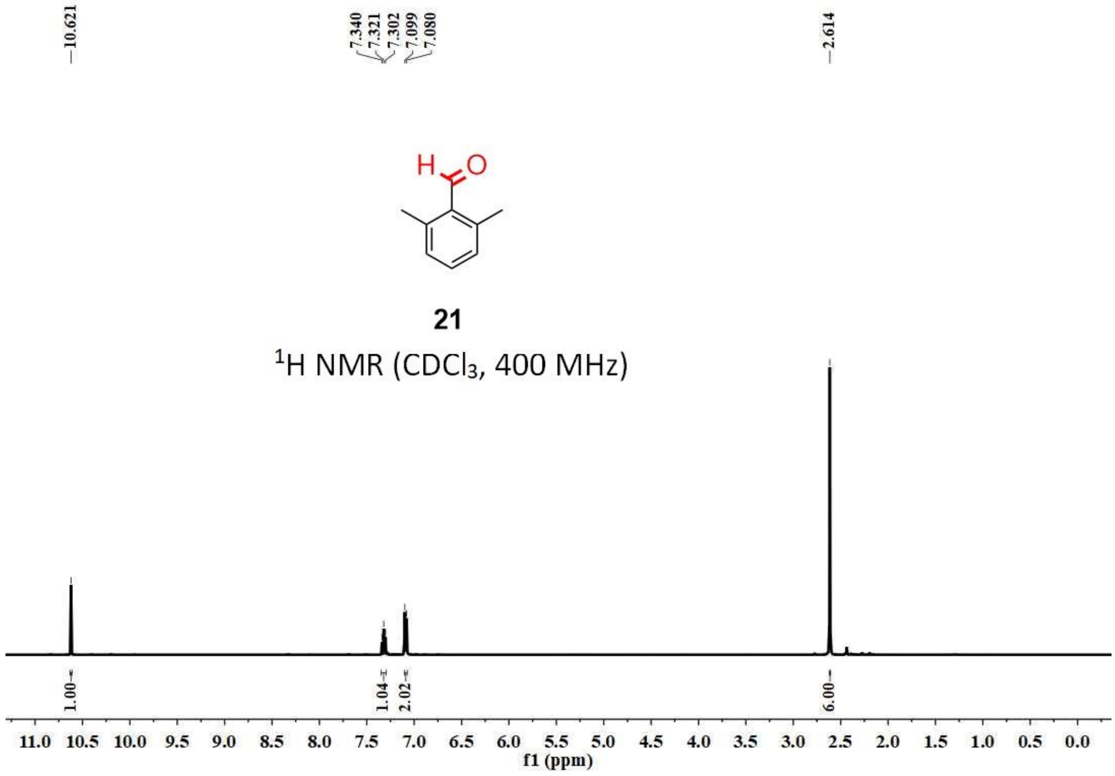

10.621
7.340
7.321
7.302
7.099
7.080
2.614
21
^1H NMR ($CDCl_3$, 400 MHz)
1.00
1.04
2.02
6.00
11.0 10.5 10.0 9.5 9.0 8.5 8.0 7.5 7.0 6.5 6.0 5.5 5.0 4.5 4.0 3.5 3.0 2.5 2.0 1.5 1.0 0.5 0.0
f1 (ppm)

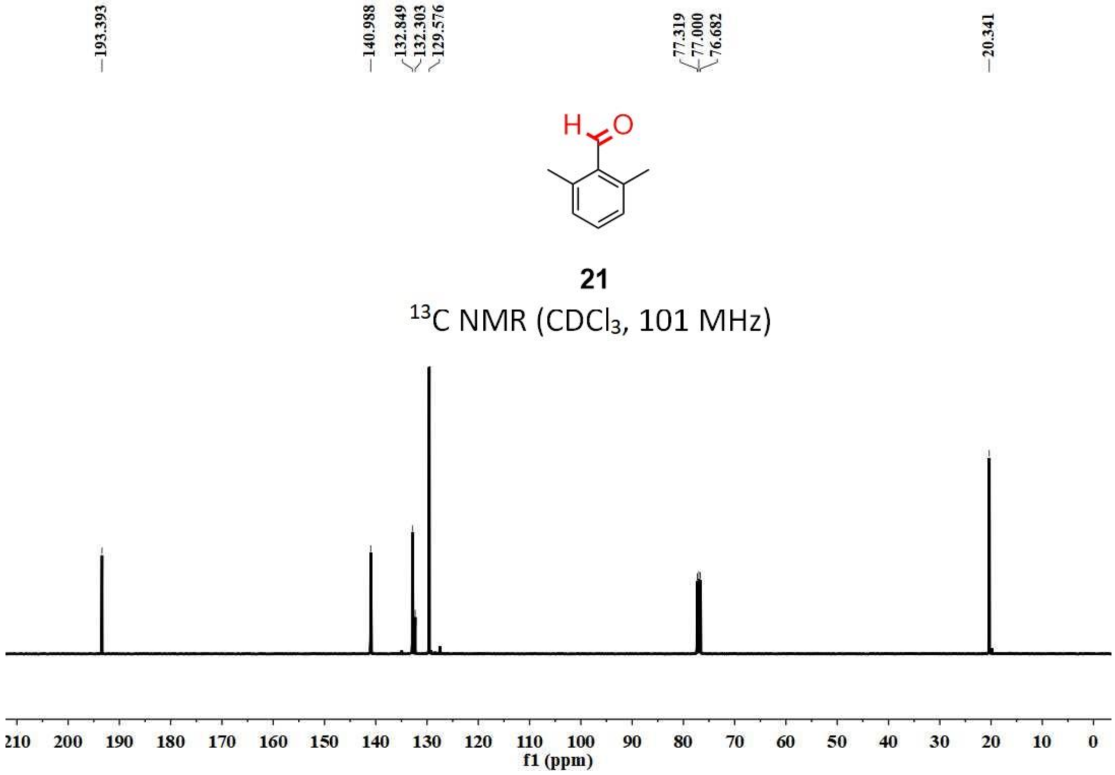

193.393
140.988
132.849
132.303
129.576
77.319
77.000
76.682
20.341
21
^{13}C NMR ($CDCl_3$, 101 MHz)
210 200 190 180 170 160 150 140 130 120 110 100 90 80 70 60 50 40 30 20 10 0
f1 (ppm)

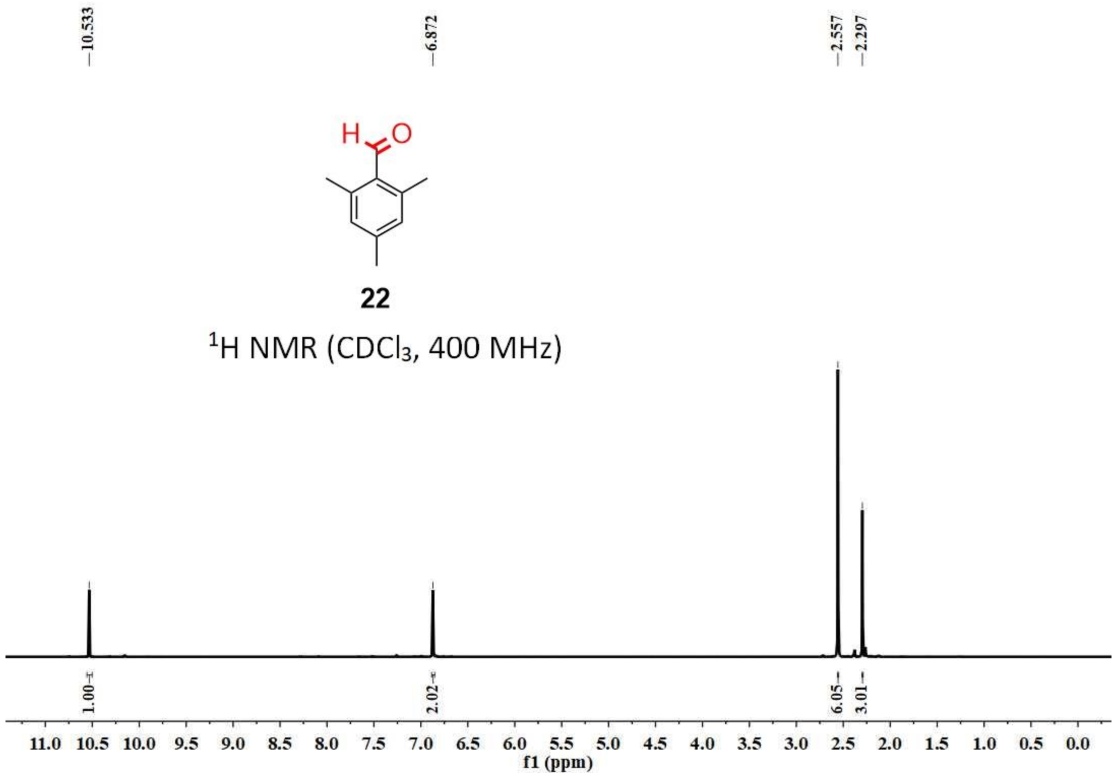
10.533
6.872
2.557
2.297
22
^{1}H NMR ($CDCl_3$, 400 MHz)
1.00
2.02
6.05
3.01
11.0 10.5 10.0 9.5 9.0 8.5 8.0 7.5 7.0 6.5 6.0 5.5 5.0 4.5 4.0 3.5 3.0 2.5 2.0 1.5 1.0 0.5 0.0
f1 (ppm)

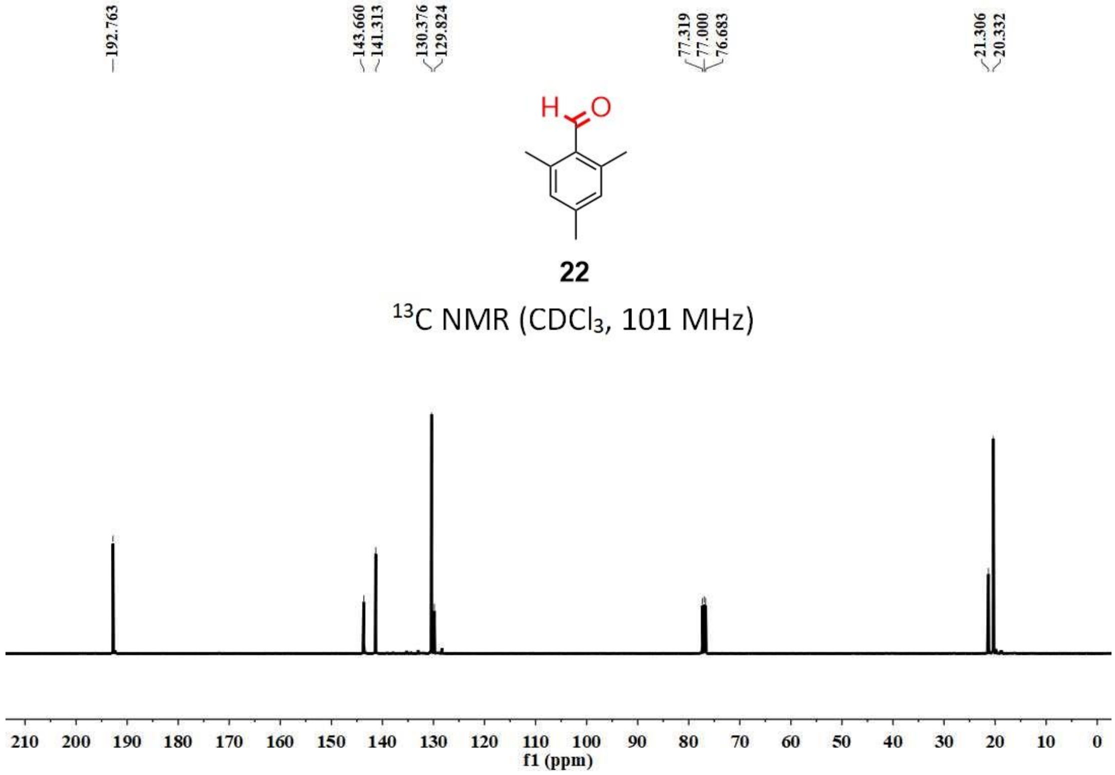
192.763
143.660
141.313
130.376
129.824
77.319
77.000
76.683
21.306
20.332
22
^{13}C NMR ($CDCl_3$, 101 MHz)
210 200 190 180 170 160 150 140 130 120 110 100 90 80 70 60 50 40 30 20 10 0
f1 (ppm)

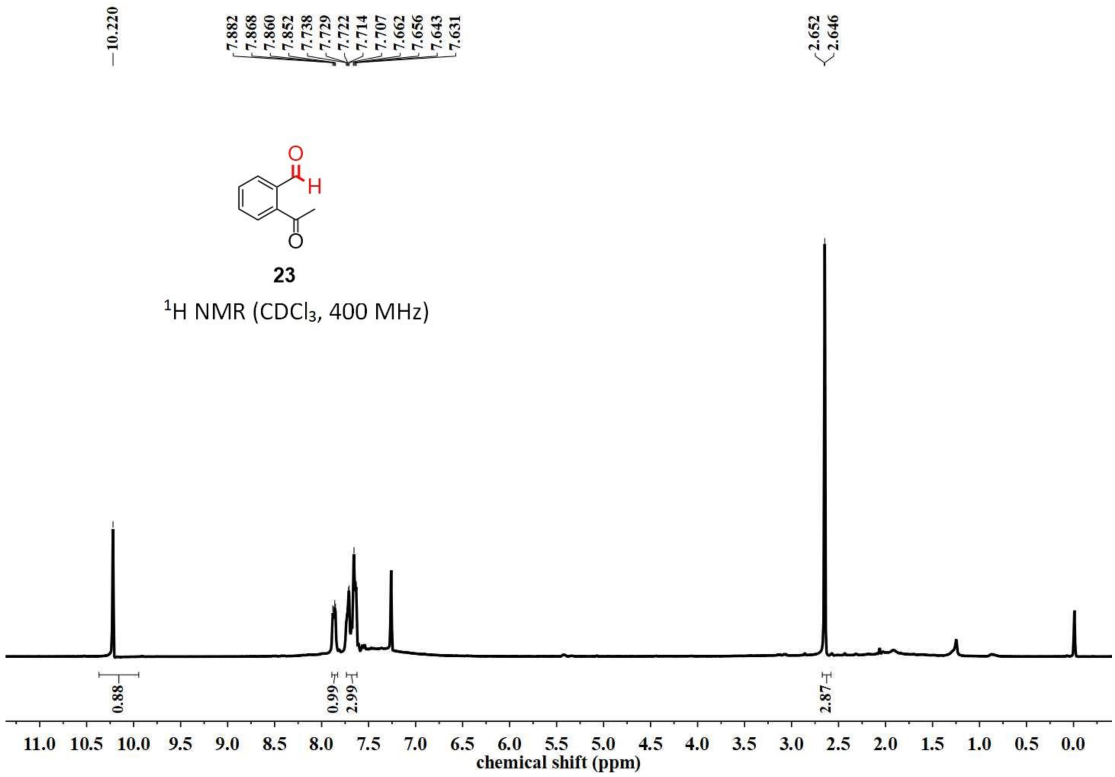
10.220
7.882 7.868 7.860 7.852 7.738 7.729 7.722 7.714 7.707 7.662 7.656 7.643 7.631
2.652 2.646
23
1H NMR (CDCl3, 400 MHz)
0.88
0.99
2.99
2.87
11.0 10.5 10.0 9.5 9.0 8.5 8.0 7.5 7.0 6.5 6.0 5.5 5.0 4.5 4.0 3.5 3.0 2.5 2.0 1.5 1.0 0.5 0.0
chemical shift (ppm)

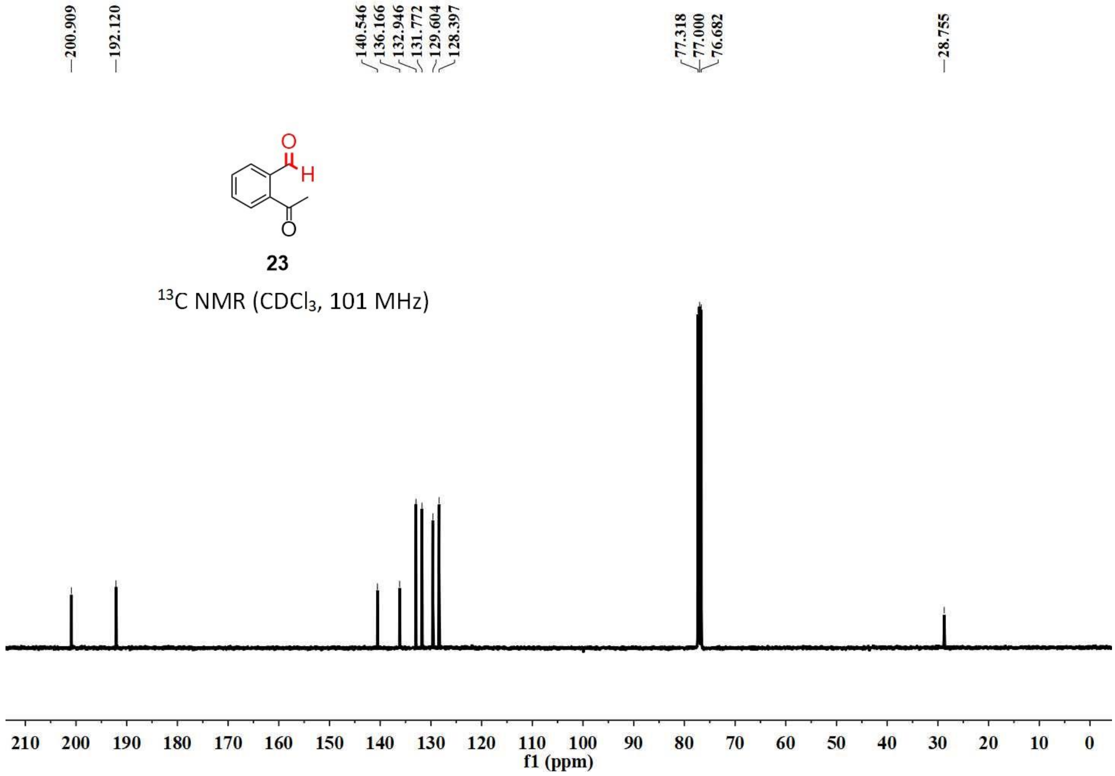
200.909
192.120
140.546 136.166 132.946 131.772 129.604 128.397
77.318 77.000 76.682
28.755
23
13C NMR (CDCl3, 101 MHz)
210 200 190 180 170 160 150 140 130 120 110 100 90 80 70 60 50 40 30 20 10 0
f1 (ppm)

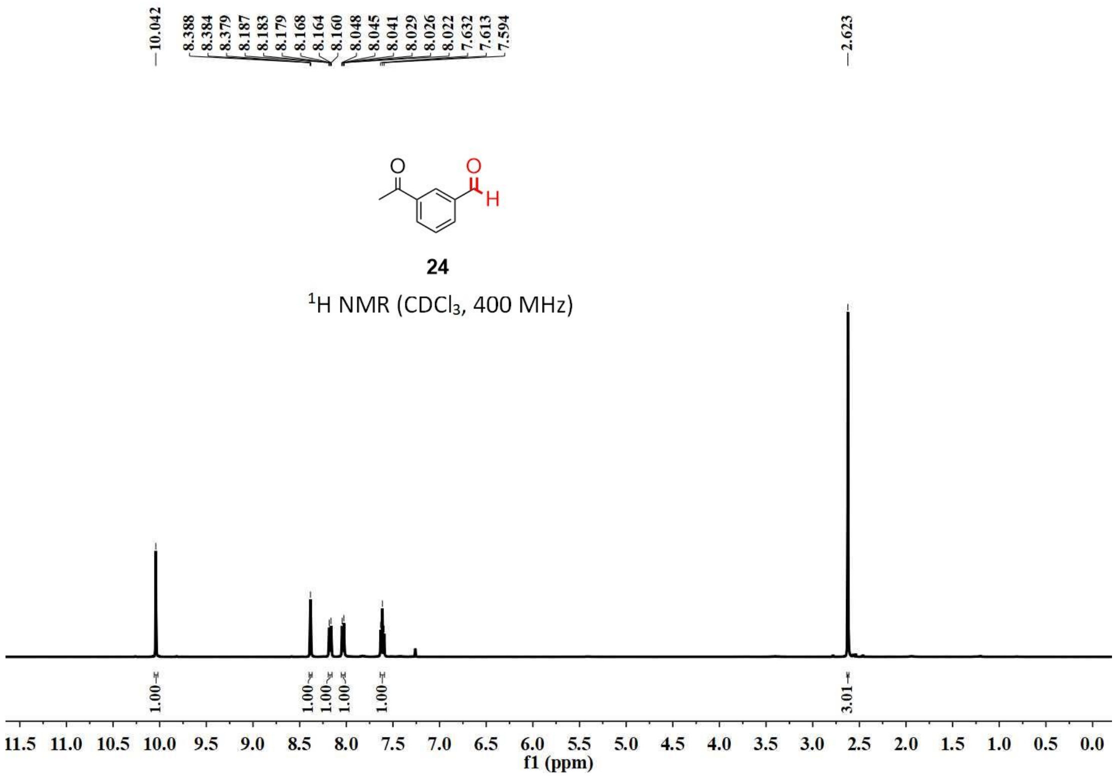

24

^{1}H NMR ($CDCl_3$, 400 MHz)

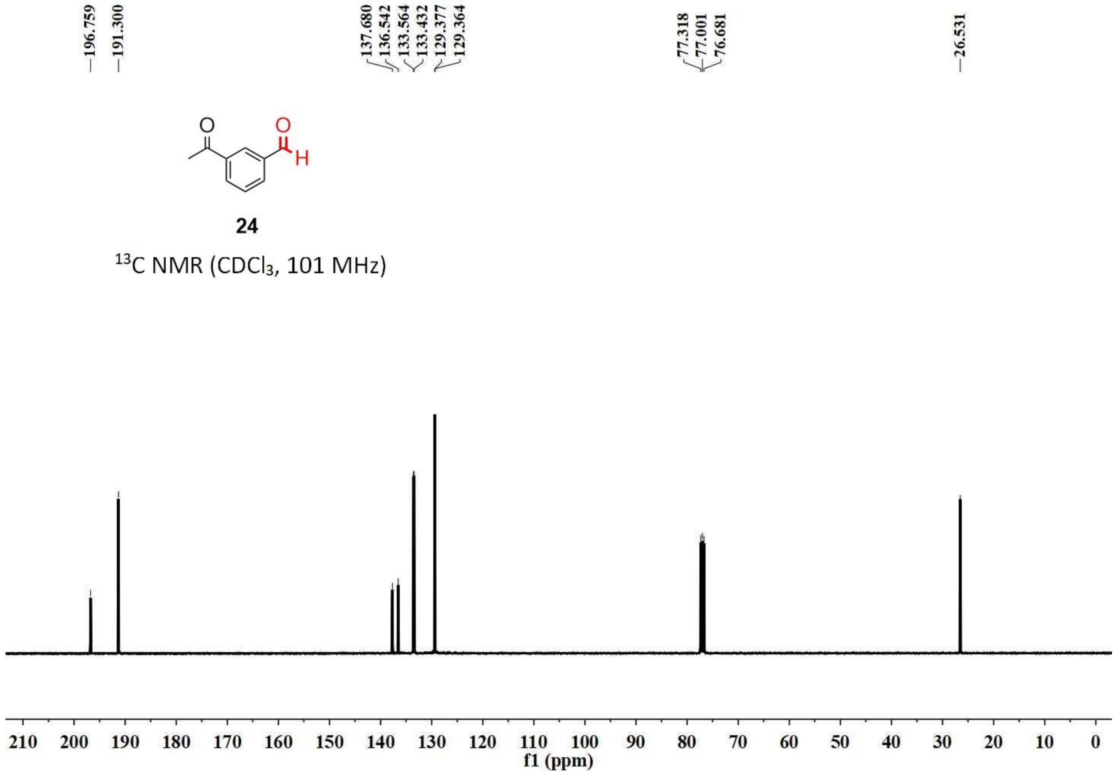

24

^{13}C NMR ($CDCl_3$, 101 MHz)

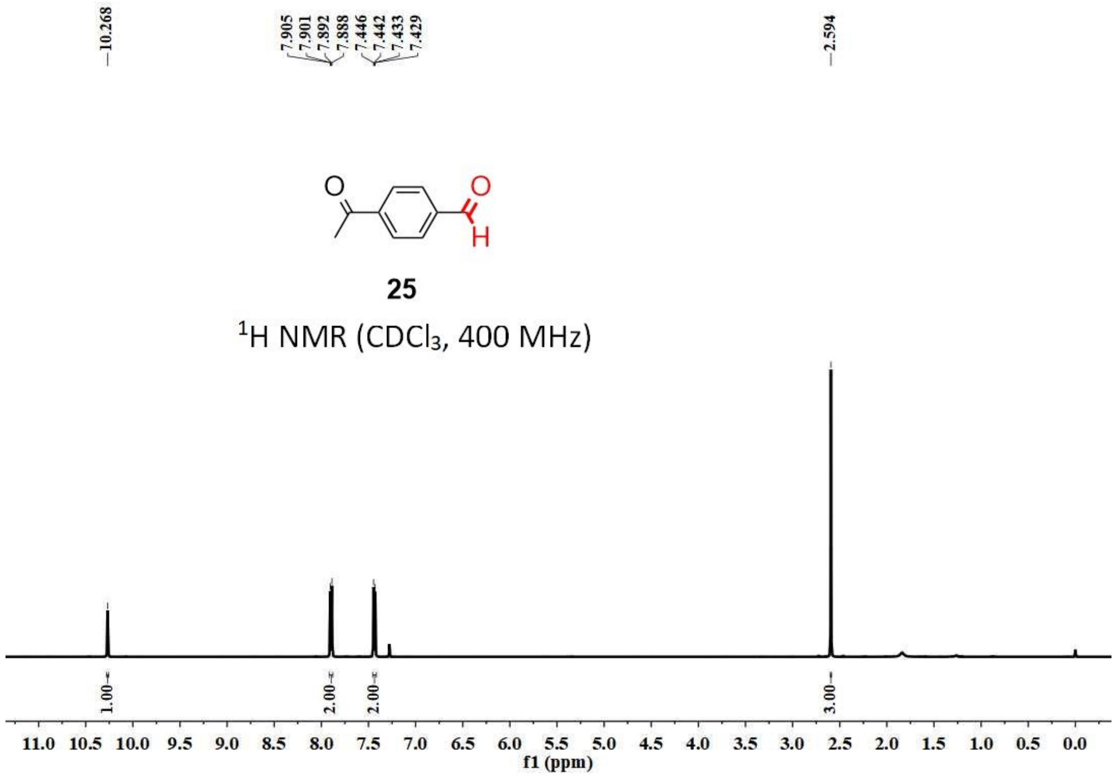

25

^{1}H NMR ($CDCl_3$, 400 MHz)

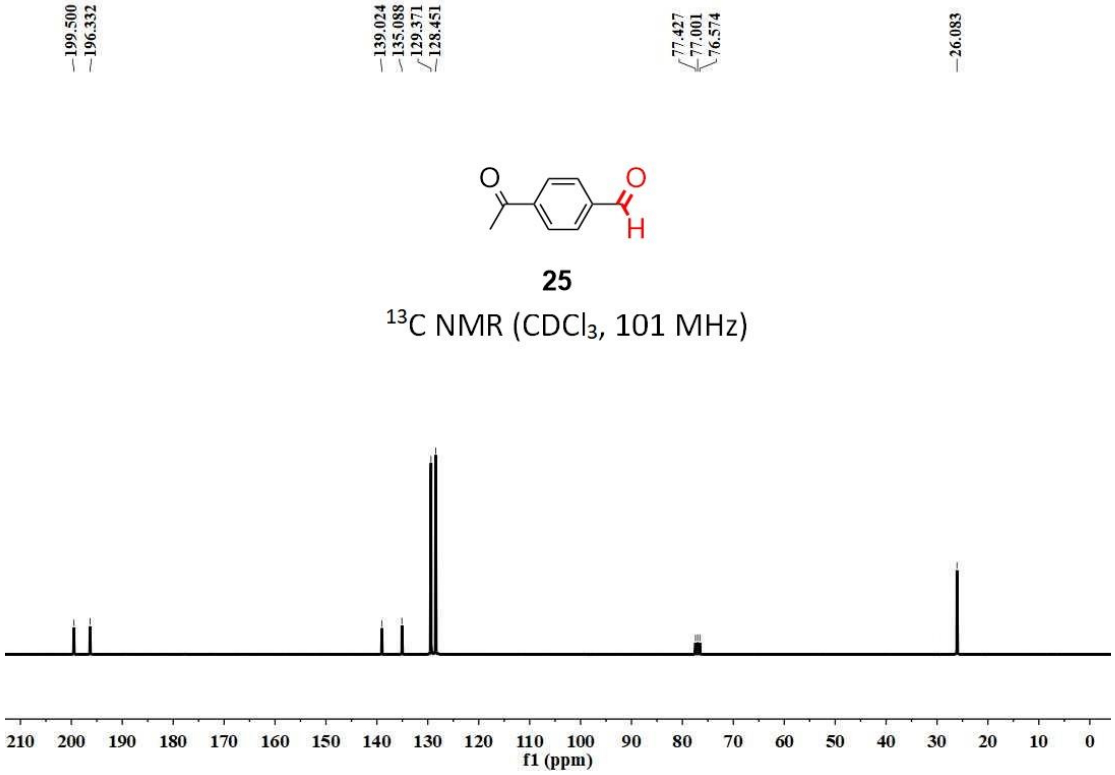

25

^{13}C NMR ($CDCl_3$, 101 MHz)

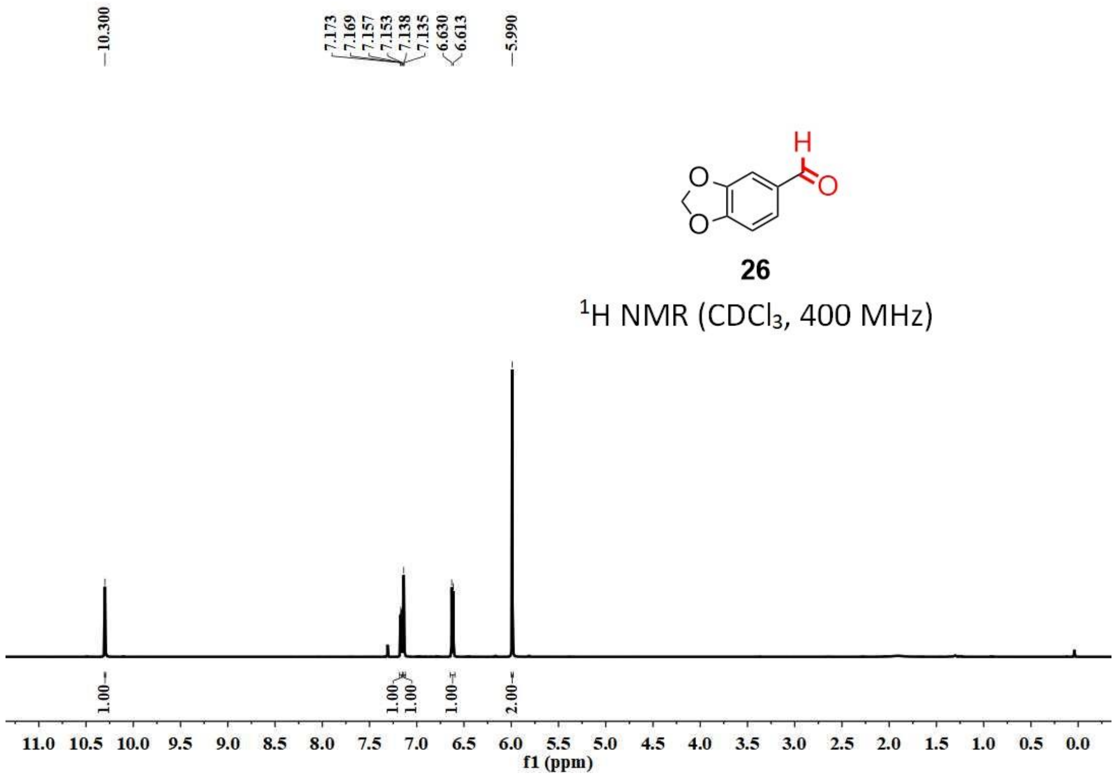
10.300
7.173
7.169
7.157
7.153
7.138
7.135
6.630
6.613
5.990
H
O
O
O
26
1H NMR (CDCl3, 400 MHz)
1.00
1.00
1.00
1.00
2.00
11.0
10.5
10.0
9.5
9.0
8.5
8.0
7.5
7.0
6.5
6.0
5.5
5.0
4.5
4.0
3.5
3.0
2.5
2.0
1.5
1.0
0.5
0.0
f1 (ppm)

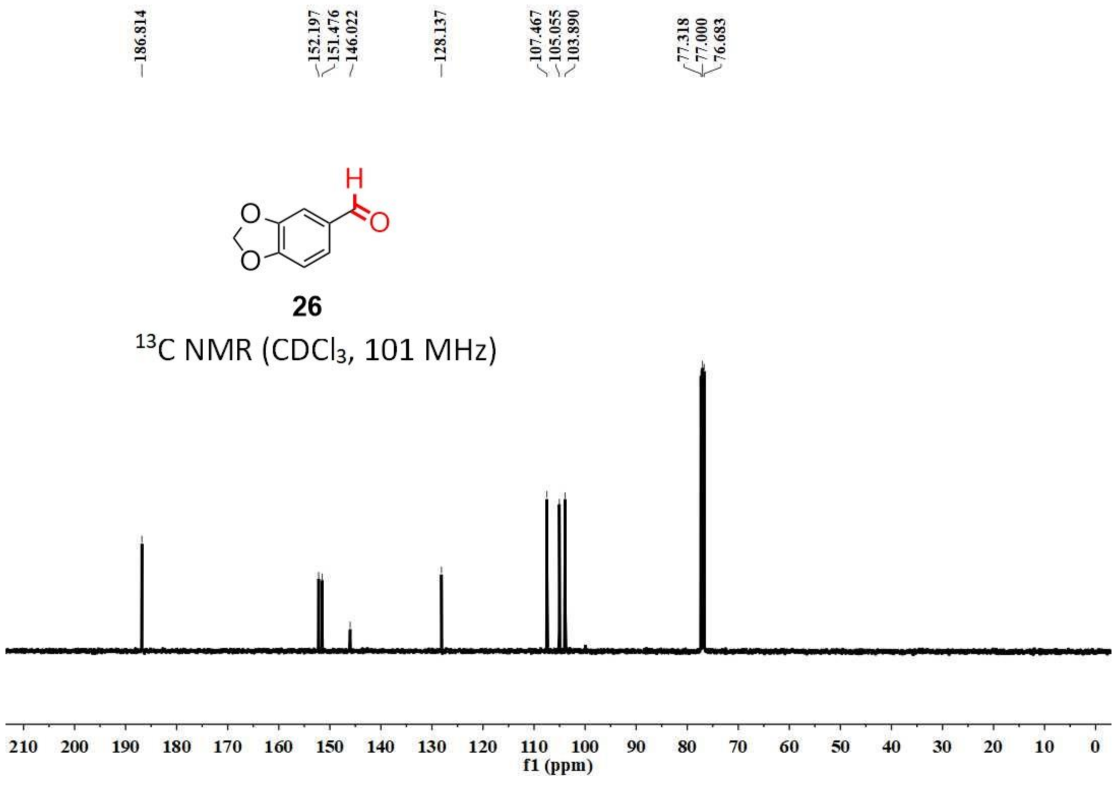
186.814
152.197
151.476
146.022
128.137
107.467
105.055
103.890
77.318
77.000
76.683
H
O
O
O
26
13C NMR (CDCl3, 101 MHz)
210
200
190
180
170
160
150
140
130
120
110
100
90
80
70
60
50
40
30
20
10
0
f1 (ppm)

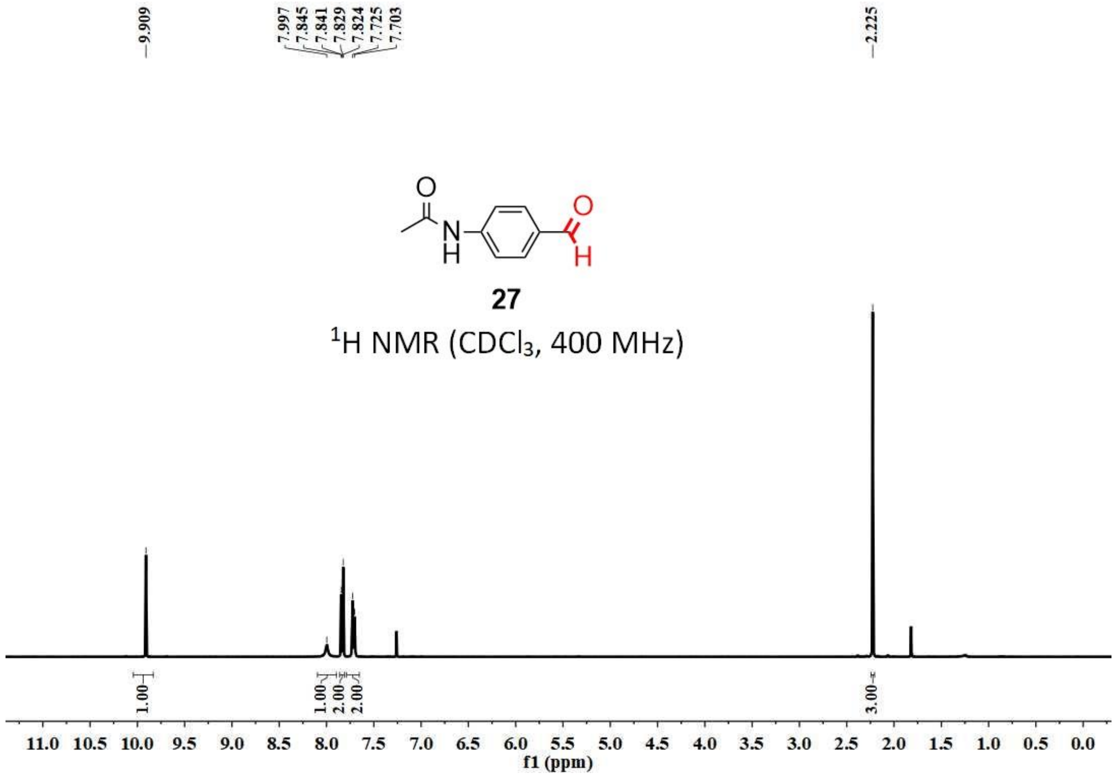
9.909
7.997
7.845
7.841
7.829
7.824
7.725
7.703
2.225
27
¹H NMR (CDCl₃, 400 MHz)
1.00
1.00
2.00
2.00
3.00
11.0 10.5 10.0 9.5 9.0 8.5 8.0 7.5 7.0 6.5 6.0 5.5 5.0 4.5 4.0 3.5 3.0 2.5 2.0 1.5 1.0 0.5 0.0
f1 (ppm)

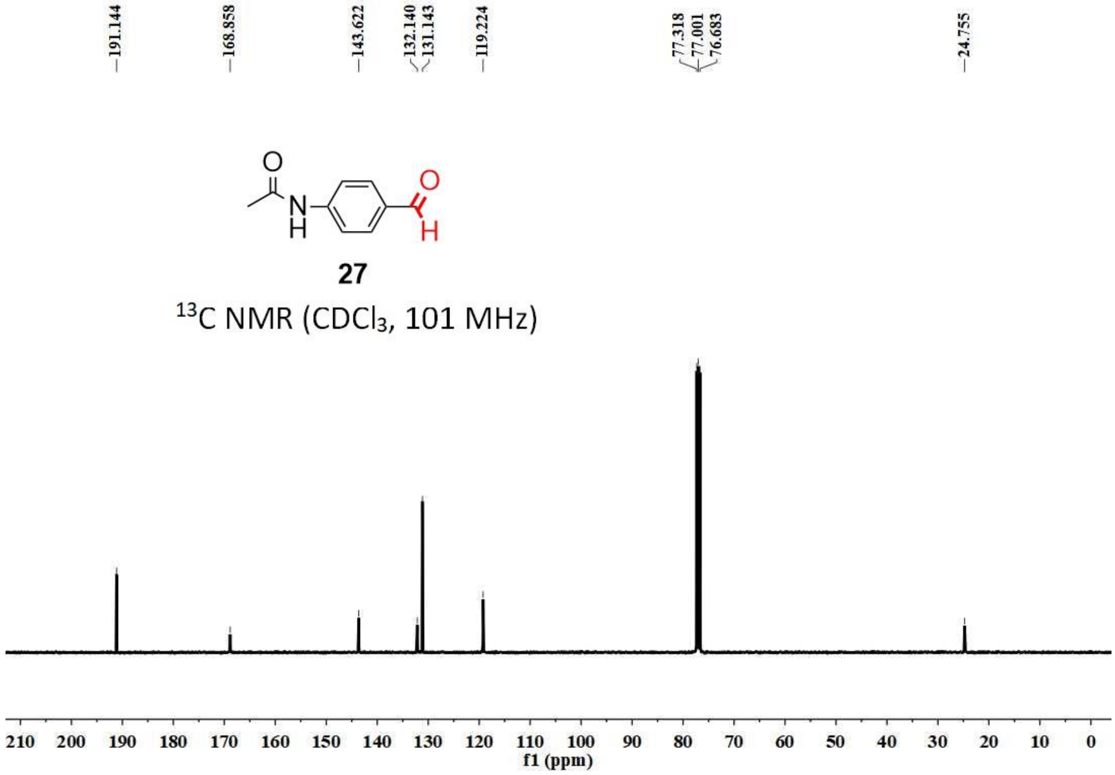
191.144
168.858
143.622
132.140
131.143
119.224
77.318
77.001
76.683
24.755
27
¹³C NMR (CDCl₃, 101 MHz)
210 200 190 180 170 160 150 140 130 120 110 100 90 80 70 60 50 40 30 20 10 0
f1 (ppm)

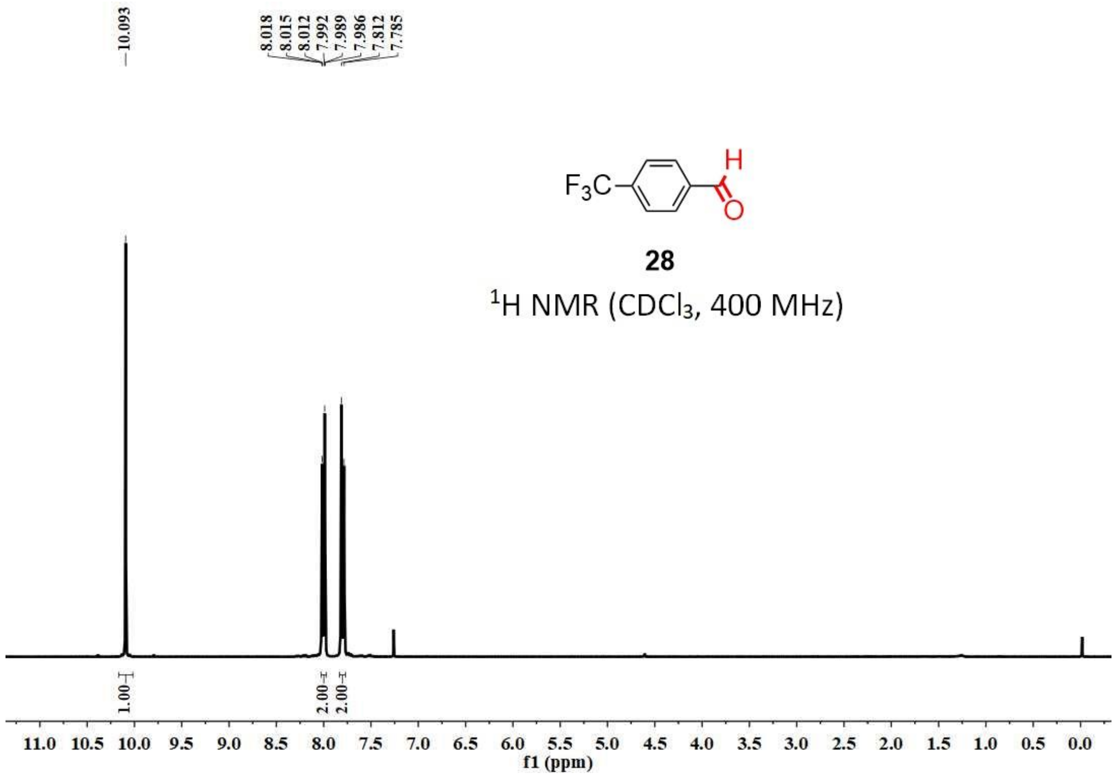
10.093
8.018
8.015
8.012
7.992
7.989
7.986
7.812
7.785
F3C
H
O
28
1H NMR (CDCl3, 400 MHz)
1.00
2.00
2.00
11.0 10.5 10.0 9.5 9.0 8.5 8.0 7.5 7.0 6.5 6.0 5.5 5.0 4.5 4.0 3.5 3.0 2.5 2.0 1.5 1.0 0.5 0.0
f1 (ppm)

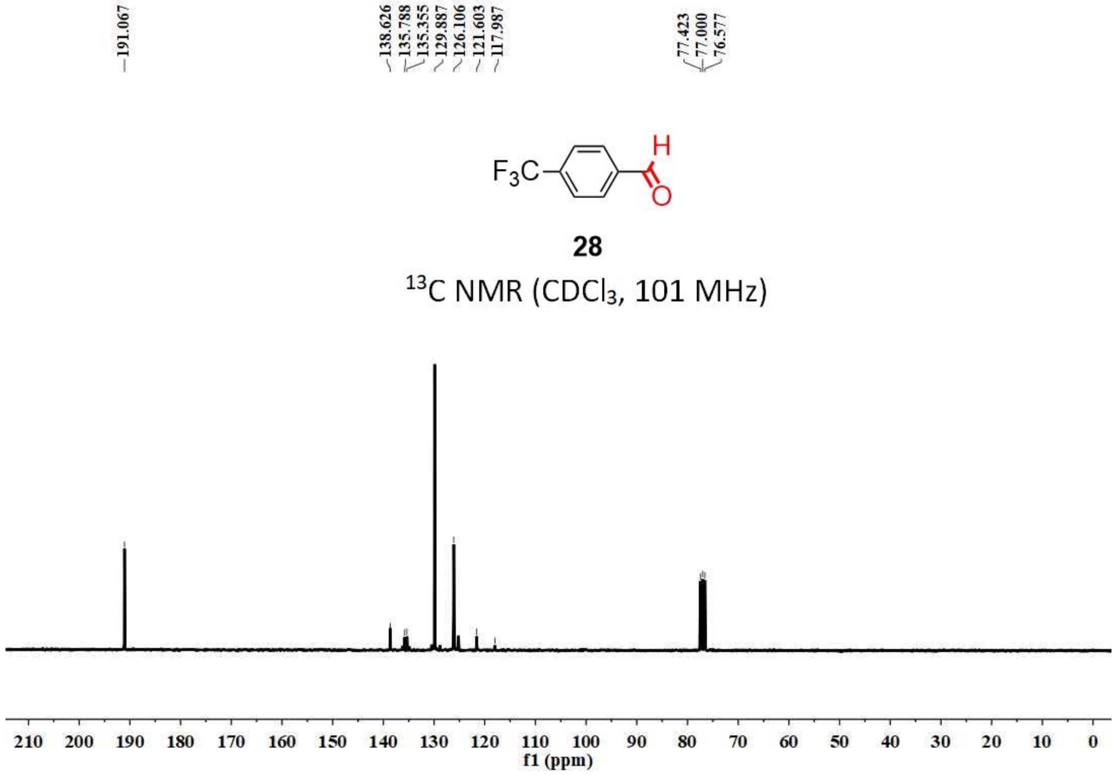
191.067
138.626
135.788
135.355
129.887
126.106
121.603
117.987
77.423
77.000
76.577
F3C
H
O
28
13C NMR (CDCl3, 101 MHz)
210 200 190 180 170 160 150 140 130 120 110 100 90 80 70 60 50 40 30 20 10 0
f1 (ppm)

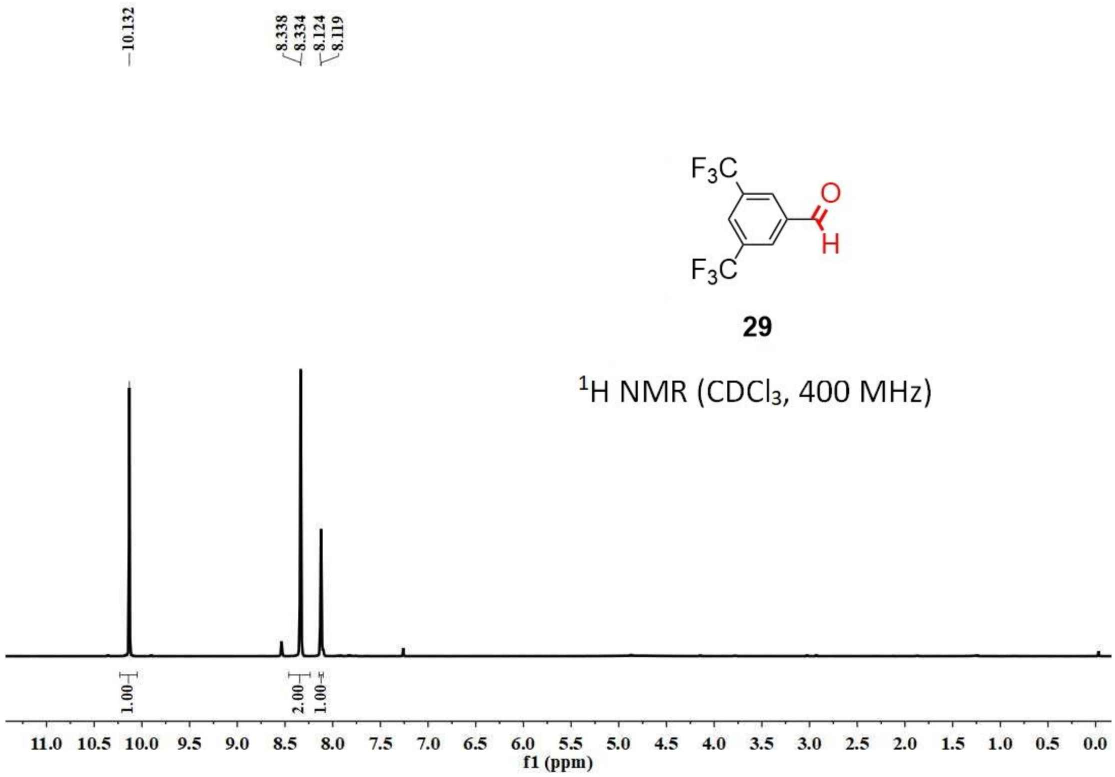
10.132
8.338
8.334
8.124
8.119
F3C
O
H
F3C
29
1H NMR (CDCl3, 400 MHz)
1.00
2.00
1.00
11.0 10.5 10.0 9.5 9.0 8.5 8.0 7.5 7.0 6.5 6.0 5.5 5.0 4.5 4.0 3.5 3.0 2.5 2.0 1.5 1.0 0.5 0.0
f1 (ppm)

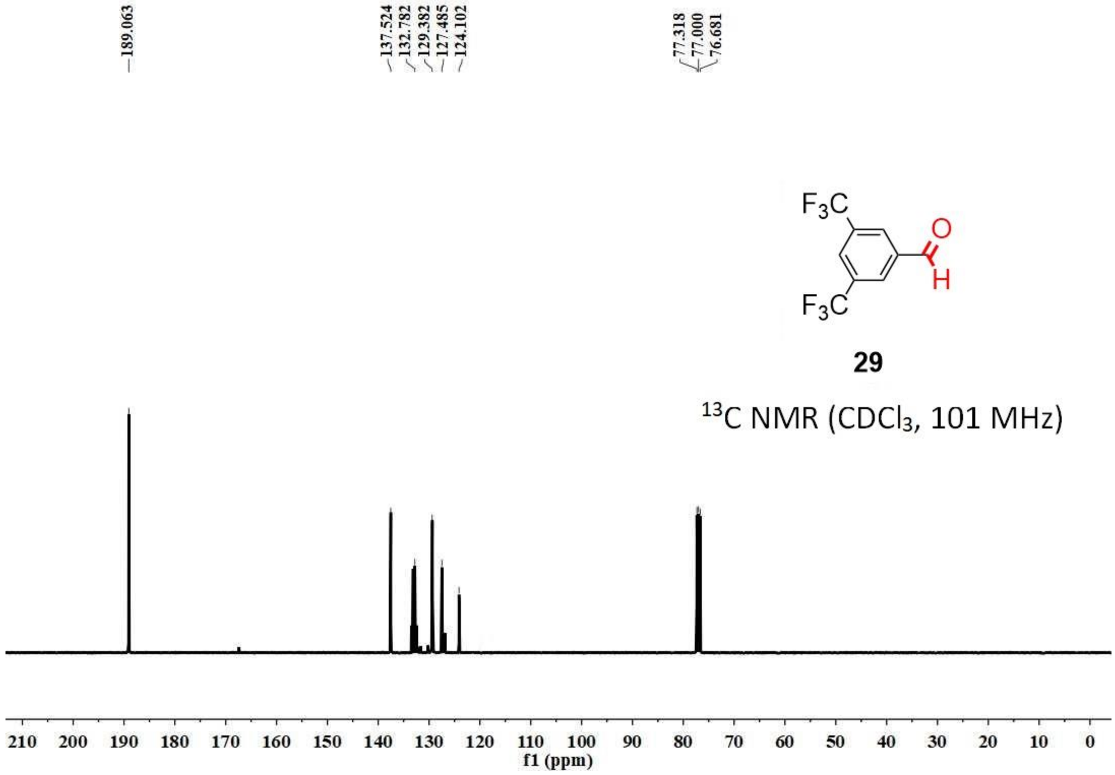
189.063
137.524
132.782
129.382
127.485
124.102
77.318
77.000
76.681
F3C
O
H
F3C
29
13C NMR (CDCl3, 101 MHz)
210 200 190 180 170 160 150 140 130 120 110 100 90 80 70 60 50 40 30 20 10 0
f1 (ppm)

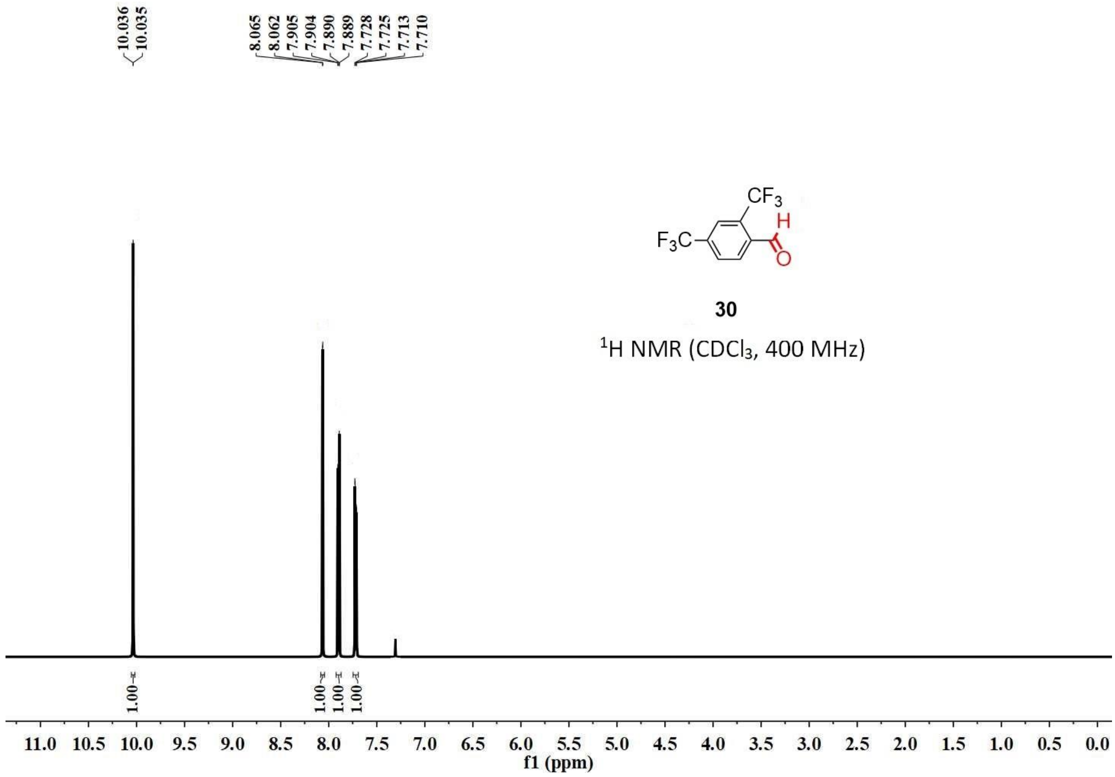
10.036
10.035
8.065
8.062
7.905
7.904
7.890
7.889
7.728
7.725
7.713
7.710
CF_3
H
F_3C
O
30
^{1}H NMR ($CDCl_3$, 400 MHz)
1.00
1.00
1.00
1.00
11.0 10.5 10.0 9.5 9.0 8.5 8.0 7.5 7.0 6.5 6.0 5.5 5.0 4.5 4.0 3.5 3.0 2.5 2.0 1.5 1.0 0.5 0.0
f1 (ppm)

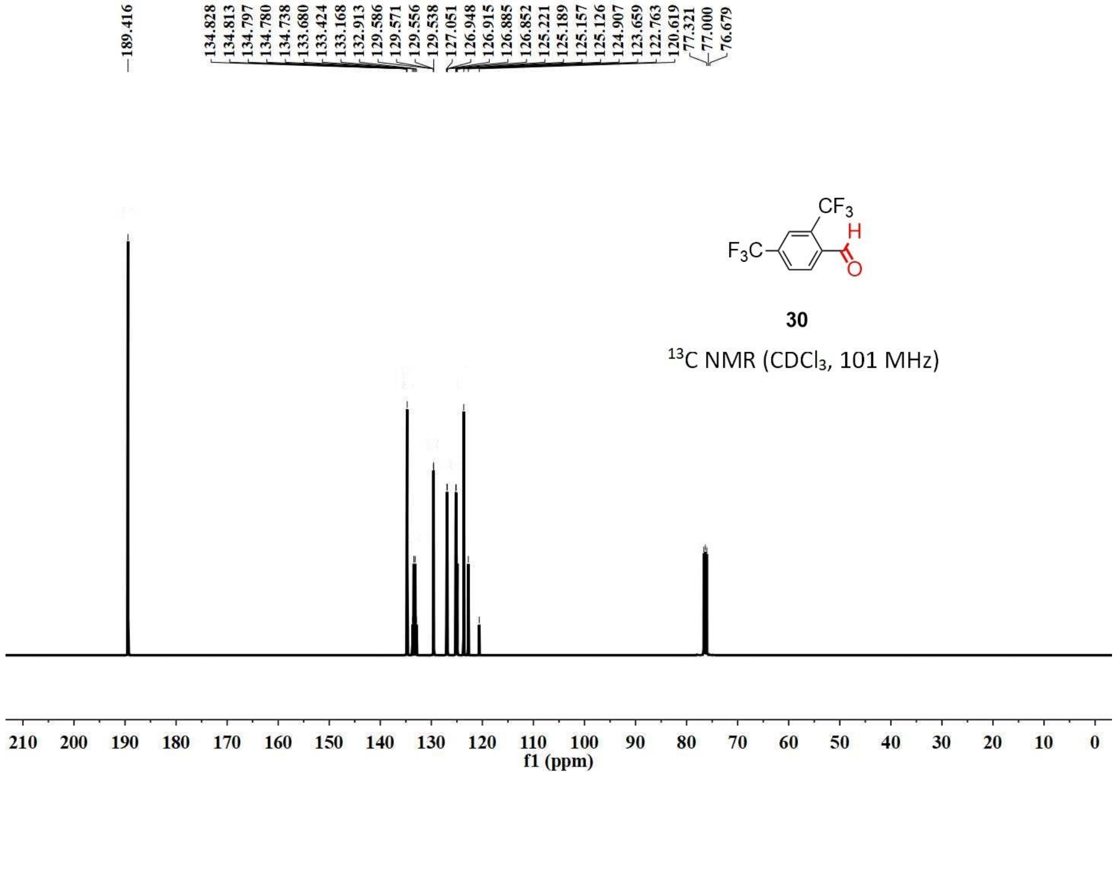
189.416
134.828
134.813
134.797
134.780
134.738
133.680
133.424
133.168
132.913
129.586
129.571
129.556
129.538
127.051
126.948
126.915
126.885
126.852
125.221
125.189
125.157
125.126
124.907
123.659
122.763
120.619
77.321
77.000
76.679
CF_3
H
F_3C
O
30
^{13}C NMR ($CDCl_3$, 101 MHz)
210 200 190 180 170 160 150 140 130 120 110 100 90 80 70 60 50 40 30 20 10 0
f1 (ppm)

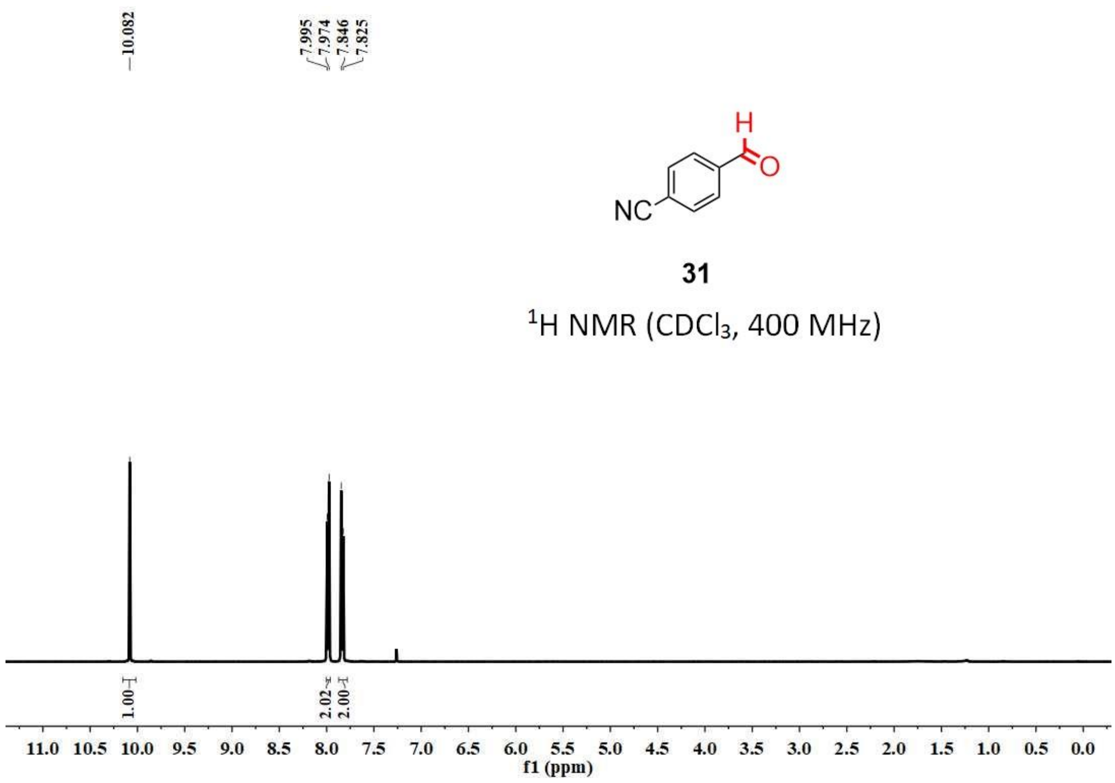

31

^{1}H NMR ($CDCl_3$, 400 MHz)

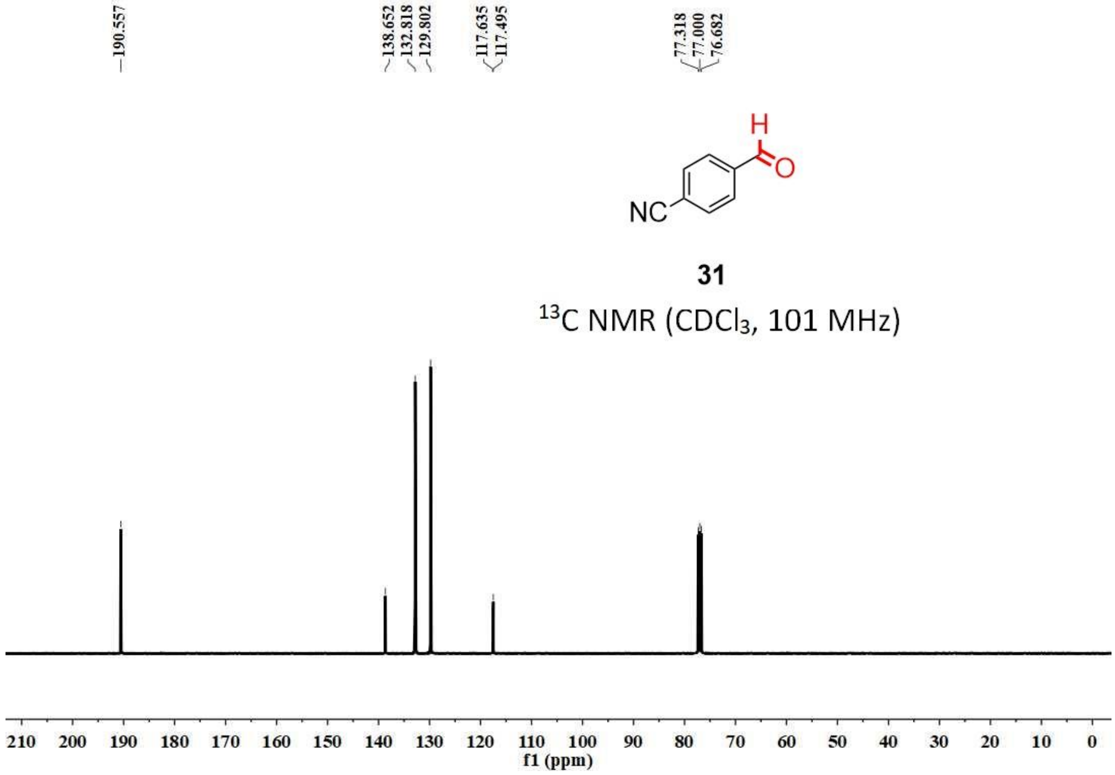

31

^{13}C NMR ($CDCl_3$, 101 MHz)

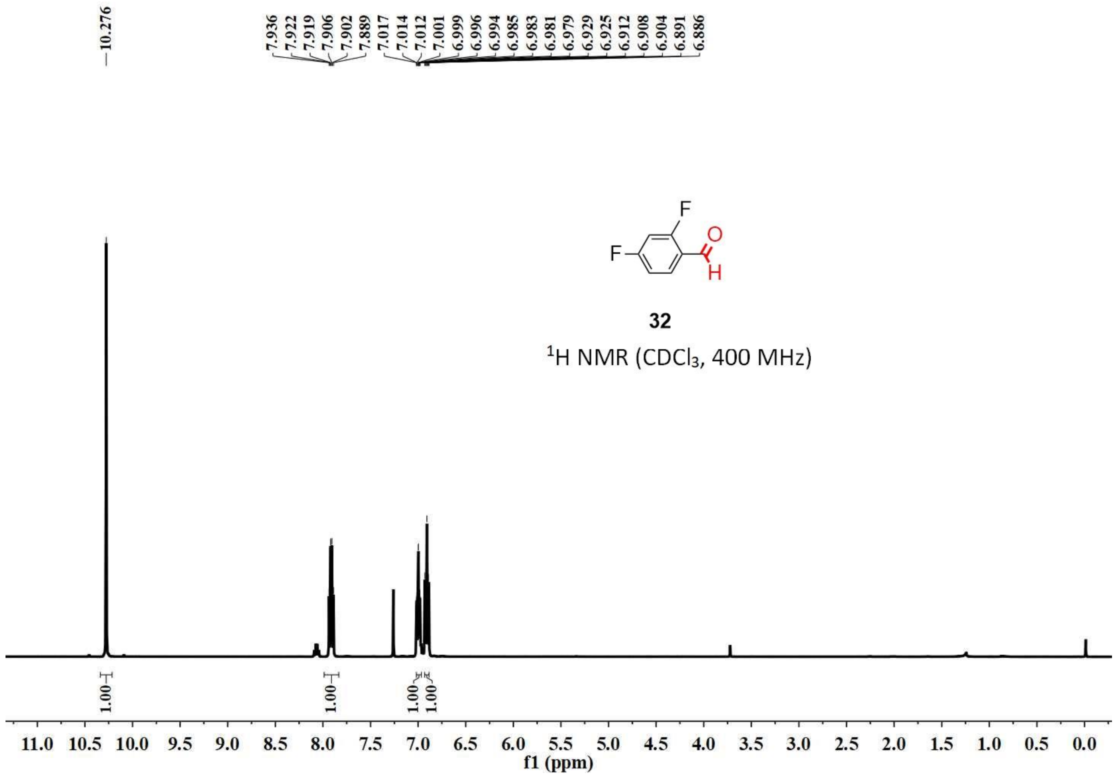
10.276
7.936 7.922 7.919 7.906 7.902 7.889 7.017 7.014 7.012 7.001 6.999 6.996 6.994 6.985 6.983 6.981 6.979 6.929 6.925 6.912 6.908 6.904 6.891 6.886
F
F
O
H
32
1H NMR (CDCl3, 400 MHz)
1.00
1.00
1.00
1.00
11.0 10.5 10.0 9.5 9.0 8.5 8.0 7.5 7.0 6.5 6.0 5.5 5.0 4.5 4.0 3.5 3.0 2.5 2.0 1.5 1.0 0.5 0.0
f1 (ppm)

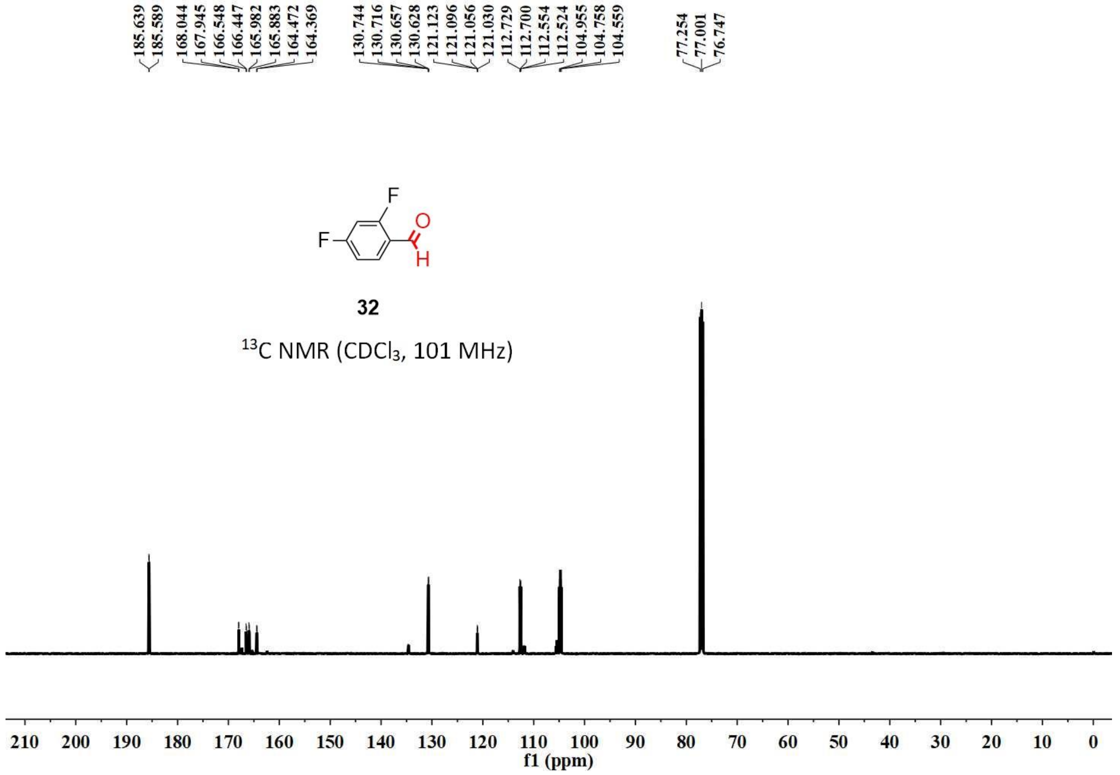
185.639 185.589 168.044 167.945 166.548 166.447 165.982 165.883 164.472 164.369
130.744 130.716 130.657 130.628 121.123 121.096 121.056 121.030 112.729 112.700 112.554 112.524 104.955 104.758 104.559
77.254 77.001 76.747
F
F
O
H
32
13C NMR (CDCl3, 101 MHz)
210 200 190 180 170 160 150 140 130 120 110 100 90 80 70 60 50 40 30 20 10 0
f1 (ppm)

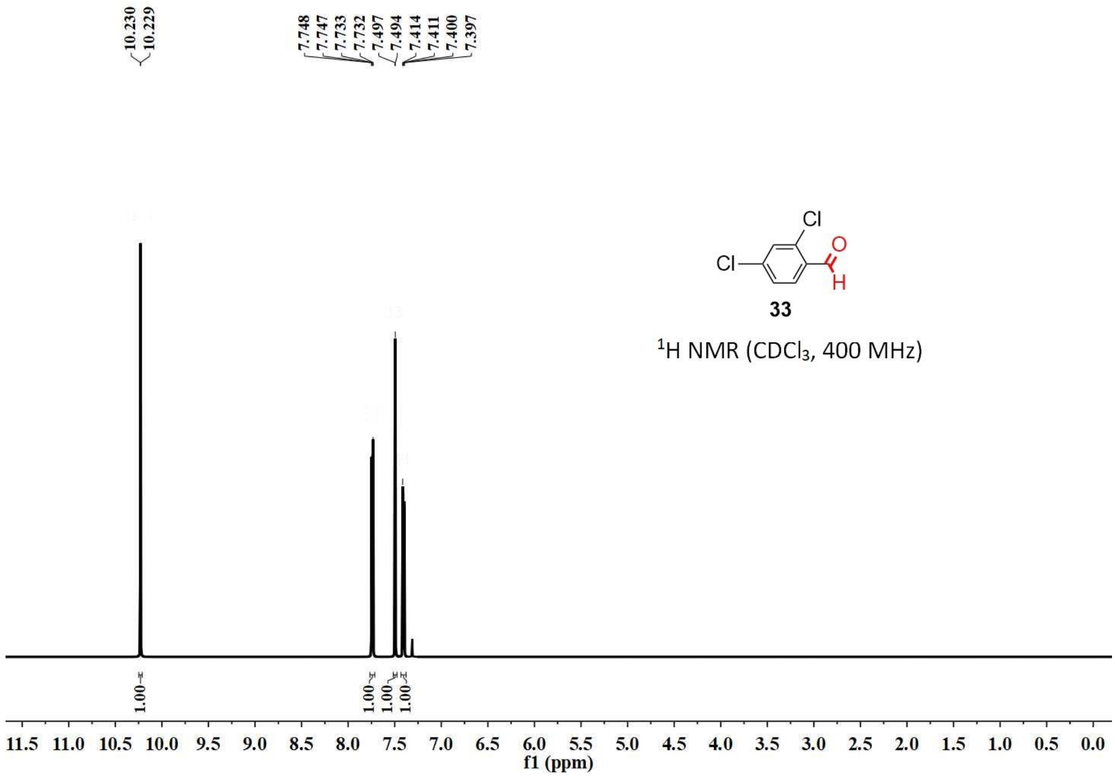
10.230
10.229
7.748
7.747
7.733
7.732
7.497
7.494
7.414
7.411
7.400
7.397
Cl
Cl
O
H
33
^{1}H NMR ($CDCl_3$, 400 MHz)
1.00
1.00
1.00
1.00
11.5 11.0 10.5 10.0 9.5 9.0 8.5 8.0 7.5 7.0 6.5 6.0 5.5 5.0 4.5 4.0 3.5 3.0 2.5 2.0 1.5 1.0 0.5 0.0
f1 (ppm)

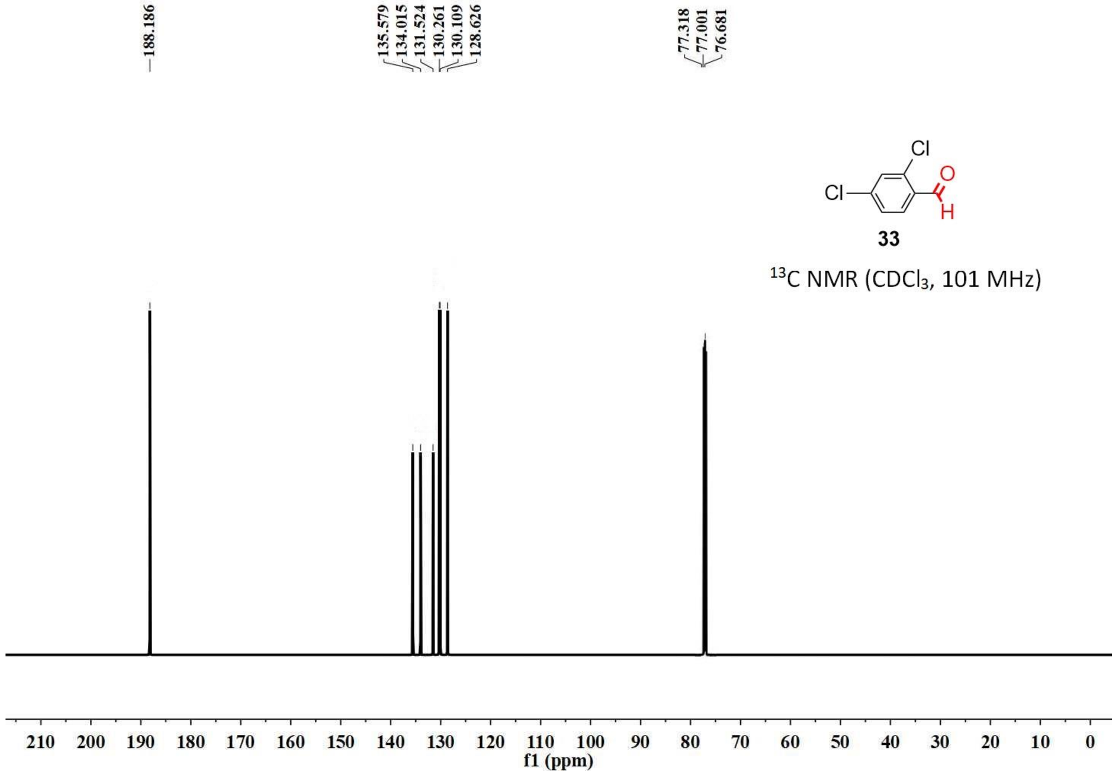
188.186
135.579
134.015
131.524
130.261
130.109
128.626
77.318
77.001
76.681
Cl
Cl
O
H
33
^{13}C NMR ($CDCl_3$, 101 MHz)
210 200 190 180 170 160 150 140 130 120 110 100 90 80 70 60 50 40 30 20 10 0
f1 (ppm)

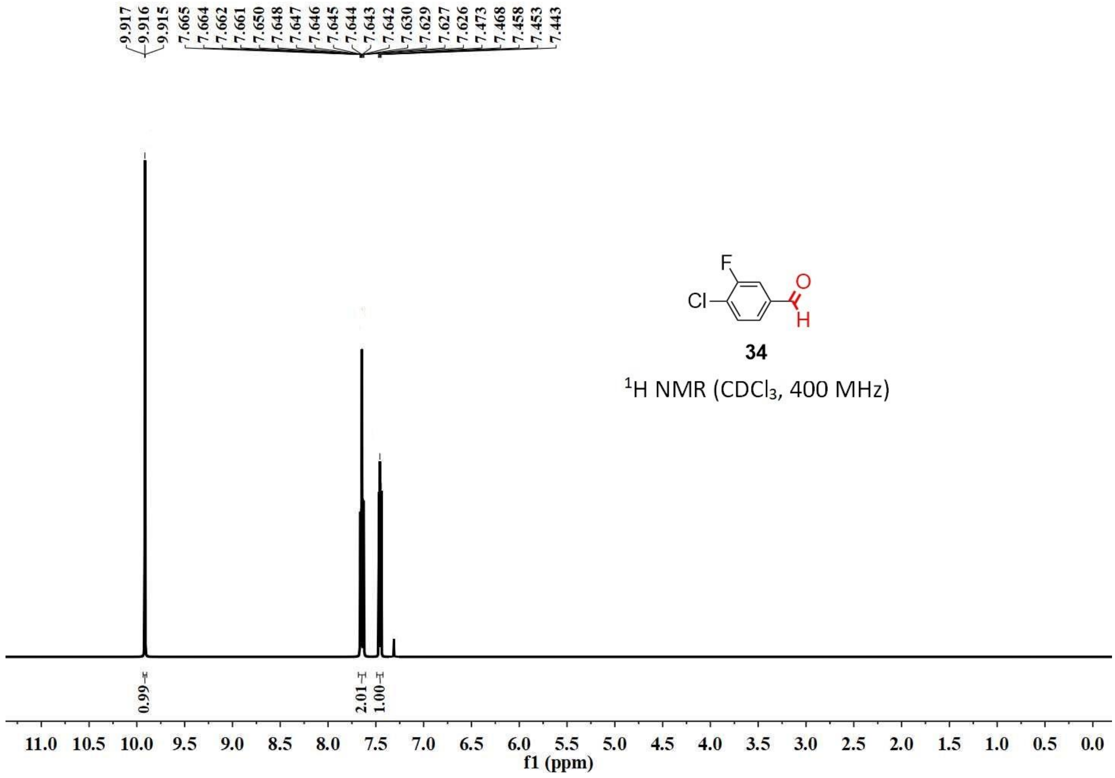
9.917
9.916
9.915
7.665
7.664
7.662
7.661
7.650
7.648
7.647
7.646
7.645
7.644
7.643
7.642
7.630
7.629
7.627
7.626
7.473
7.468
7.458
7.453
7.443
F
Cl
O
H
34
1H NMR (CDCl3, 400 MHz)
0.99
2.01
1.00
11.0 10.5 10.0 9.5 9.0 8.5 8.0 7.5 7.0 6.5 6.0 5.5 5.0 4.5 4.0 3.5 3.0 2.5 2.0 1.5 1.0 0.5 0.0
f1 (ppm)

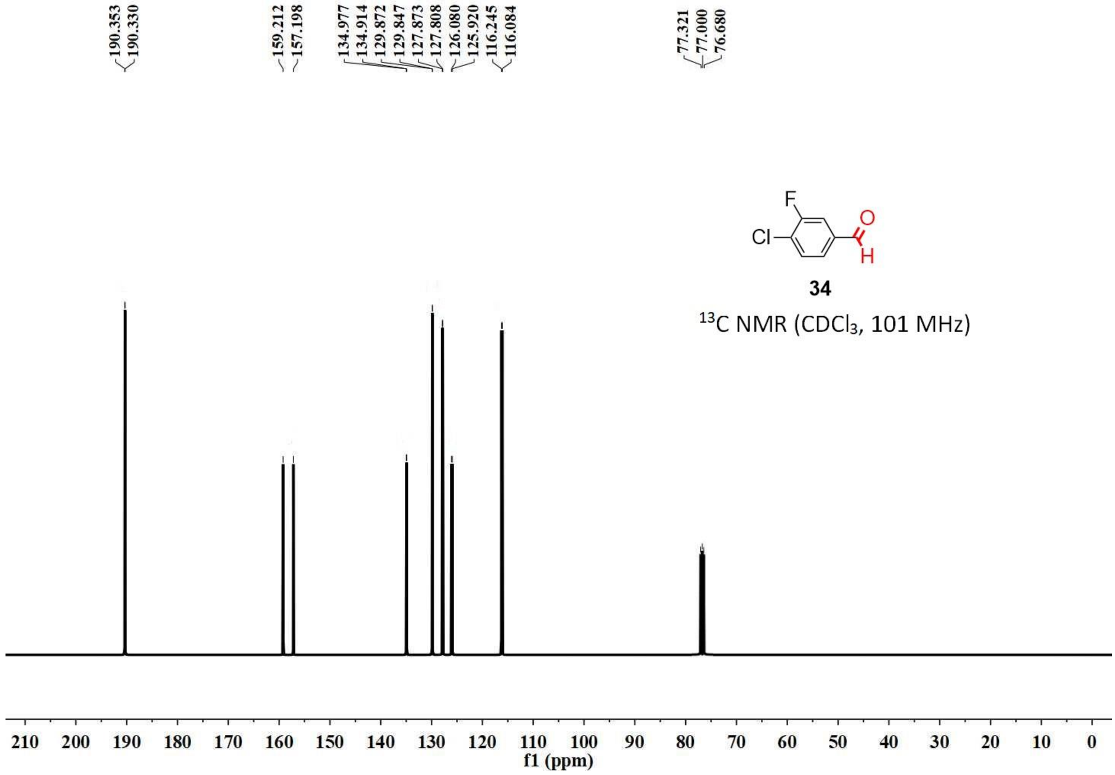
190.353
190.330
159.212
157.198
134.977
134.914
129.872
129.847
127.873
127.808
126.080
125.920
116.245
116.084
77.321
77.000
76.680
F
Cl
O
H
34
13C NMR (CDCl3, 101 MHz)
210 200 190 180 170 160 150 140 130 120 110 100 90 80 70 60 50 40 30 20 10 0
f1 (ppm)

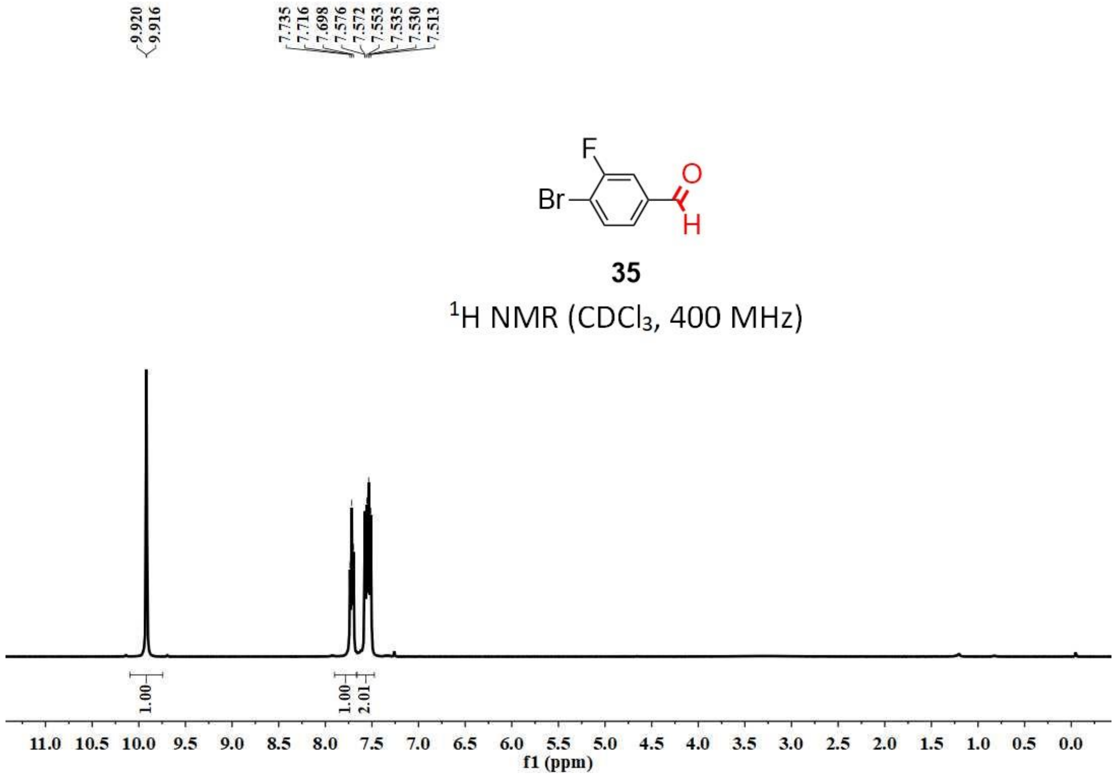
9.920
9.916
7.735
7.716
7.698
7.576
7.572
7.553
7.535
7.530
7.513
F
Br
O
H
35
^{1}H NMR (CDCl$_3$, 400 MHz)
1.00
1.00
2.01
11.0 10.5 10.0 9.5 9.0 8.5 8.0 7.5 7.0 6.5 6.0 5.5 5.0 4.5 4.0 3.5 3.0 2.5 2.0 1.5 1.0 0.5 0.0
f1 (ppm)

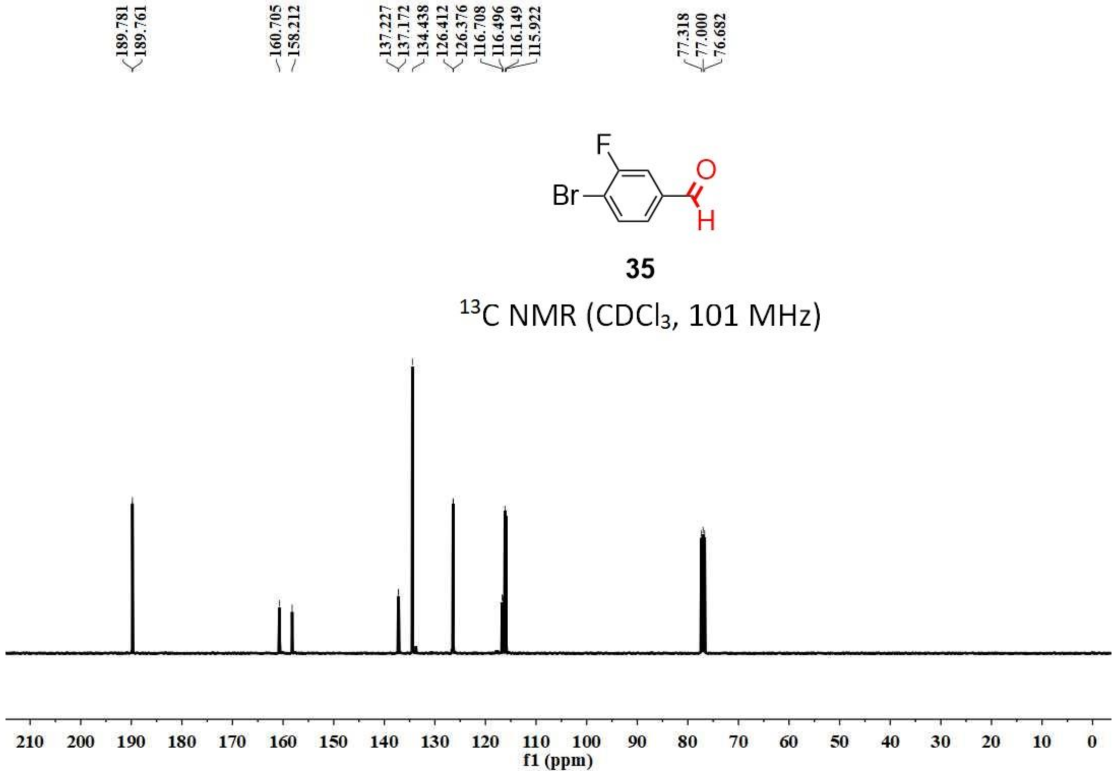
189.781
189.761
160.705
158.212
137.227
137.172
134.438
126.412
126.376
116.708
116.496
116.149
115.922
77.318
77.000
76.682
F
Br
O
H
35
^{13}C NMR (CDCl$_3$, 101 MHz)
210 200 190 180 170 160 150 140 130 120 110 100 90 80 70 60 50 40 30 20 10 0
f1 (ppm)

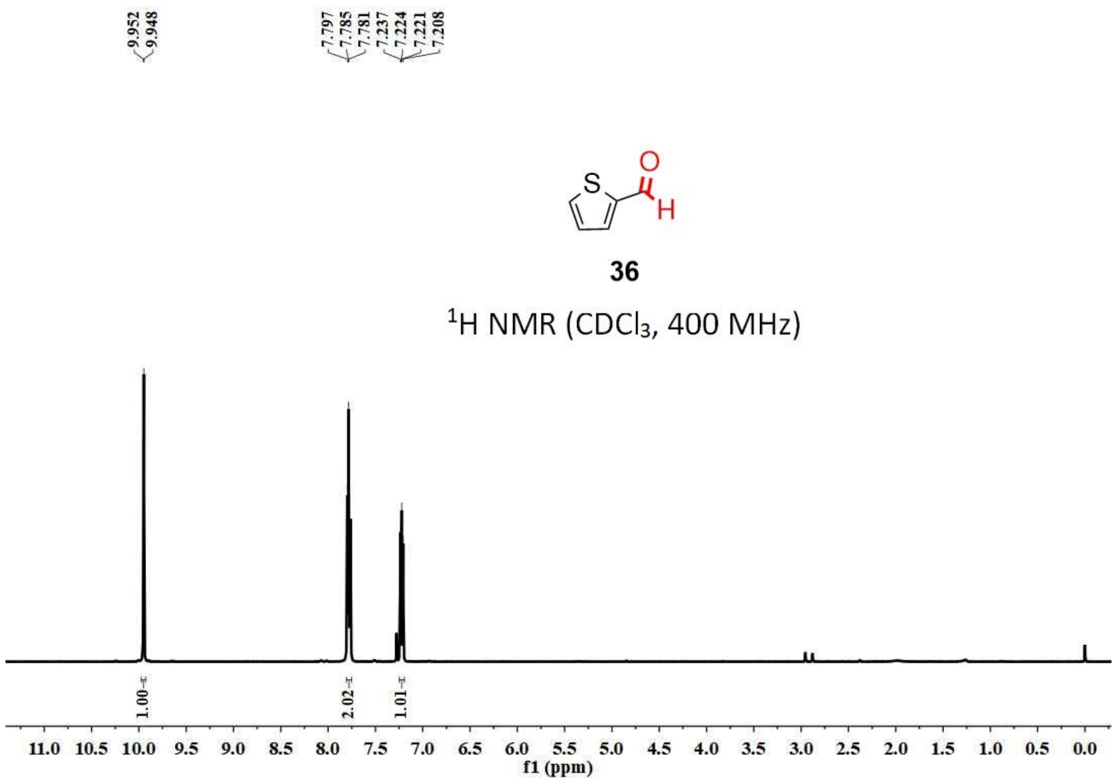
9.952
9.948
7.797
7.785
7.781
7.237
7.224
7.221
7.208
S
O
H
36
1H NMR (CDCl3, 400 MHz)
1.00
2.02
1.01
11.0 10.5 10.0 9.5 9.0 8.5 8.0 7.5 7.0 6.5 6.0 5.5 5.0 4.5 4.0 3.5 3.0 2.5 2.0 1.5 1.0 0.5 0.0
f1 (ppm)

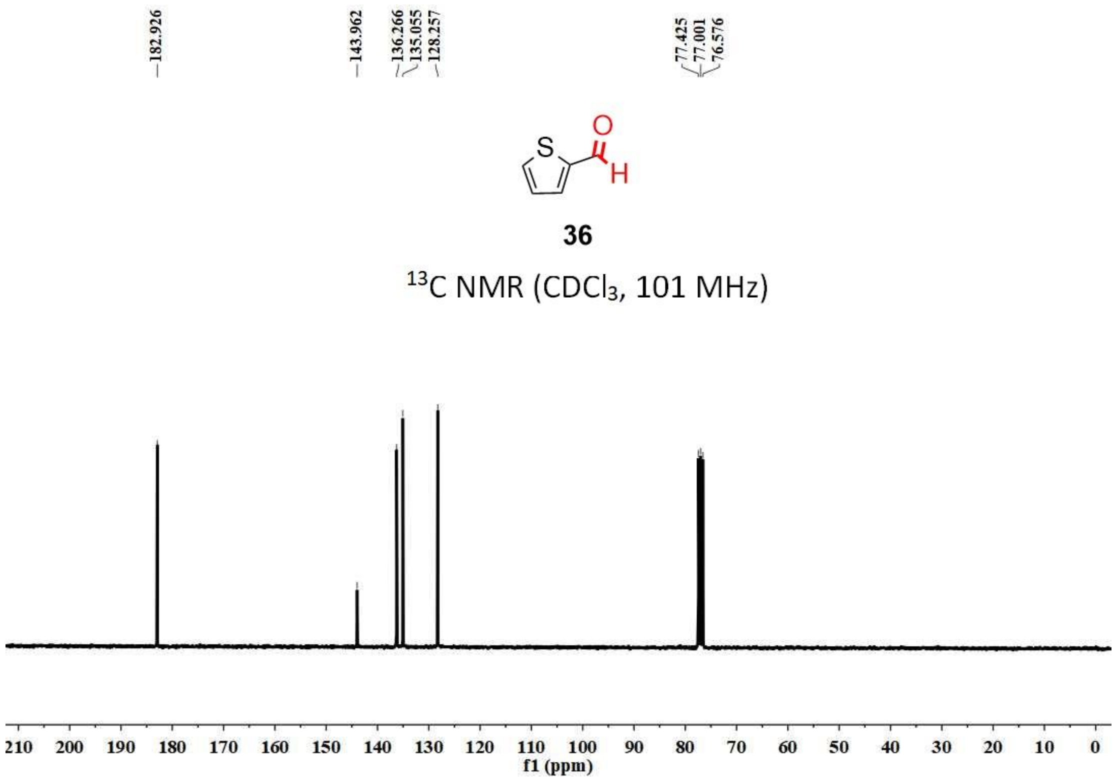
182.926
143.962
136.266
135.055
128.257
77.425
77.001
76.576
S
O
H
36
13C NMR (CDCl3, 101 MHz)
210 200 190 180 170 160 150 140 130 120 110 100 90 80 70 60 50 40 30 20 10 0
f1 (ppm)

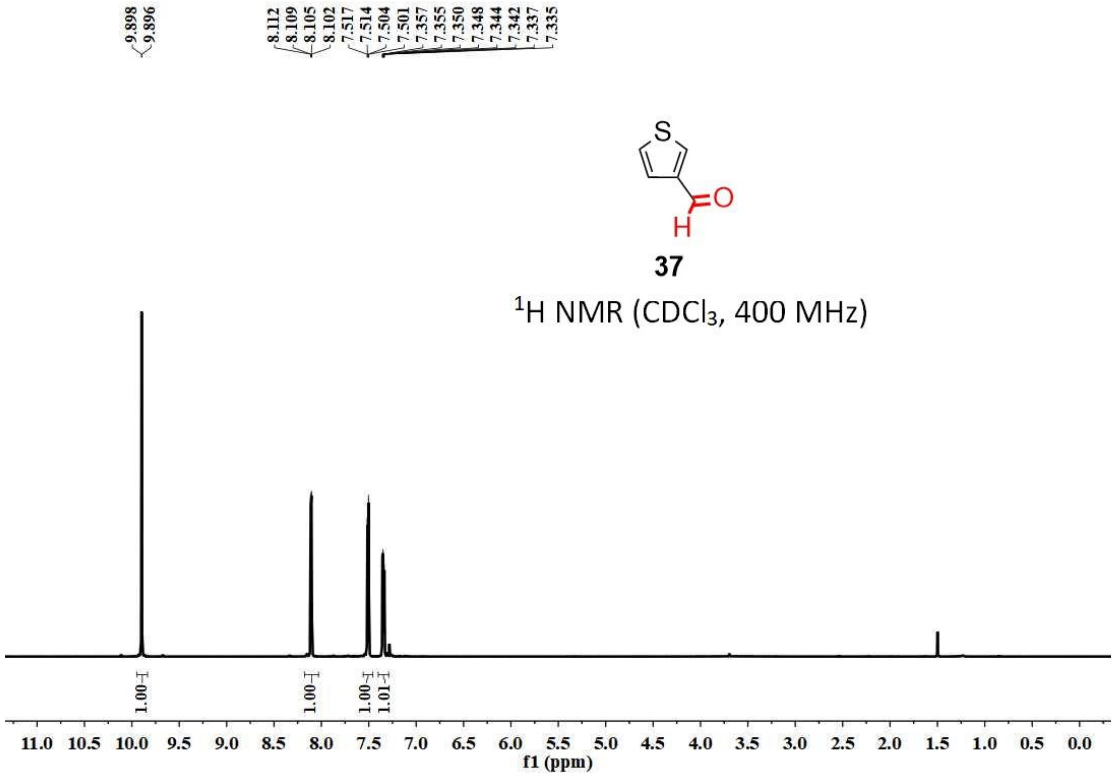

37

^1H NMR ($CDCl_3$, 400 MHz)

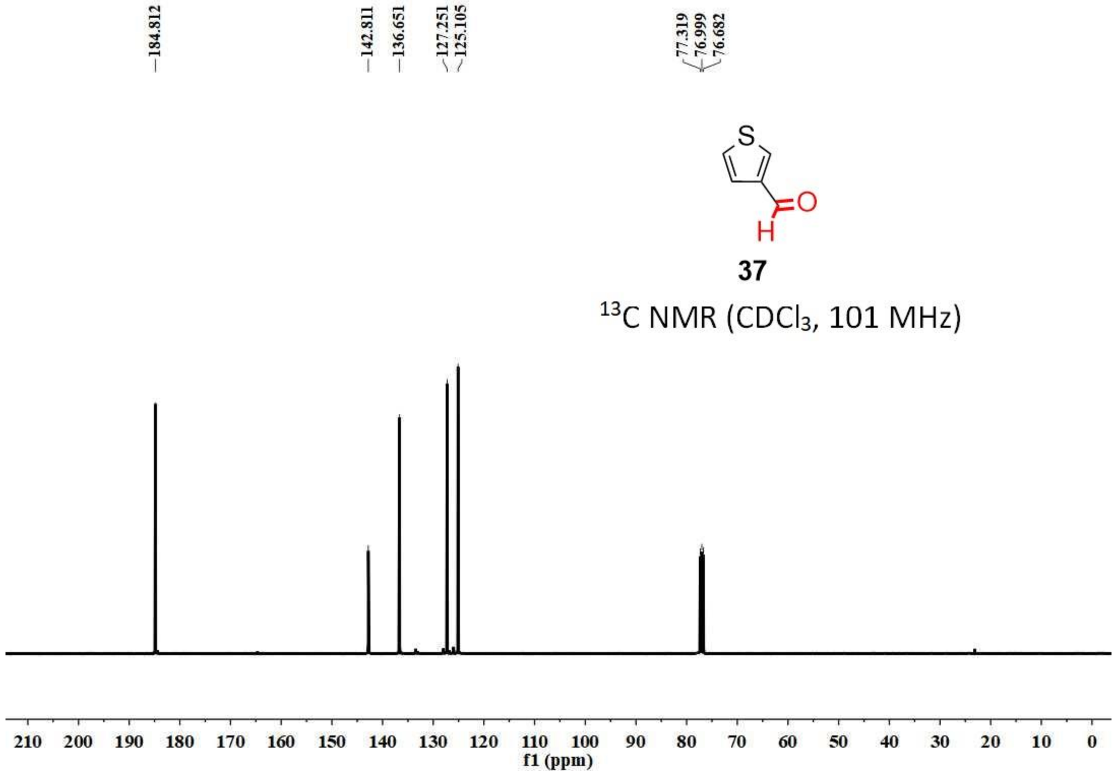

37

^{13}C NMR ($CDCl_3$, 101 MHz)

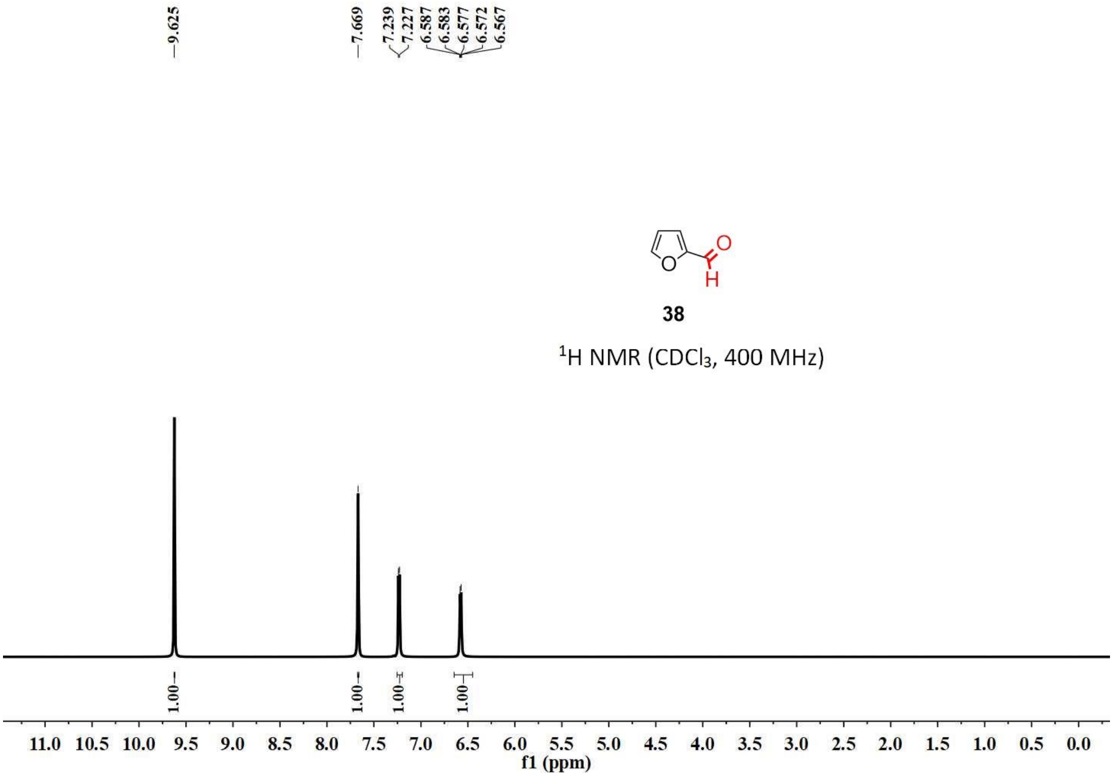

38

^{1}H NMR ($CDCl_3$, 400 MHz)

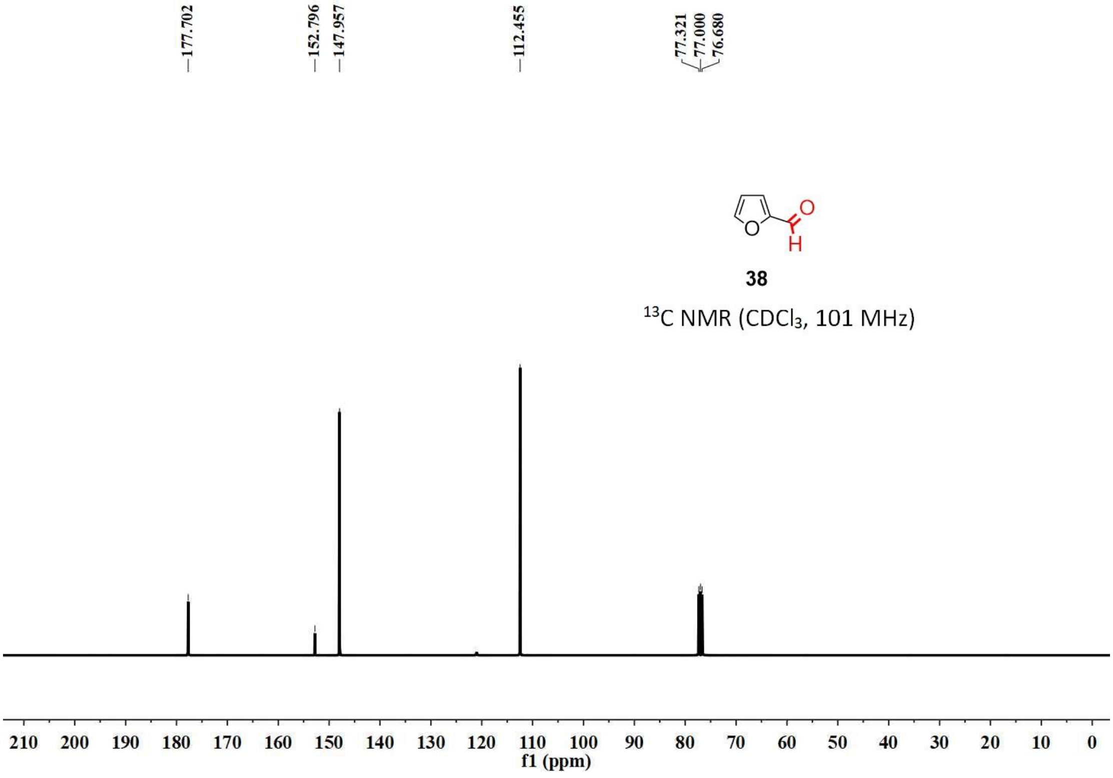

38

^{13}C NMR ($CDCl_3$, 101 MHz)

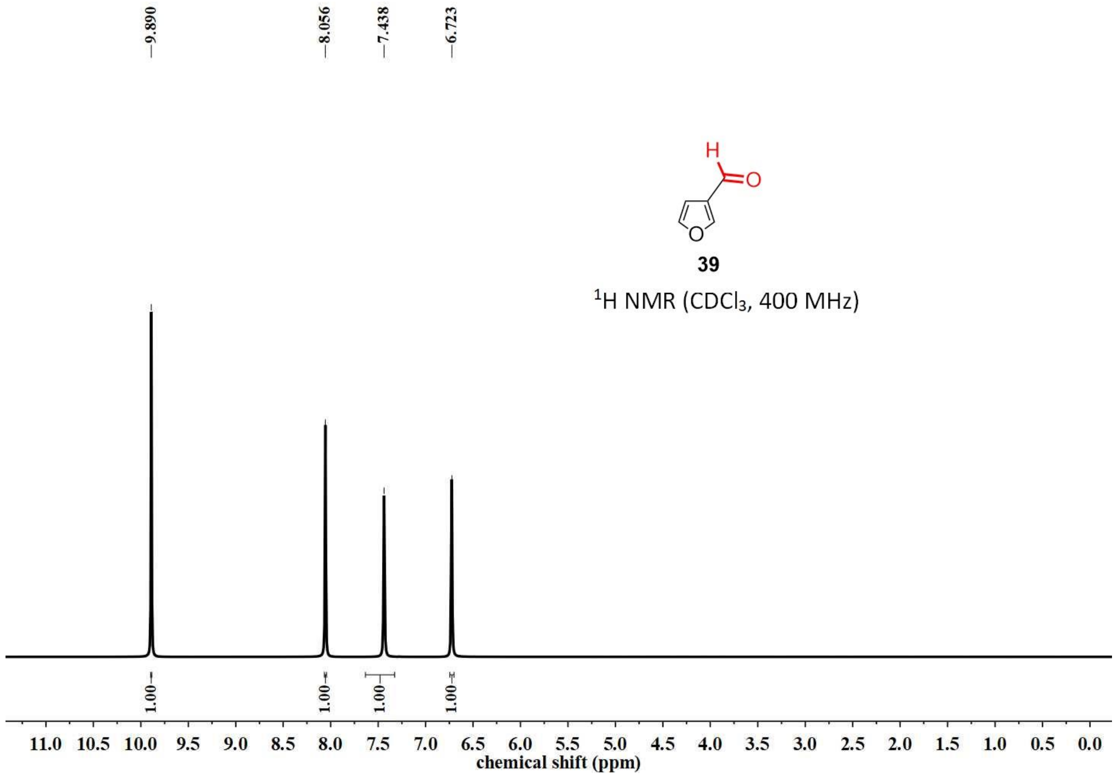

9.890
8.056
7.438
6.723
H
O
O
39
^{1}H NMR ($CDCl_3$, 400 MHz)
1.00
1.00
1.00
1.00
11.0 10.5 10.0 9.5 9.0 8.5 8.0 7.5 7.0 6.5 6.0 5.5 5.0 4.5 4.0 3.5 3.0 2.5 2.0 1.5 1.0 0.5 0.0
chemical shift (ppm)

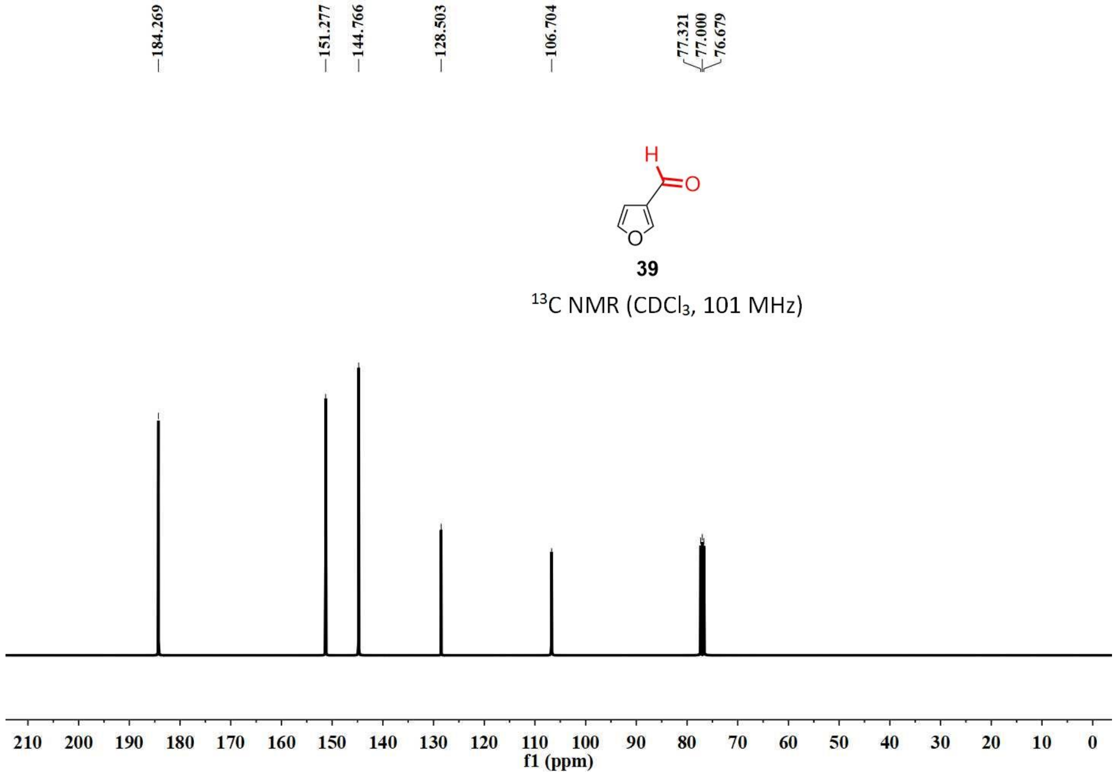

184.269
151.277
144.766
128.503
106.704
77.321
77.000
76.679
H
O
O
39
^{13}C NMR ($CDCl_3$, 101 MHz)
210 200 190 180 170 160 150 140 130 120 110 100 90 80 70 60 50 40 30 20 10 0
f1 (ppm)

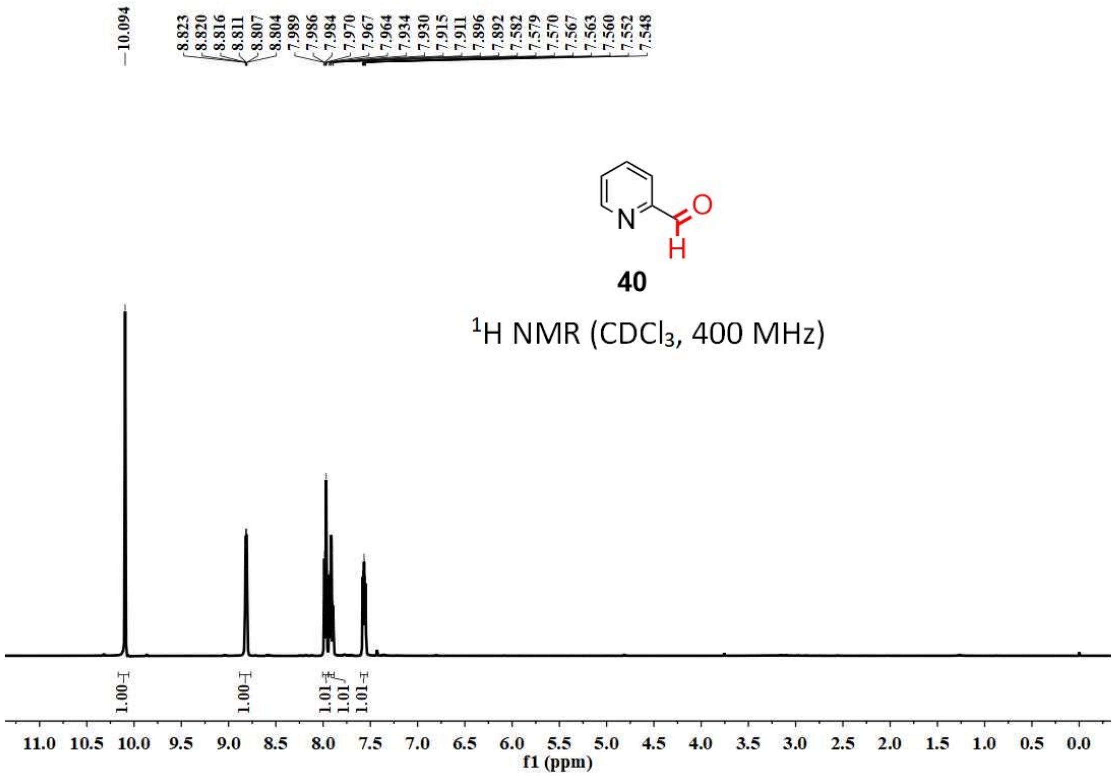
10.094
8.823
8.820
8.816
8.811
8.807
8.804
7.989
7.986
7.984
7.970
7.967
7.964
7.934
7.930
7.915
7.911
7.896
7.892
7.582
7.579
7.570
7.567
7.563
7.560
7.552
7.548
N
O
H
40
1H NMR (CDCl3, 400 MHz)
1.00
1.00
1.01
1.01
1.01
11.0 10.5 10.0 9.5 9.0 8.5 8.0 7.5 7.0 6.5 6.0 5.5 5.0 4.5 4.0 3.5 3.0 2.5 2.0 1.5 1.0 0.5 0.0
f1 (ppm)

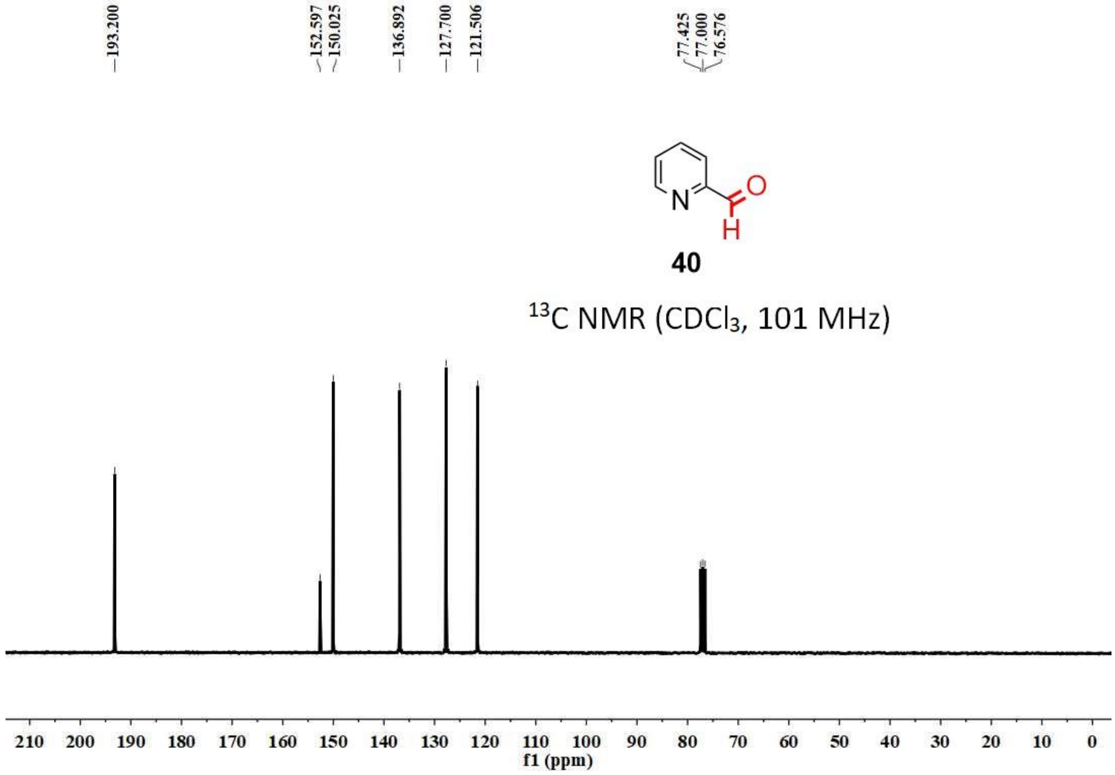
193.200
152.597
150.025
136.892
127.700
121.506
77.425
77.000
76.576
N
O
H
40
13C NMR (CDCl3, 101 MHz)
210 200 190 180 170 160 150 140 130 120 110 100 90 80 70 60 50 40 30 20 10 0
f1 (ppm)

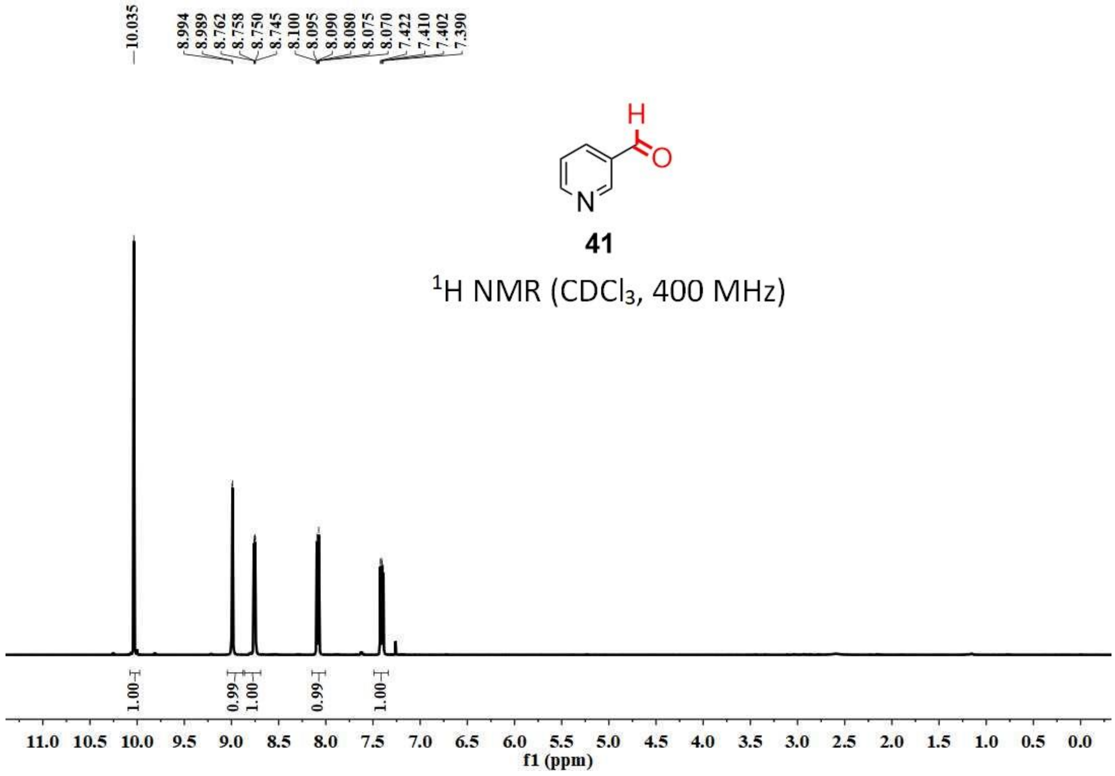
41
1H NMR (CDCl3, 400 MHz)
10.035
8.994
8.989
8.762
8.758
8.750
8.745
8.100
8.095
8.090
8.080
8.075
8.070
7.422
7.410
7.402
7.390
1.00
0.99
1.00
0.99
1.00
f1 (ppm)

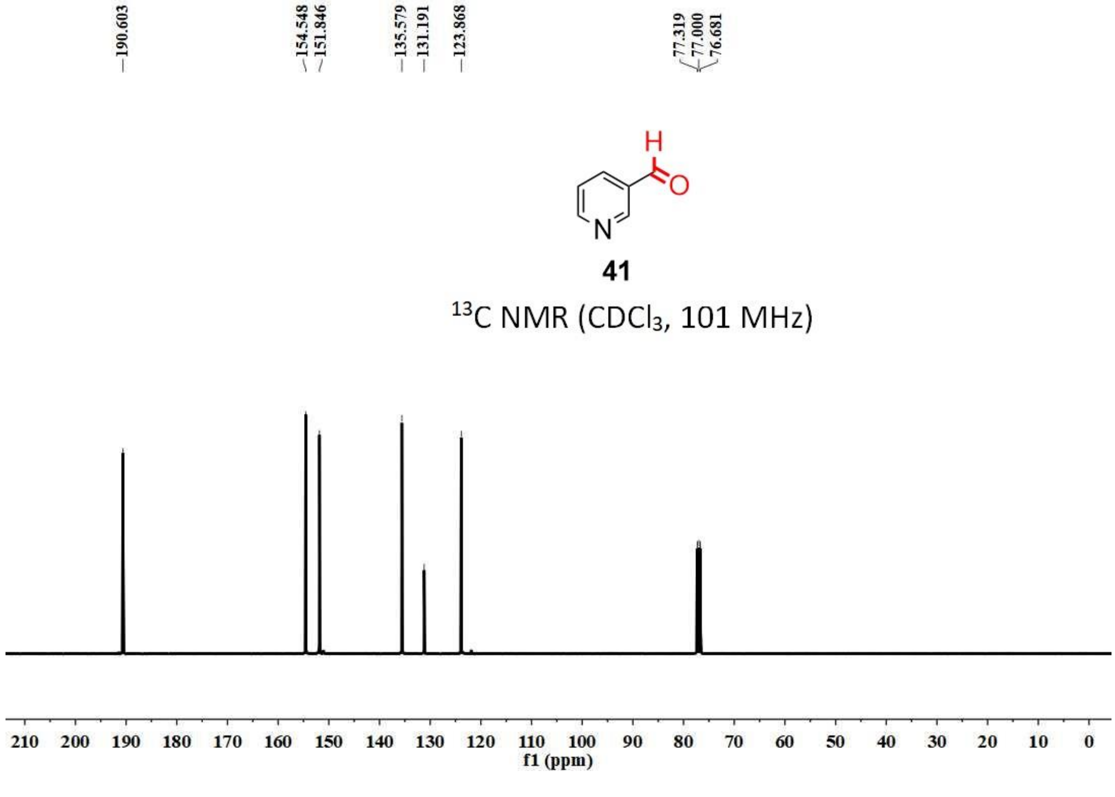
41
13C NMR (CDCl3, 101 MHz)
190.603
154.548
151.846
135.579
131.191
123.868
77.319
77.000
76.681
f1 (ppm)

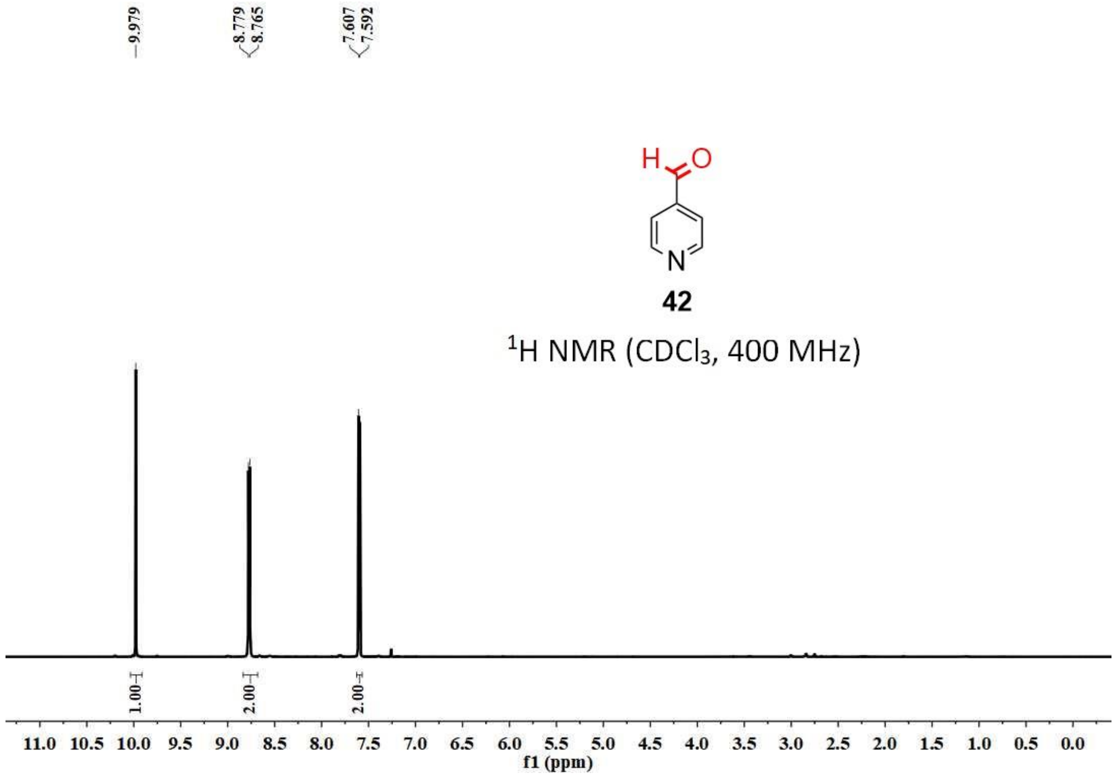
9.979
8.779
8.765
7.607
7.592
H
O
N
42
^{1}H NMR (CDCl$_3$, 400 MHz)
1.00
2.00
2.00
11.0 10.5 10.0 9.5 9.0 8.5 8.0 7.5 7.0 6.5 6.0 5.5 5.0 4.5 4.0 3.5 3.0 2.5 2.0 1.5 1.0 0.5 0.0
f1 (ppm)

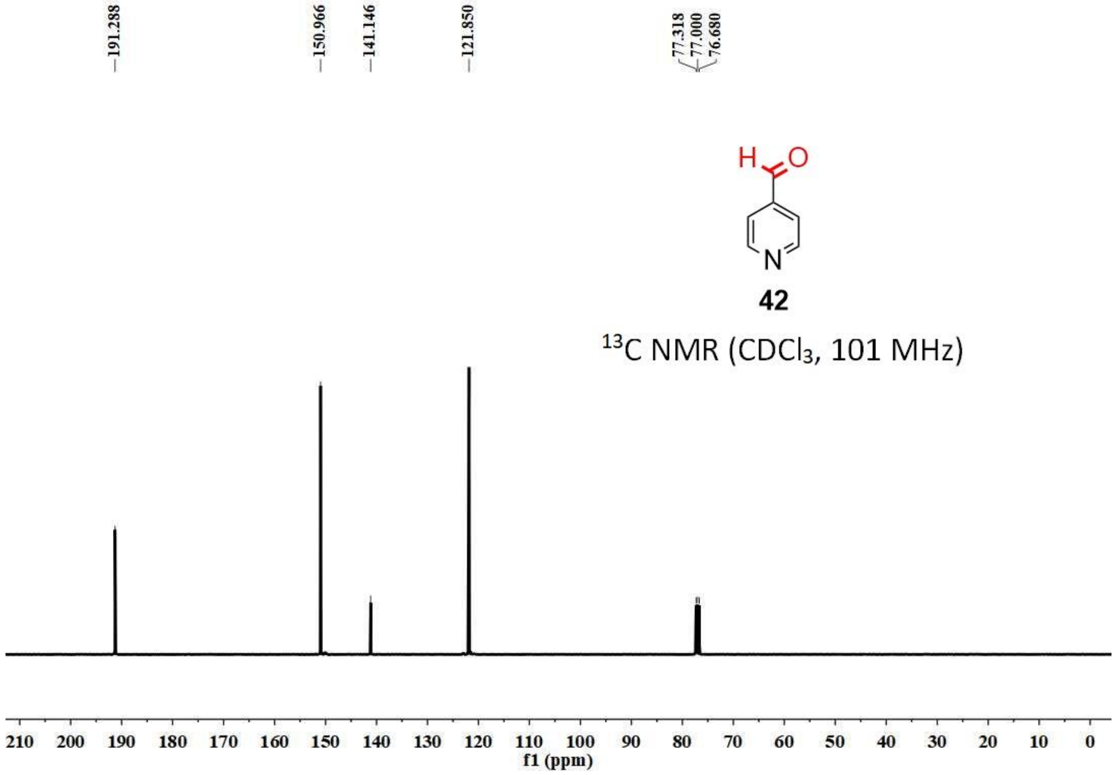
191.288
150.966
141.146
121.850
77.318
77.000
76.680
H
O
N
42
^{13}C NMR (CDCl$_3$, 101 MHz)
210 200 190 180 170 160 150 140 130 120 110 100 90 80 70 60 50 40 30 20 10 0
f1 (ppm)

4. References

[1] K. Natte, A. Dumrath, H. Neumann, M. Beller, Palladium-Catalyzed Carbonylations of Aryl Bromides using Paraformaldehyde: Synthesis of Aldehydes and Esters, Angewandte Chemie-International Edition 53 (2014) 10090-10094.

[2] M.L. Zhang, N. Li, X.Y. Tao, R. Ruzi, S.Y. Yu, C.J. Zhu, Selective reduction of carboxylic acids to aldehydes with hydrosilane via photoredox catalysis, Chemical Communications 53 (2017) 10228-10231.

[3] L.W. Zhan, L. Han, P. Xing, B. Jiang, Copper N-Heterocyclic Carbene: A Catalyst for Aerobic Oxidation or Reduction Reactions, Organic Letters 17 (2015) 5990-5993.

[4] A. Gonzalez-de-Castro, J.L. Xiao, Green and Efficient: Iron-Catalyzed Selective Oxidation of Olefins to Carbonyls with O-2, Journal of the American Chemical Society 137 (2015) 8206-8218.

[5] R. Haraguchi, S. Tanazawa, N. Tokunaga, S. Fukuzawa, Palladium-Catalyzed Formylation of Arylzinc Reagents with S-Phenyl Thioformate, Organic Letters 19 (2017) 1646-1649.

[6] X. Jiang, J.M. Wang, Y. Zhang, Z. Chen, Y.M. Zhu, S.J. Ji, Palladium-Catalyzed Formylation of Aryl Halides with tert-Butyl Isocyanide, Organic Letters 16 (2014) 3492-3495.

[7] H. Huang, C. Yu, X. Li, Y. Zhang, Y. Zhang, X. Chen, P.S. Mariano, H. Xie, W. Wang, Synthesis of Aldehydes by Organocatalytic Formylation Reactions of Boronic Acids with Glyoxylic Acid, Angewandte Chemie-International Edition 56 (2017) 8201-8205.

[8] L.J. Goossen, B.A. Khan, T. Fett, M. Treu, Low-Pressure Hydrogenation of Arenecarboxylic Acids to Aryl Aldehydes, Advanced Synthesis & Catalysis 352 (2010) 2166-2170.

[9] X. Wang, R. Liu, Y. Jin, X. Liang, TEMPO/HCl/NaNO2 catalyst: A transition-metal-free approach to efficient aerobic oxidation of alcohols to aldehydes and ketones under mild conditions, Chemistry-a European Journal 14 (2008) 2679-2685.

[10] A. Wang, H.F. Jiang, Palladium-Catalyzed Direct Oxidation of Alkenes with Molecular Oxygen: General and Practical Methods for the Preparation of 1,2-Diols, Aldehydes, and Ketones, Journal of Organic Chemistry 75 (2010) 2321-2326.

[11] H. Huang, X.M. Li, C.G. Yu, Y.T. Zhang, P.S. Mariano, W. Wang, Visible-Light-Promoted Nickel- and Organic-Dye-Cocatalyzed Formylation Reaction of Aryl Halides and Triflates and Vinyl Bromides with Diethoxyacetic Acid as a Formyl Equivalent, Angewandte Chemie-International Edition 56 (2017) 1500-1505.

[12] G.L. Sun, X. Lv, Y.N. Zhang, M. Lei, L.H. Hu, Palladium-Catalyzed Formylation of Aryl Iodides with HCOOH as CO Source, Organic Letters 19 (2017) 4235-4238.
